# Supplementary material for: Individually unique, fixed stripe configurations of Octopus chierchiae allow for photoidentification in long-term studies
Source: PLoS One. 2023 Apr 12;18(4):e0265292. doi: 10.1371/journal.pone.0265292 (PMC10096297; doi:10.1371/journal.pone.0265292)
Supplement: S3 File — (PDF) [file pone.0265292.s003.pdf]

Octopus Chierchiae Photo identification Survey

Each of the following questions displays two photos.

Please mark "Match" if you think the photos are of the same individual or "No Match" if you think they are two different animals.

(Note: the image may be taken from different angles and the animals can distort their bodies and colors. Some images have been rotated to show animals in the same orientation)

* Required

1. 1 *

1 point

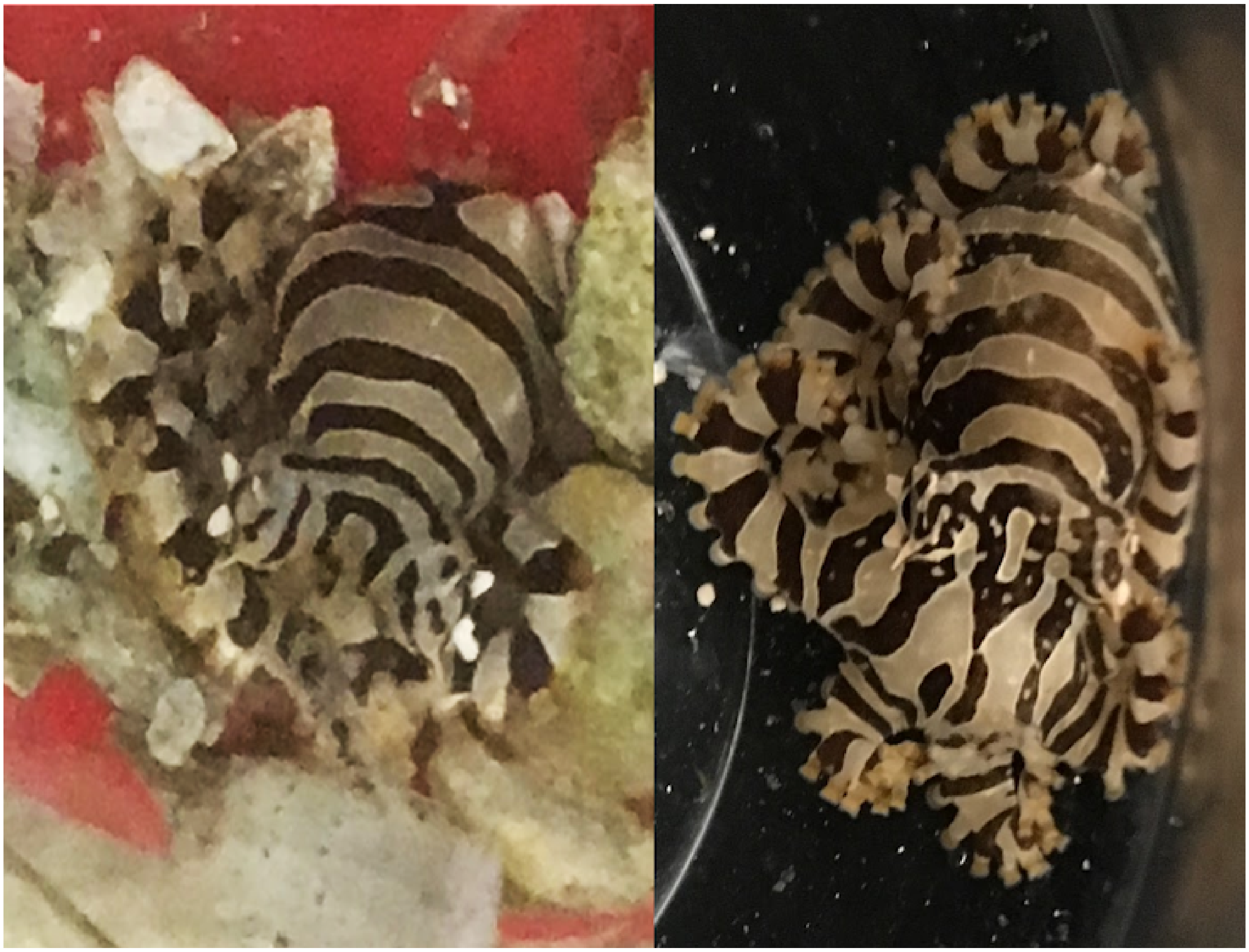

Mark only one oval.

- Match
- No Match

2. 2 *

1 point

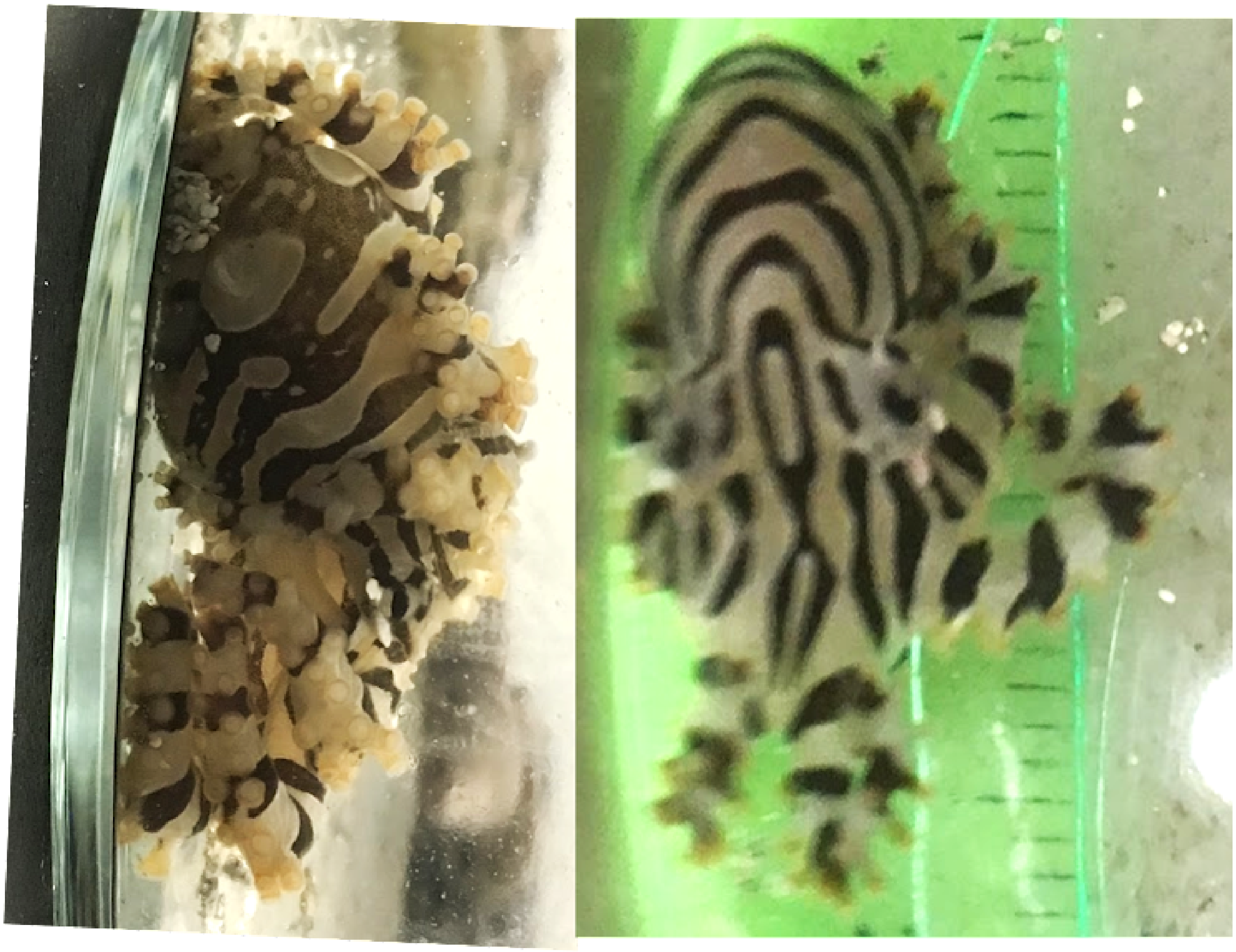

Mark only one oval.

- ( ) Match
- ( ) No Match

3. 3 *

1 point

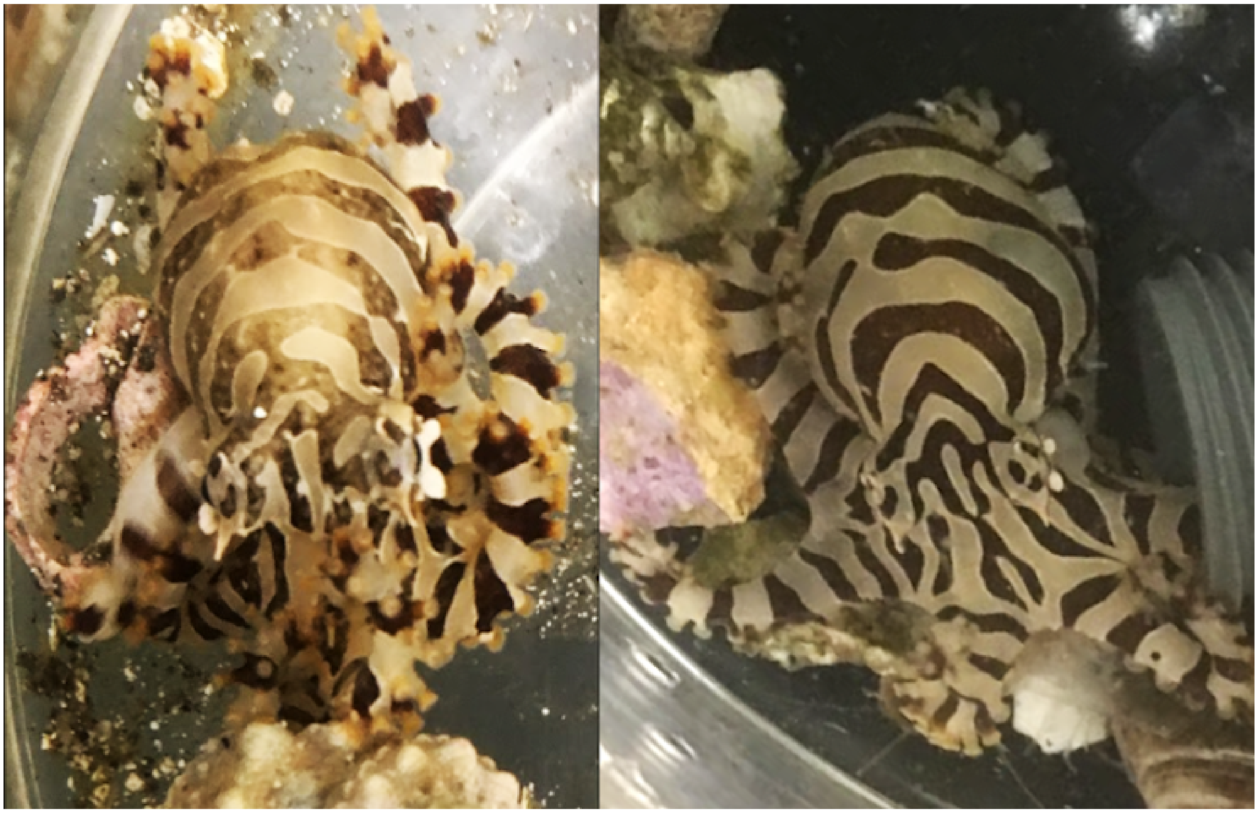

Mark only one oval.

- Match
- No Match

4. 4 * 1 point

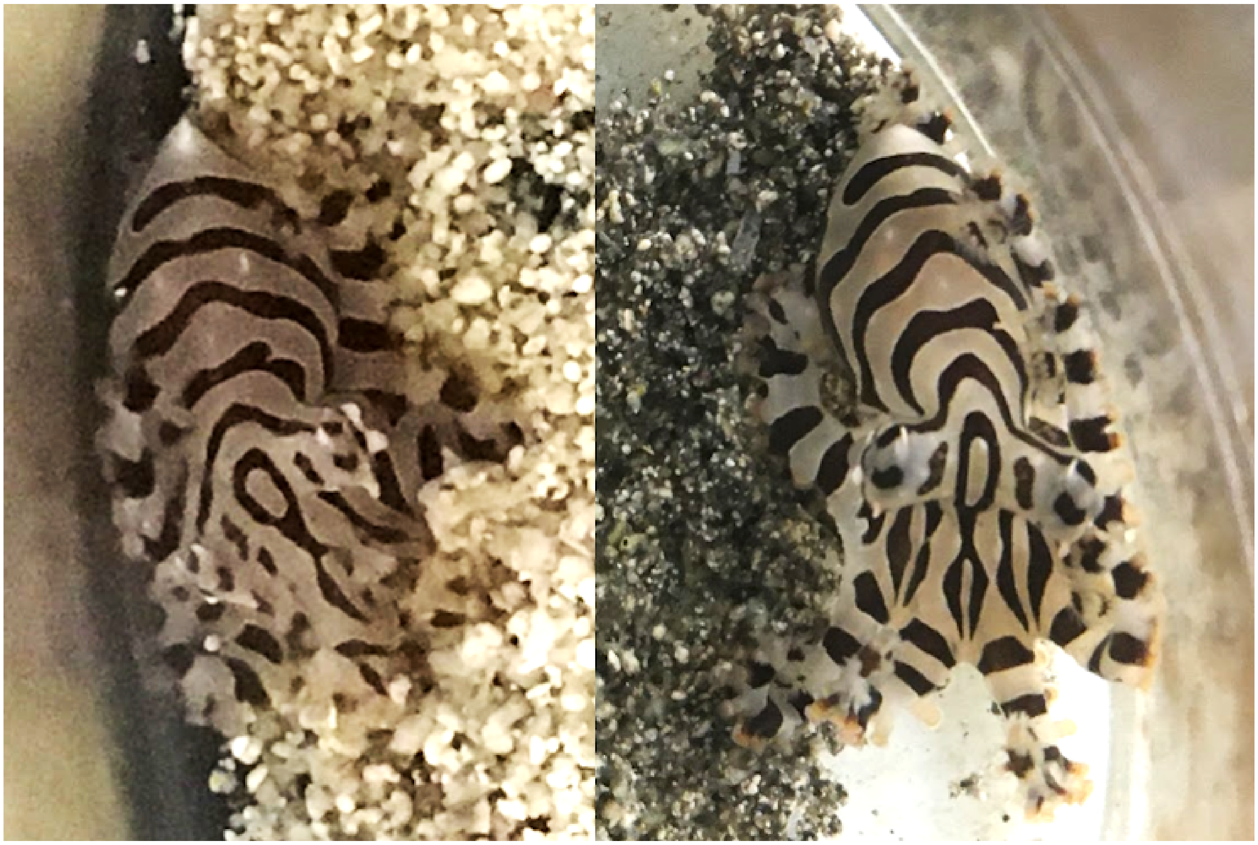

Mark only one oval.

- Match
- No Match

5. 5 *

1 point

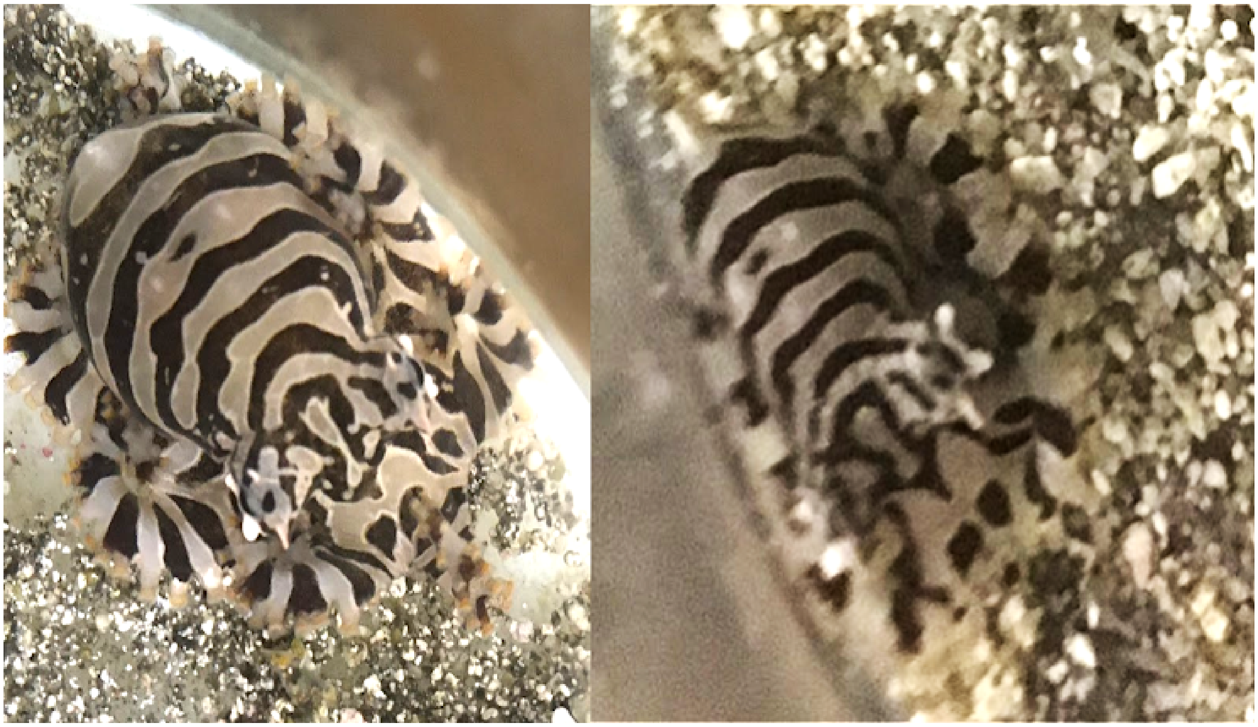

Mark only one oval.

- ( ) Match
- ( ) No Match

6. 6 *

1 point

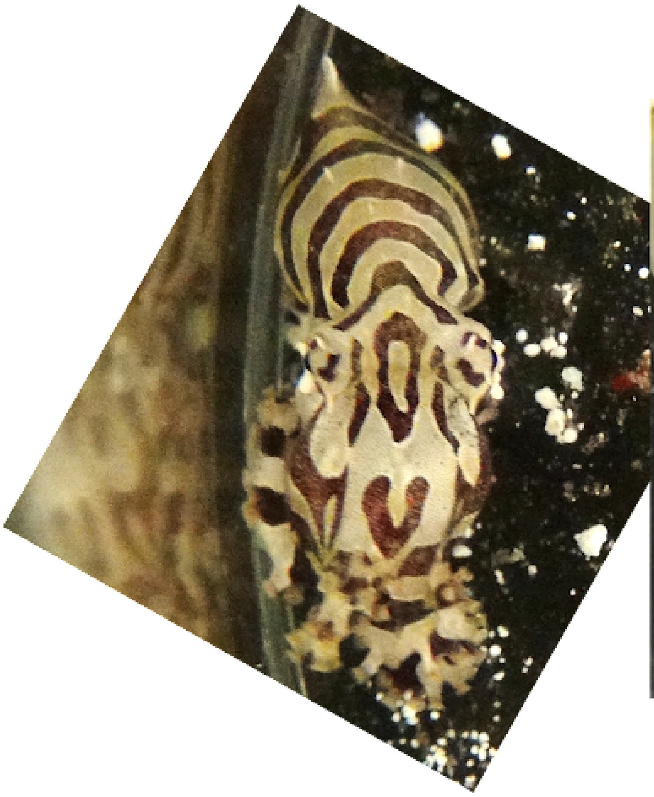

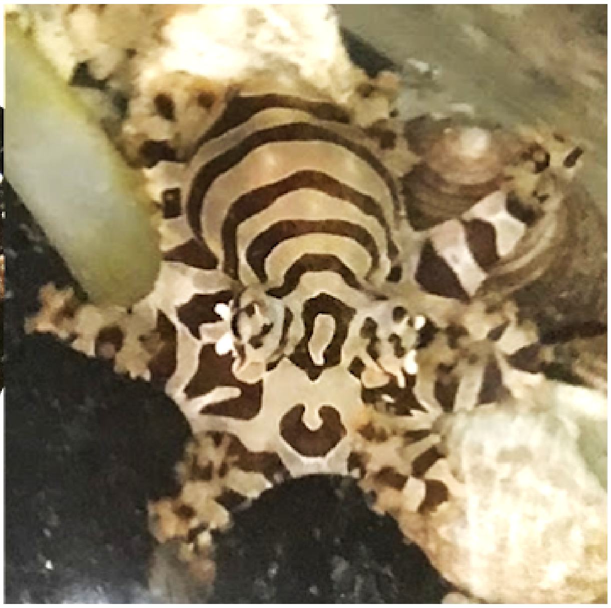

Mark only one oval.

- ( ) Match
- ( ) No Match

7. 7 * 1 point

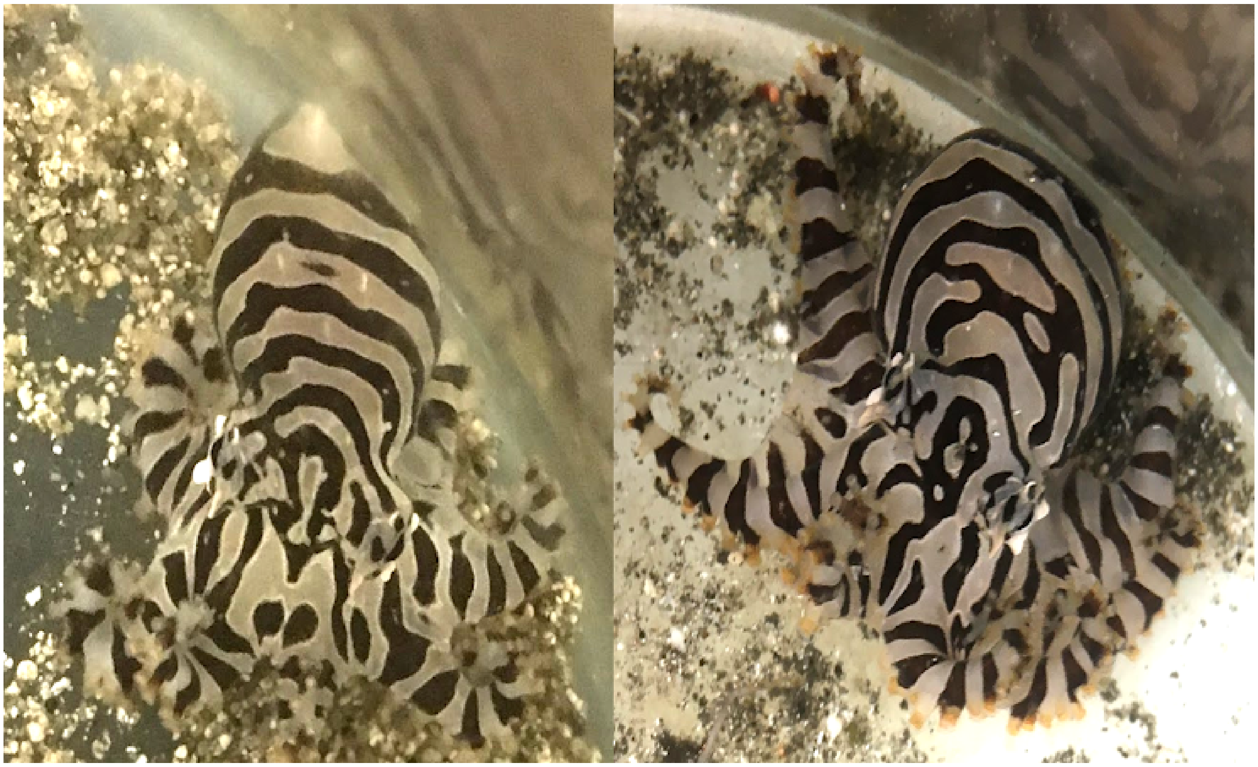

Mark only one oval.

- Match
- No Match

8. 8 *

1 point

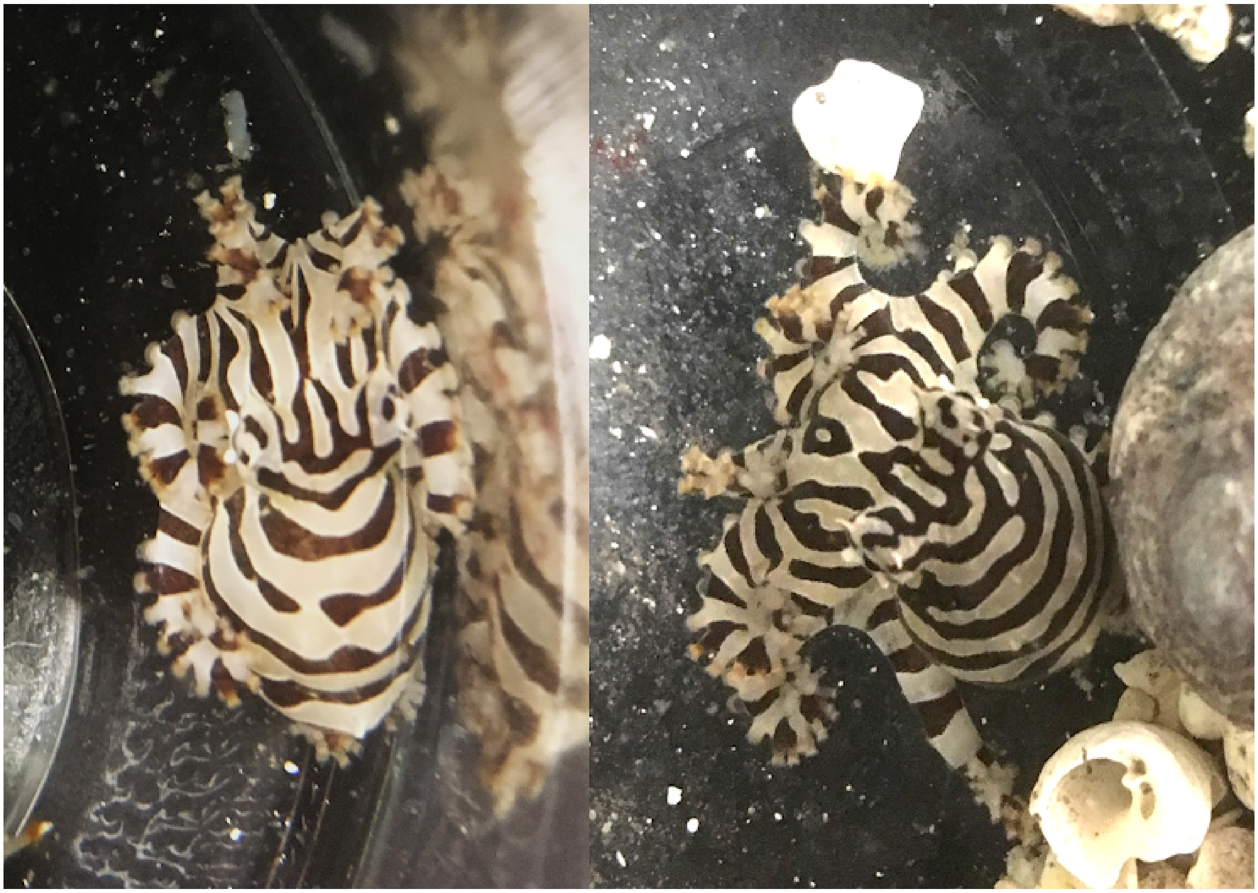

Mark only one oval.

- Match
- No Match

9. 9 *

1 point

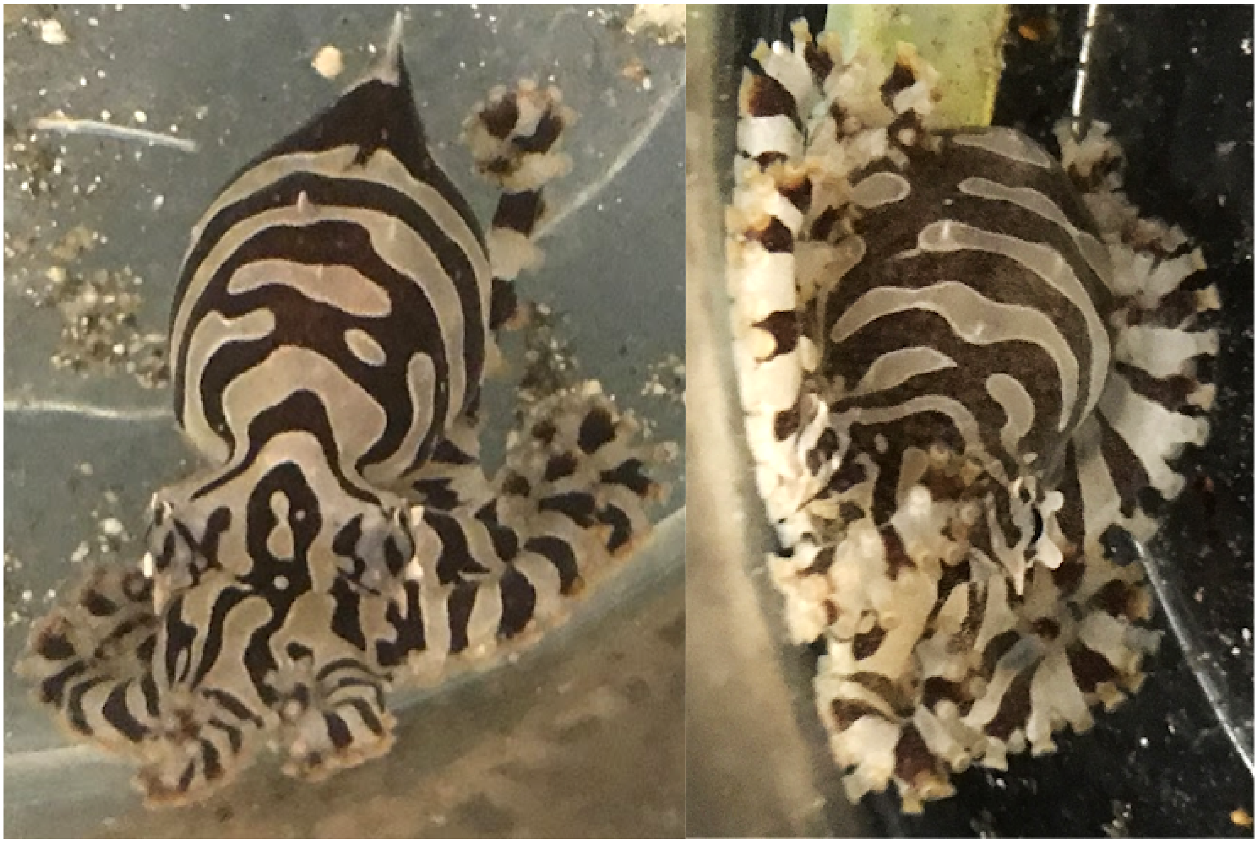

Mark only one oval.

- Match
- No Match

10. 10 * 1 point

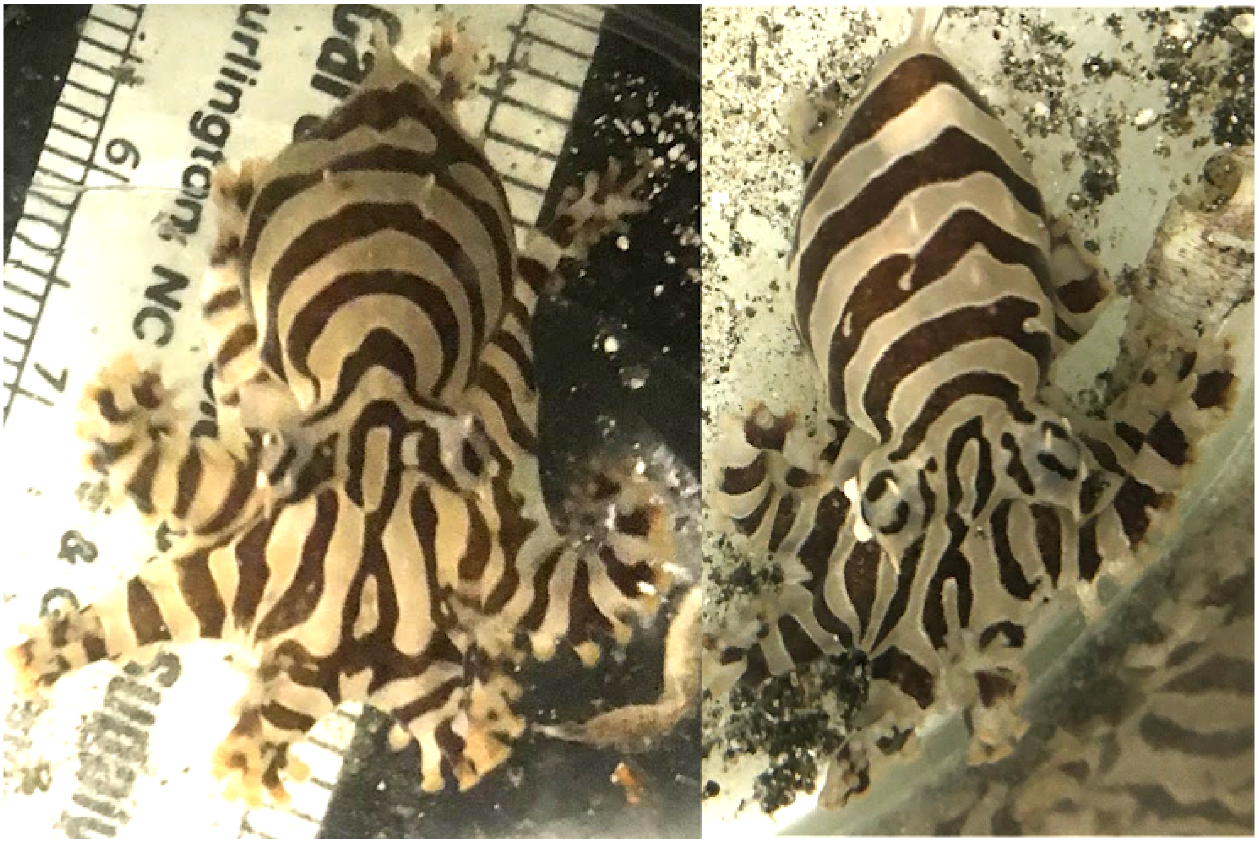

Mark only one oval.

- Match
- No Match

11. 11 * 1 point

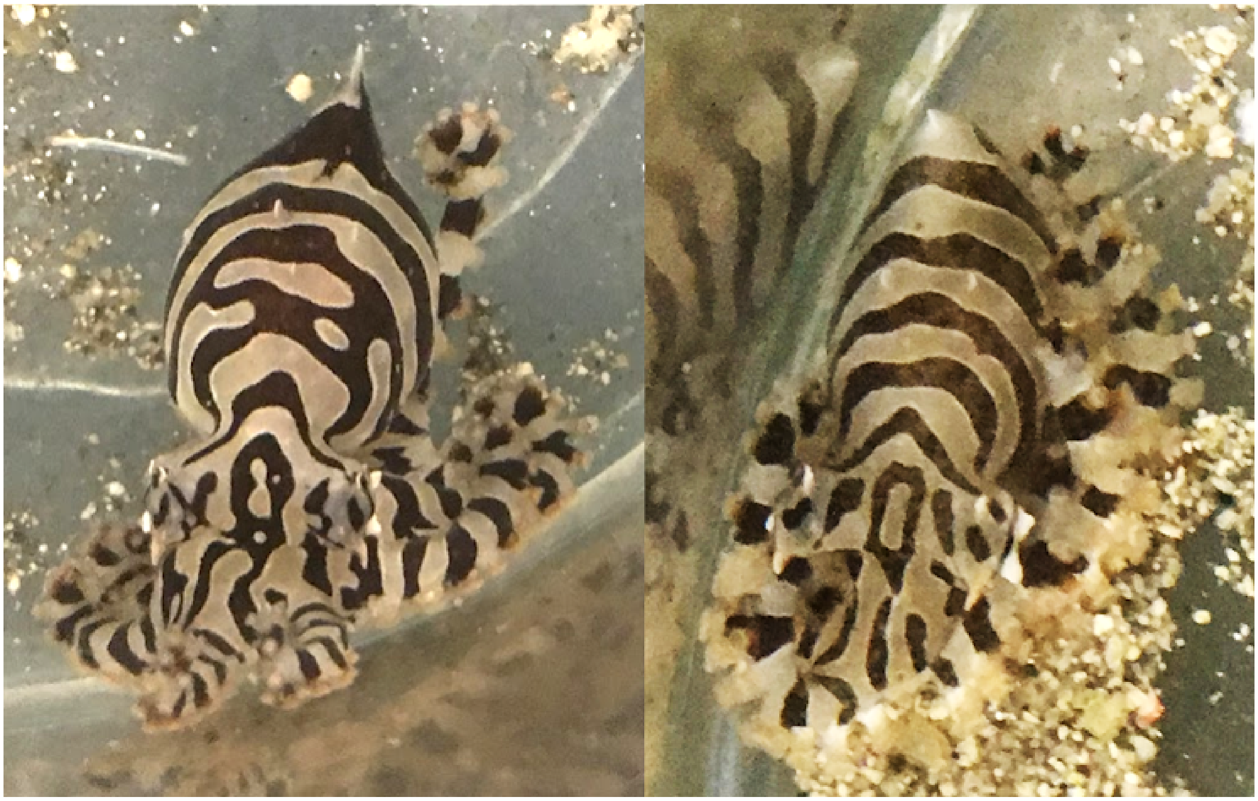

Mark only one oval.

- Match
- No Match

12. 12 *

1 point

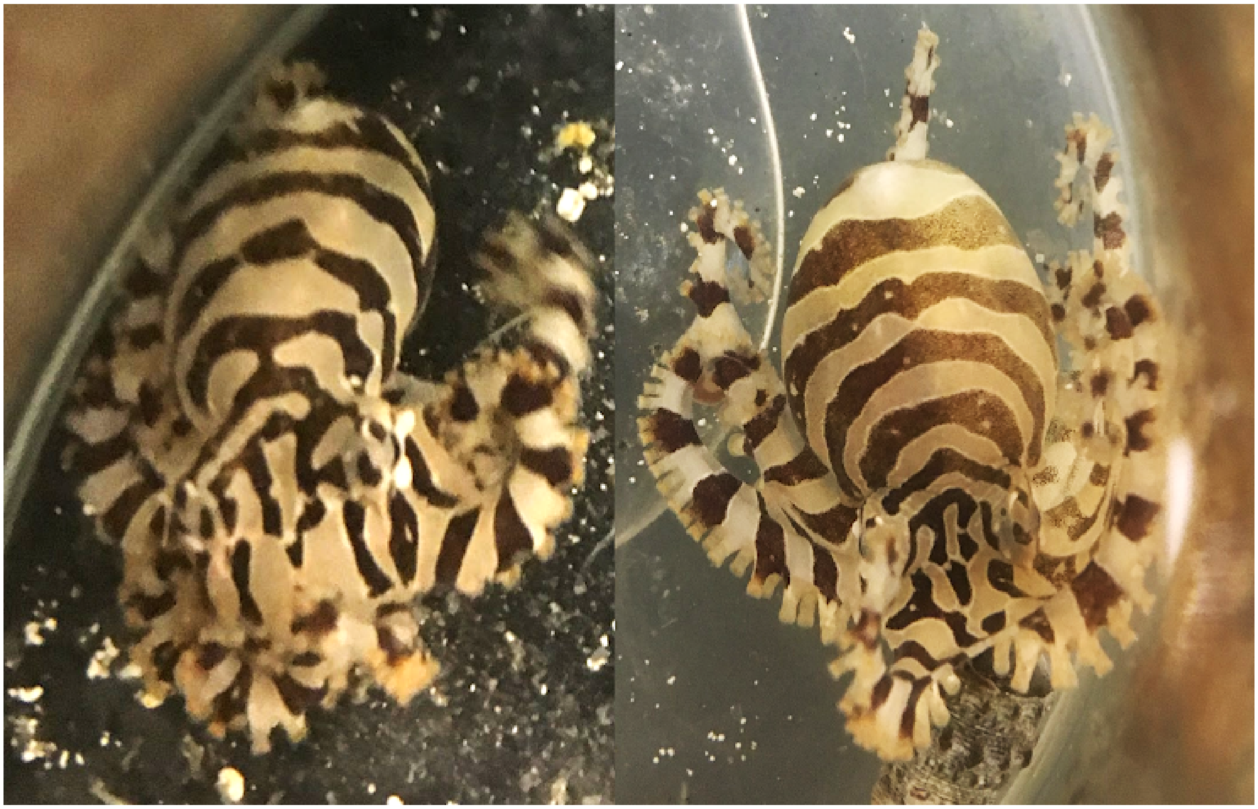

Mark only one oval.

- Match
- No Match

13. 13 *

1 point

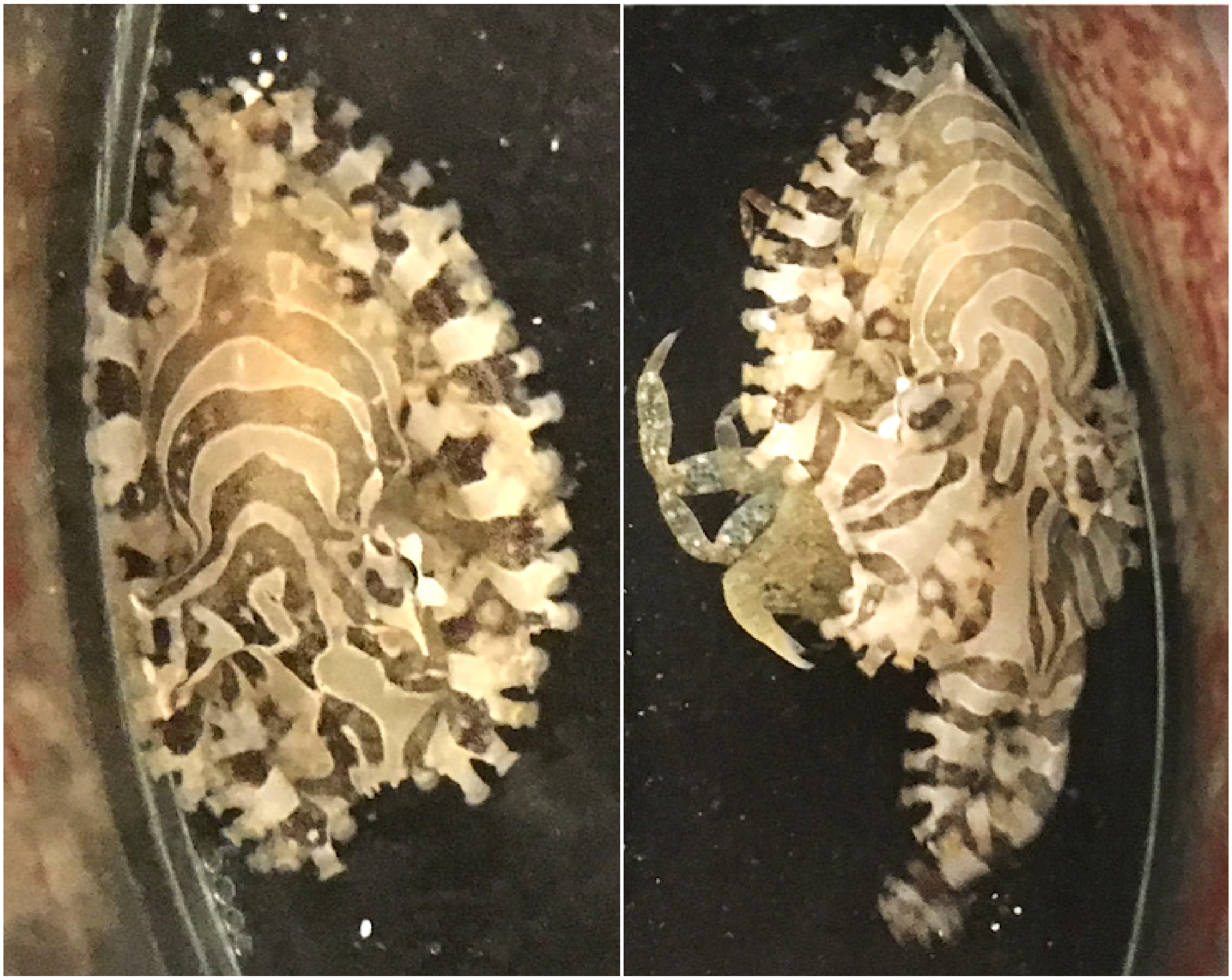

Mark only one oval.

- Match
- No Match

14. 14 *

1 point

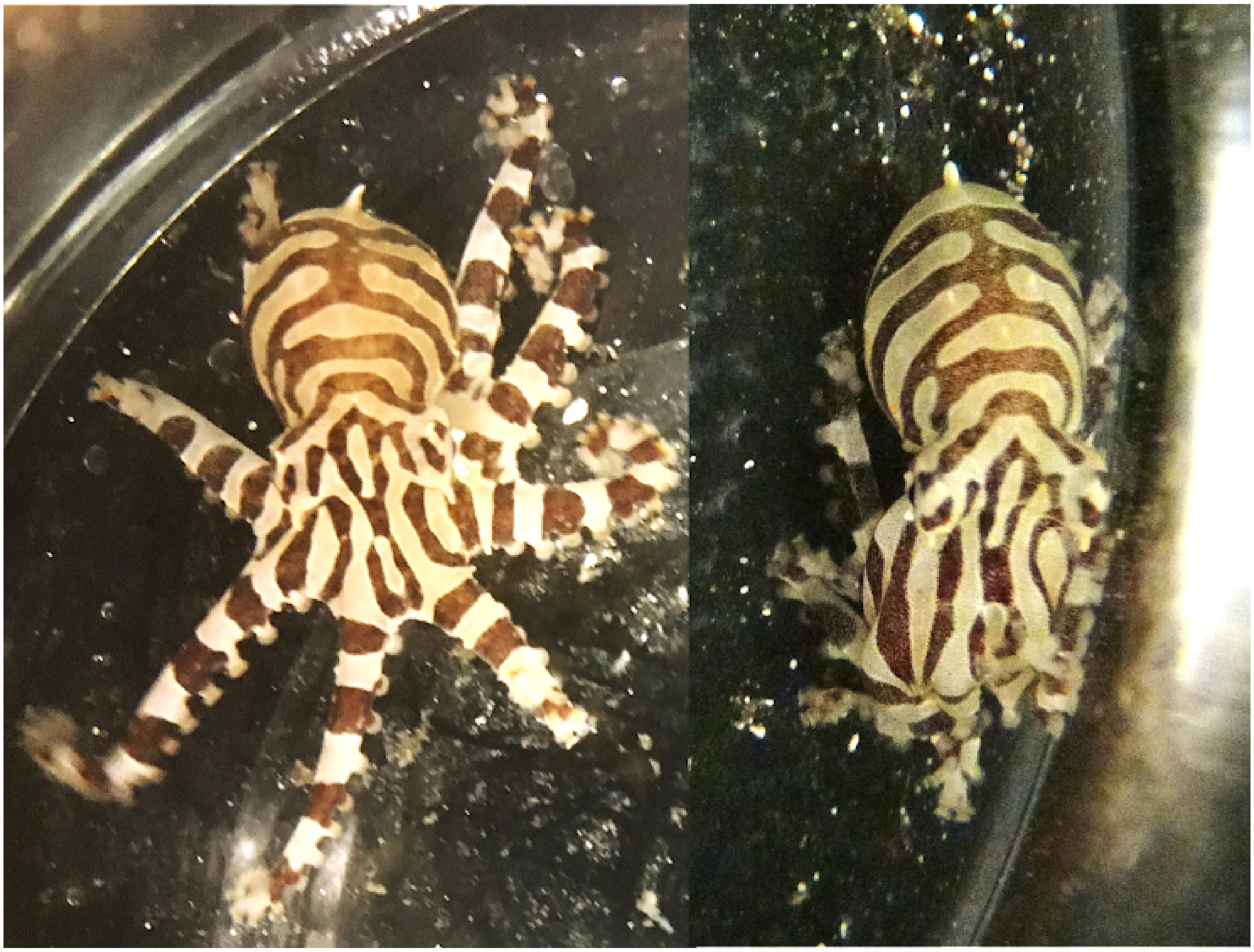

Mark only one oval.

- ◯ Match
- ◯ No Match

15. 15 *

1 point

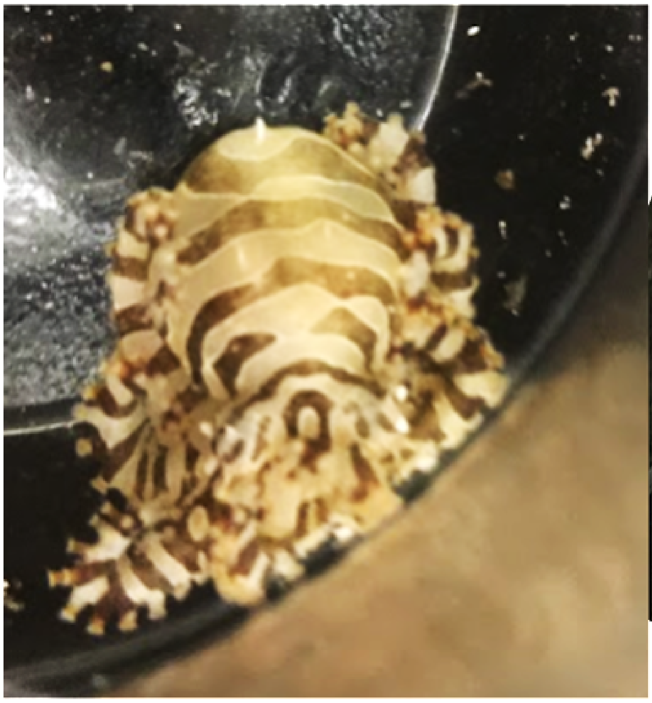

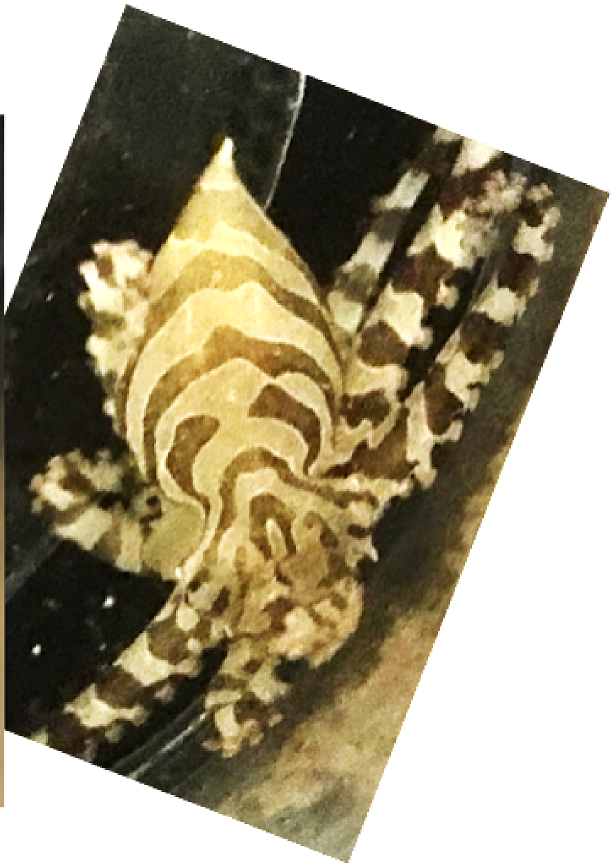

Mark only one oval.

- Match
- No Match

16. 16 *

1 point

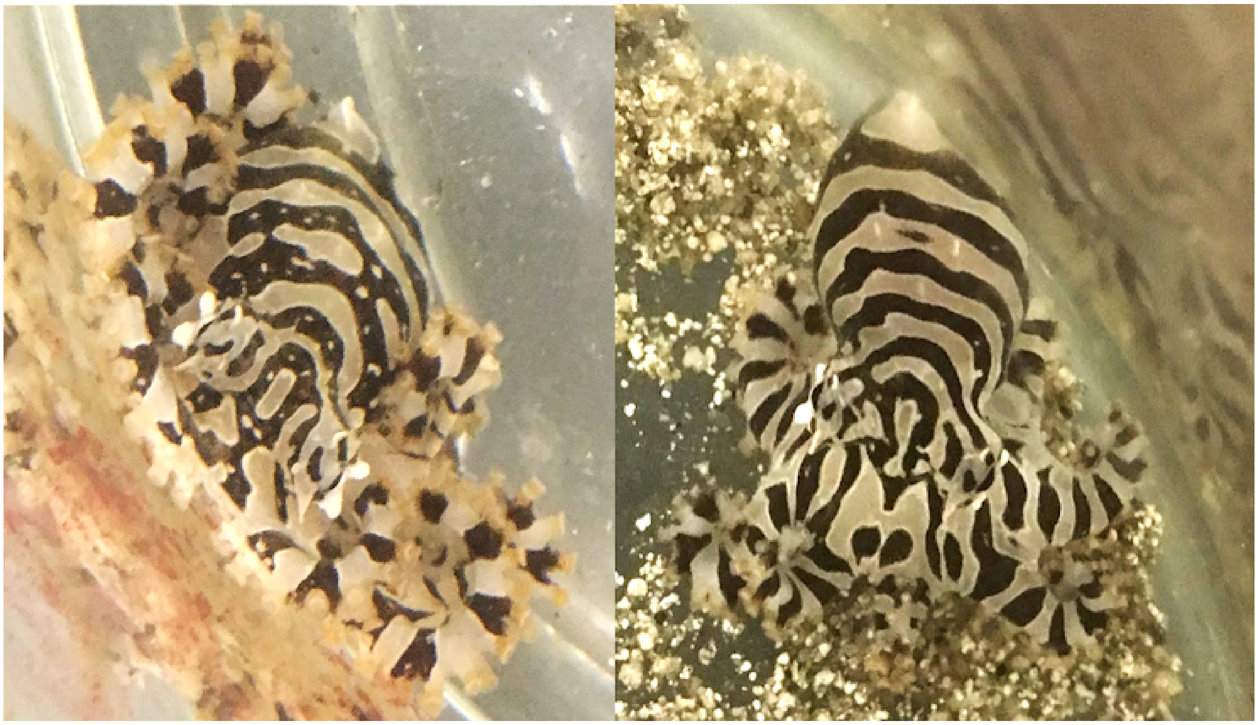

Mark only one oval.

- Match
- No Match

17. 17 * 1 point

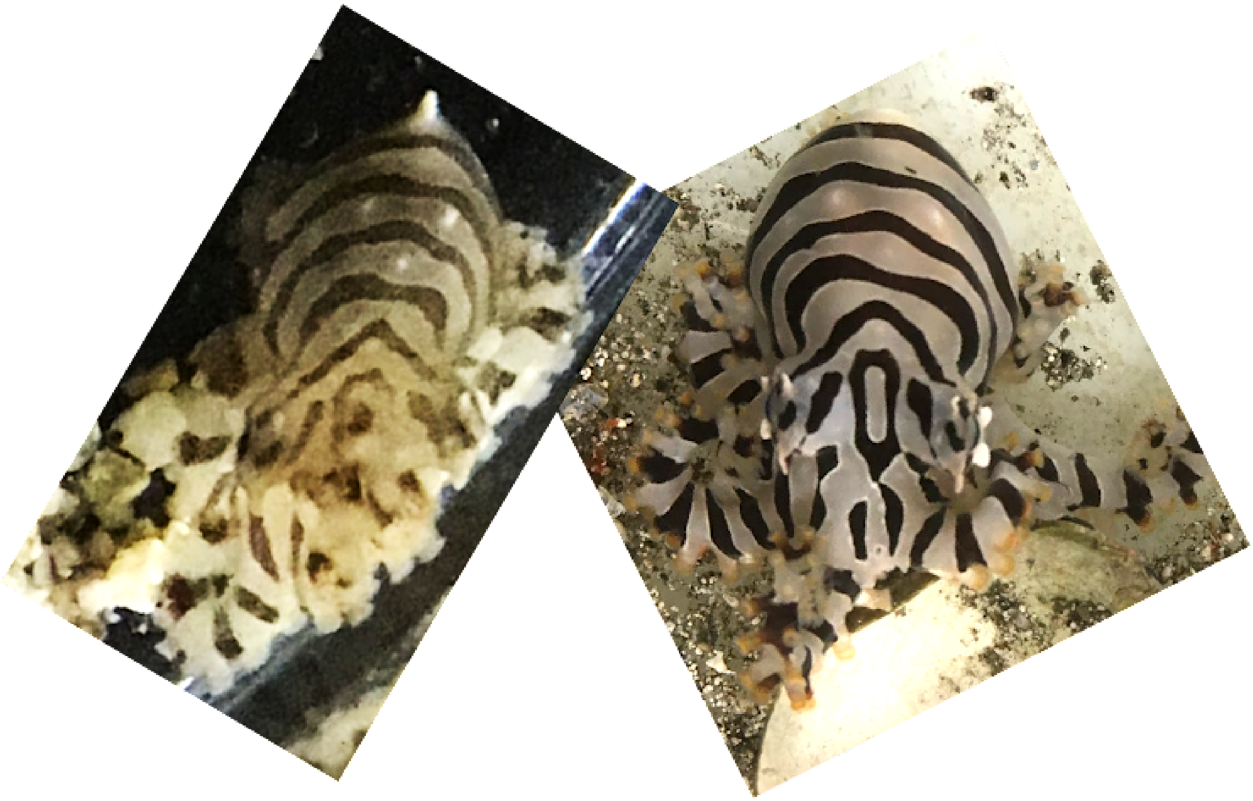

Mark only one oval.

- Match
- No Match

18. 18 *

1 point

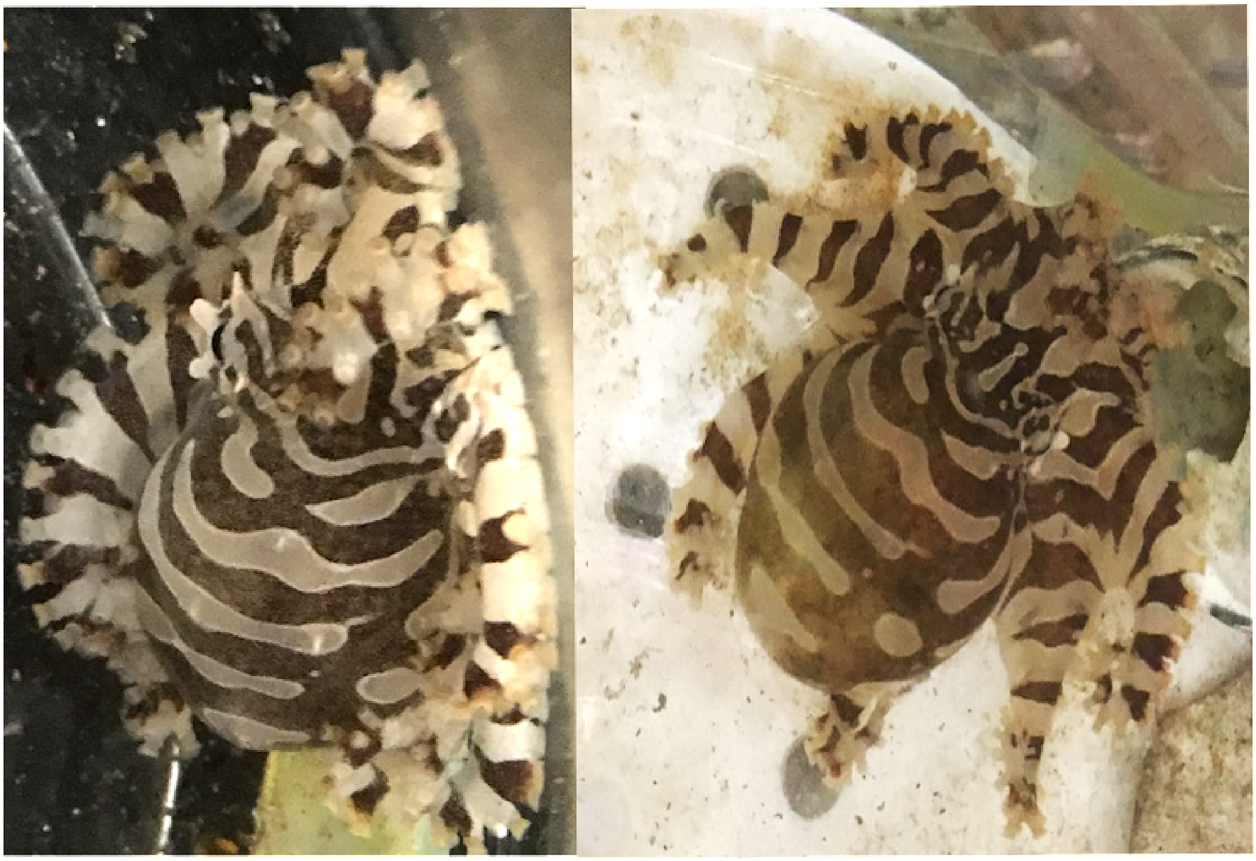

Mark only one oval.

- Match
- No Match

19. 19 *

1 point

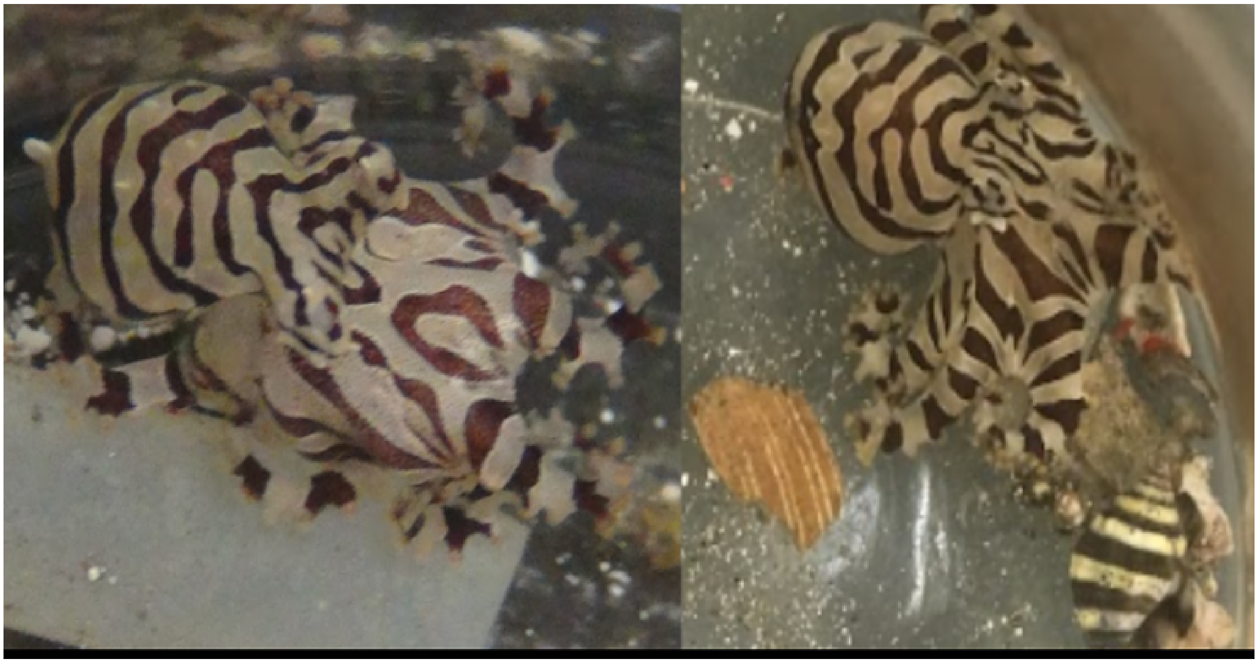

Mark only one oval.

- Match
- No Match

20. 20 *

1 point

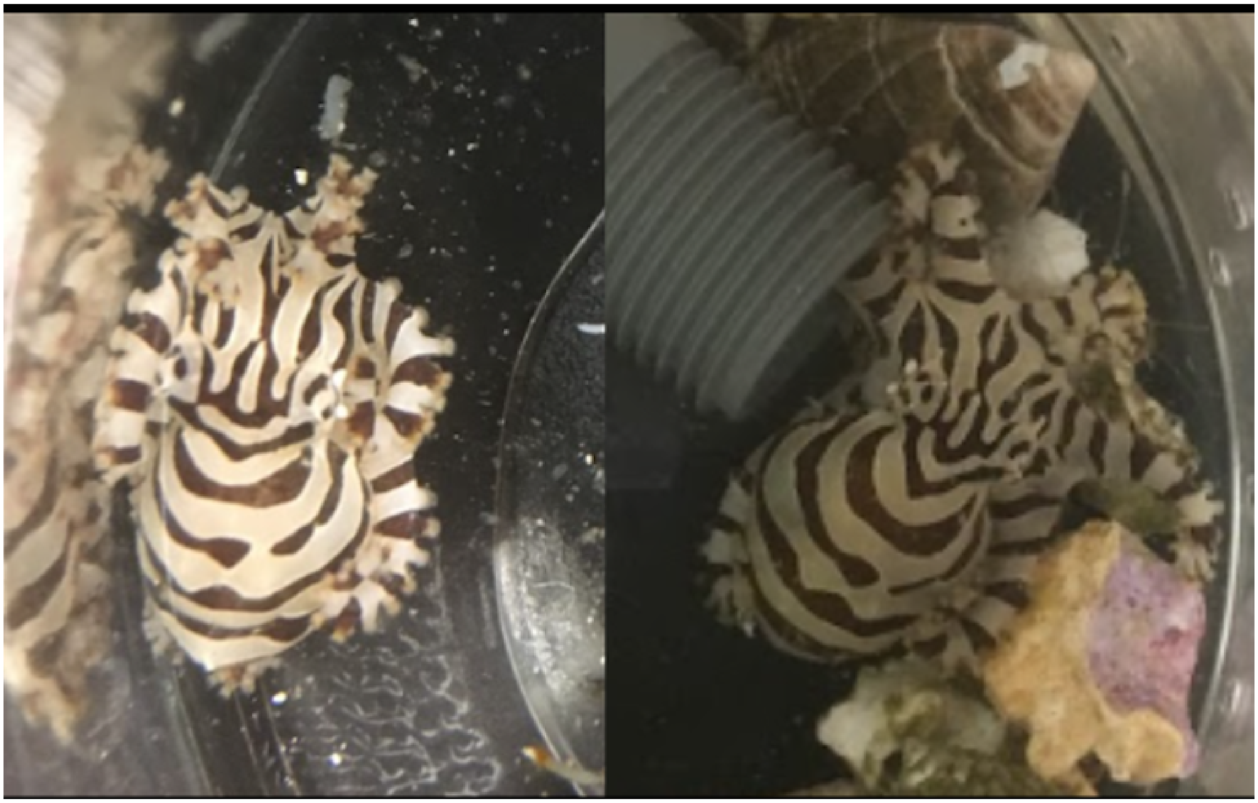

Mark only one oval.

- Match
- No Match

This content is neither created nor endorsed by Google.

Google Forms
