# Supplementary material for: Individually unique, fixed stripe configurations of Octopus chierchiae allow for photoidentification in long-term studies
Source: PLoS One. 2023 Apr 12;18(4):e0265292. doi: 10.1371/journal.pone.0265292 (PMC10096297; doi:10.1371/journal.pone.0265292)
Supplement: S4 File — (PDF) [file pone.0265292.s004.pdf]

Preliminary Surveys and Discussion

We created two preliminary surveys following the methods of Huffard et. al. (2008) from our photobank, solicited participants via social media, and collected responses via Google Forms. Survey A consisted of a series of 50 slides, each showing two photographs (taken from 0 days to 5 months apart) of *O. chierchiae* individuals side by side. Participants of Survey A were asked to mark whether the 2 photographs were of the same or different individuals. Survey B consisted of the same series of slides as Survey A, but included pattern traces created in Adobe Illustrator below the photographs.

Our preliminary survey results (S6 Dataset) showed that *O. chierchiae* individuals are distinguishable to a majority of untrained observers. 63.6% and 54.5% of all participants scored above 75% on Surveys A and B respectively. There were five questions on each survey where over 50% of participants answered incorrectly and one question on each where it was an even split between correct and incorrect responses. Upon specific examination of these problematic questions, the increased difficulty could be explained by a combination of subpar photo quality, the animal being in dark morph (thus making its stripes harder to discern), body pattern being obscured in some manner, or the animals being viewed from different angles.

Interestingly, the average score was lower on Survey B where body pattern traces were provided for assistance, perhaps showing variance in how individual participants recognize identifying features. Like the results for *Wunderpus photogenicu*s, false positives were more common than false negatives in both survey groups when photoidentifying *O. chierchiae* (Huffard et. al., 2008). Additionally, we received feedback that a 50 question survey took too long to complete for some people to be interested in participating.

Given these inferences, for the refined version of the survey discussed in the main body of our manuscript, we only included images of animals in the stripe-bar-spot morph with no major obstructions to the stripe patterns on their dorsal mantle surface. Additionally, we shortened the survey to 20 questions and rotated the images so that each slide depicted animals in similar orientations.

The bar charts below present the scores of each participant, illustrated as total score (Blue), False Negative (Red) and False Positive (Orange) when assessing images.

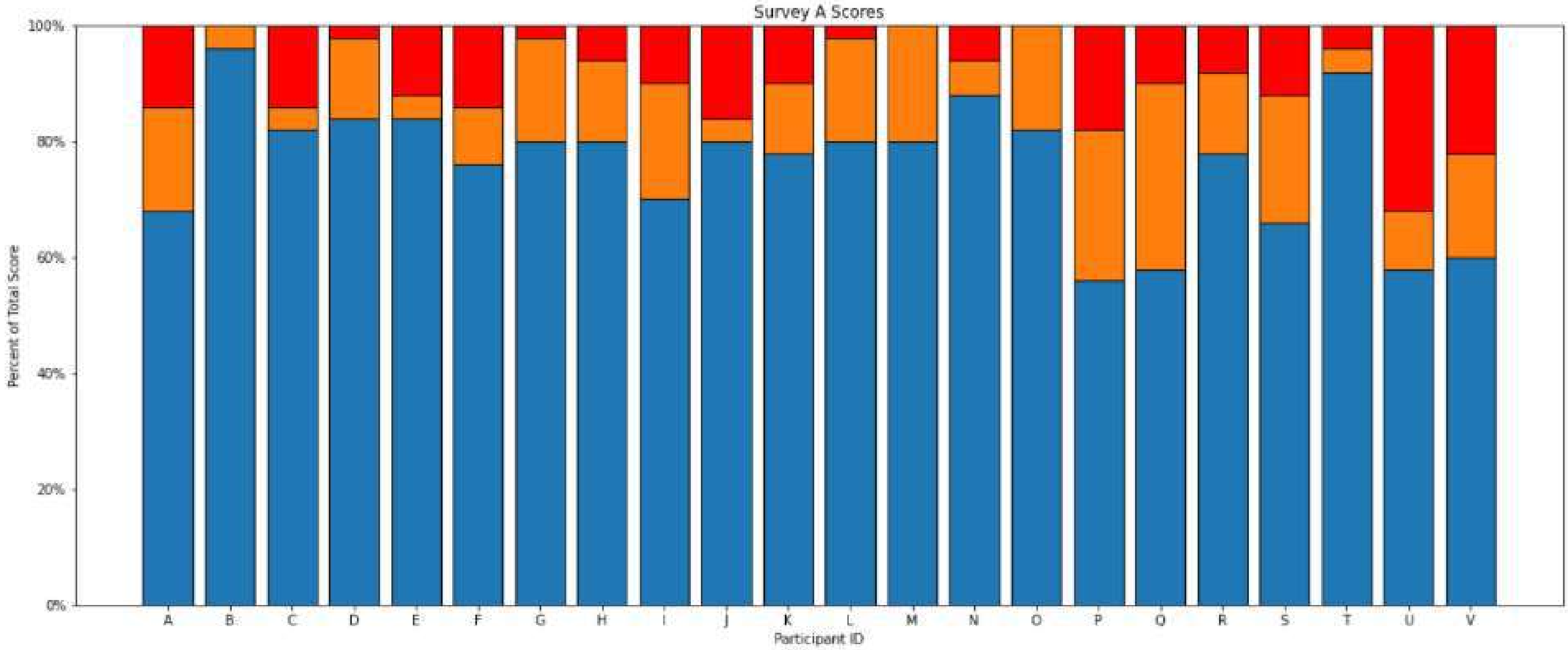
Survey A Scores
Percent of Total Score
100%
80%
60%
40%
20%
0%
A B C D E F G H I J K L M N O P Q R S T U V
Participant ID

Octopus Chierchiae Photo identification Survey A

Each of the following questions displays 2 photos.

Please mark "Match" if you think the photos are of the same individual or "no match" if you think they are two different animals.

(Note: the image may be taken from different angles and the animals can distort their bodies and colors)

1

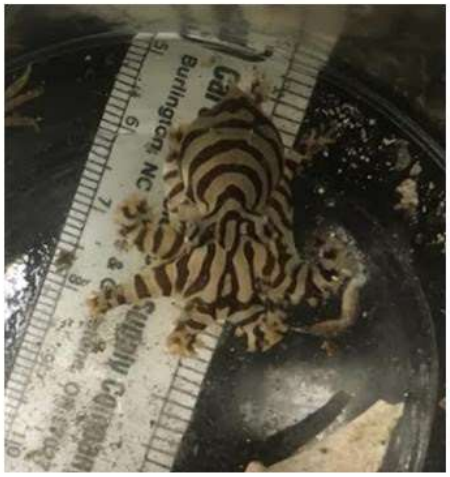

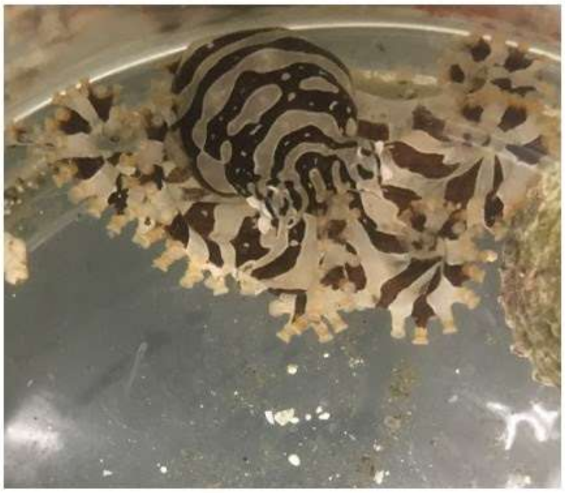

1. *Mark only one oval.*

- match
- no match

2

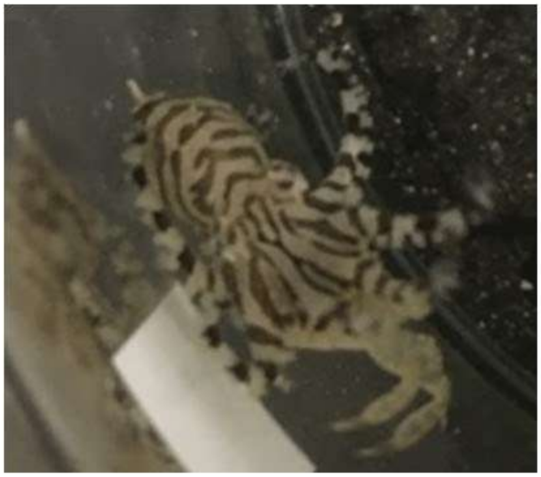

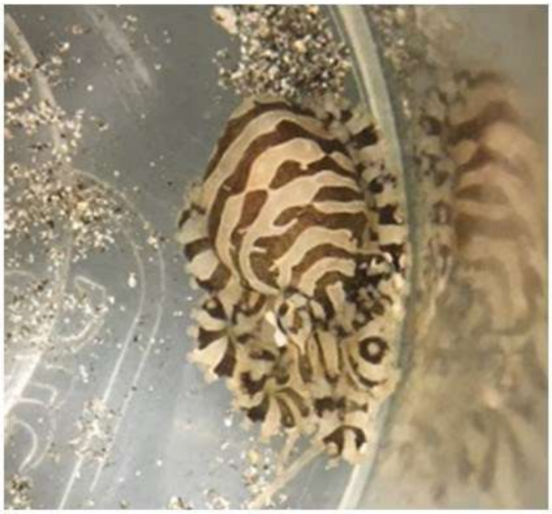

2\. *Mark only one oval.*

- match
- no match

3

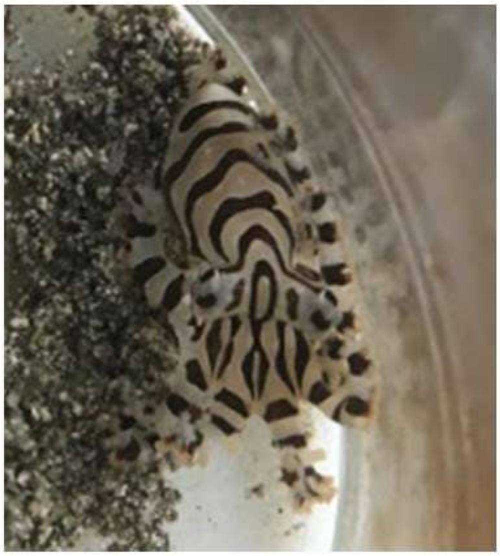

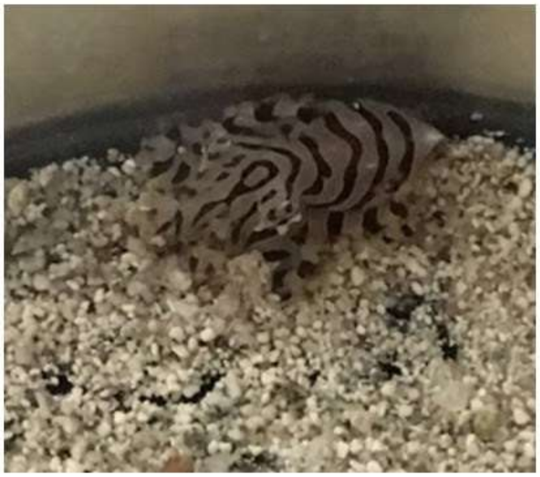

3\.
Mark only one oval.

- match
- no match

4

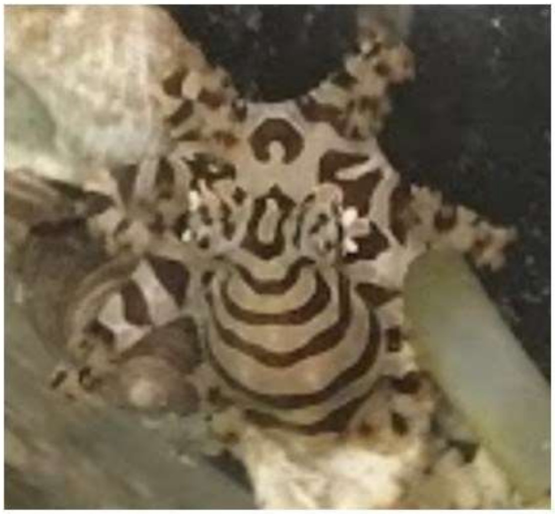

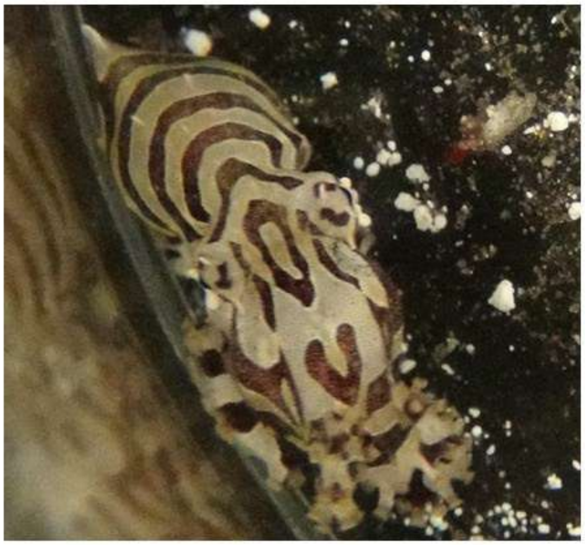

4\. *Mark only one oval.*

- match
- no match

5

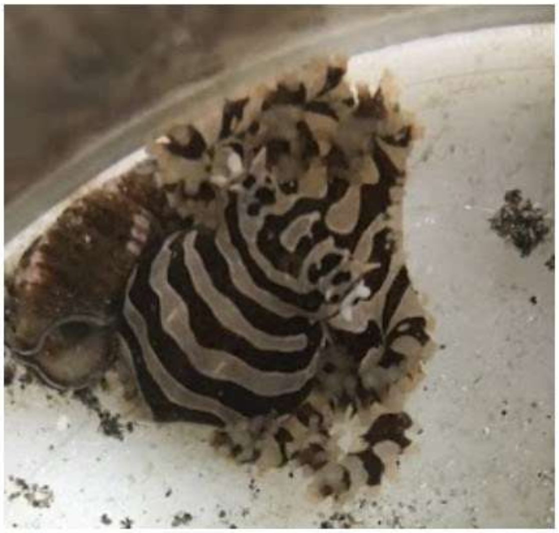

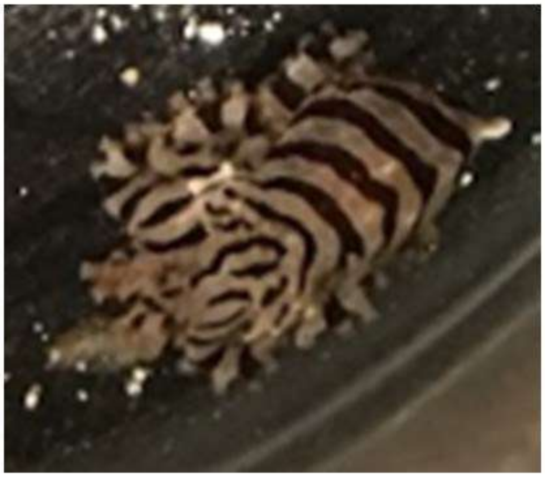

5.
Mark only one oval.

- match
- no match

6

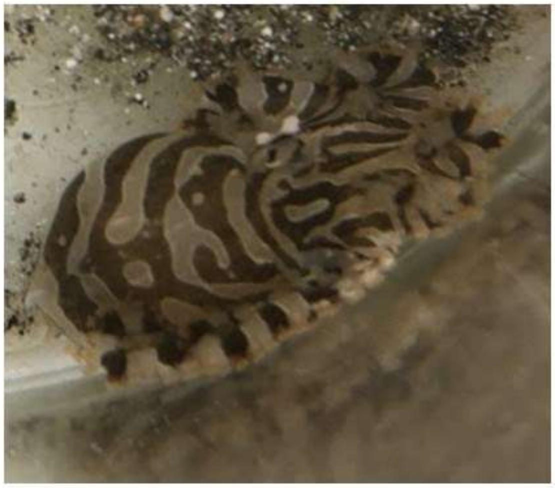

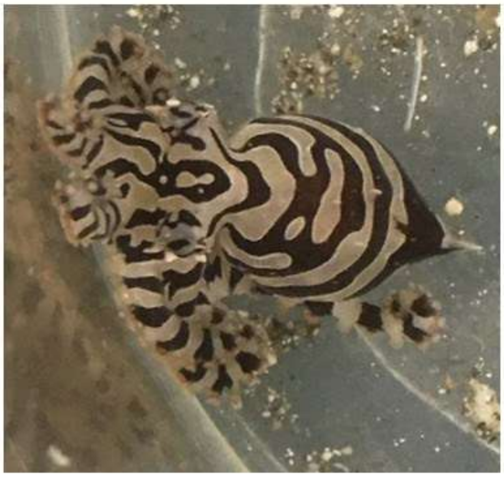

6.
Mark only one oval.

- match
- no match

7

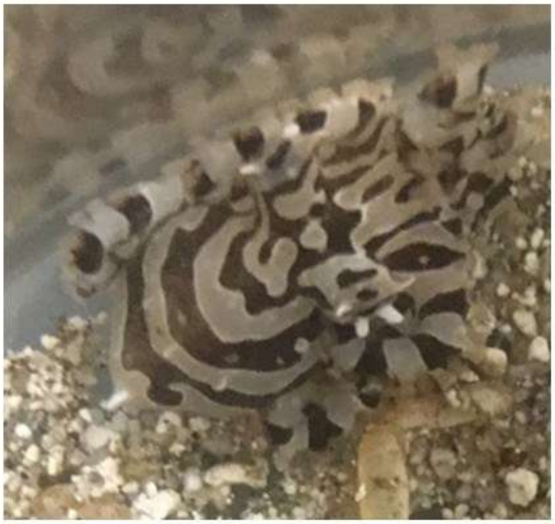

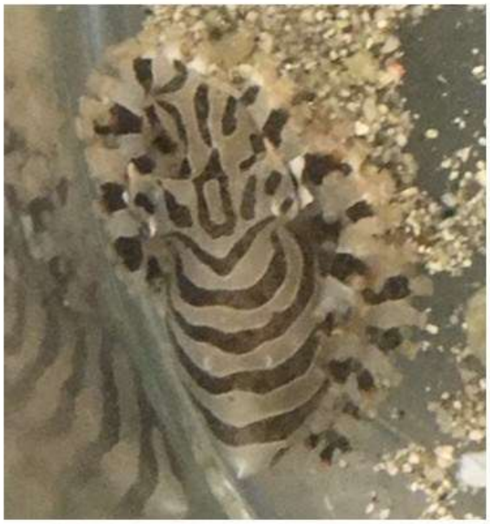

7.
Mark only one oval.

- match
- no match

8

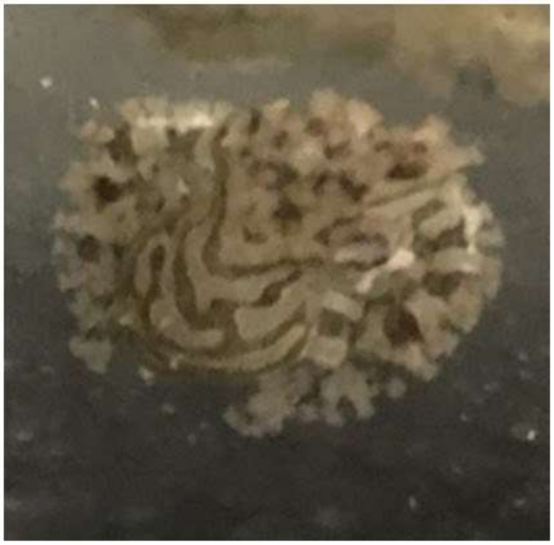

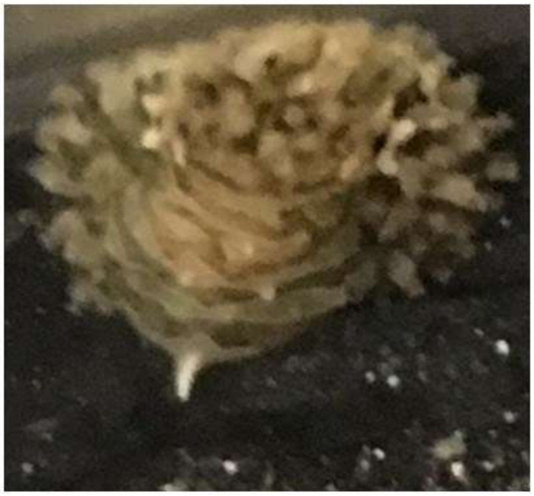

8.
Mark only one oval.

- ( ) match
- ( ) no match

9

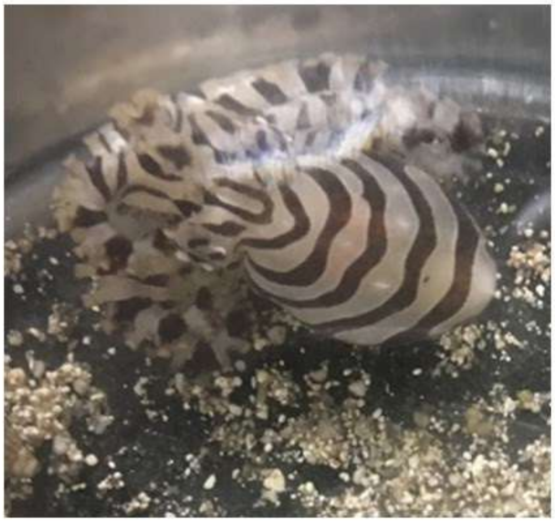

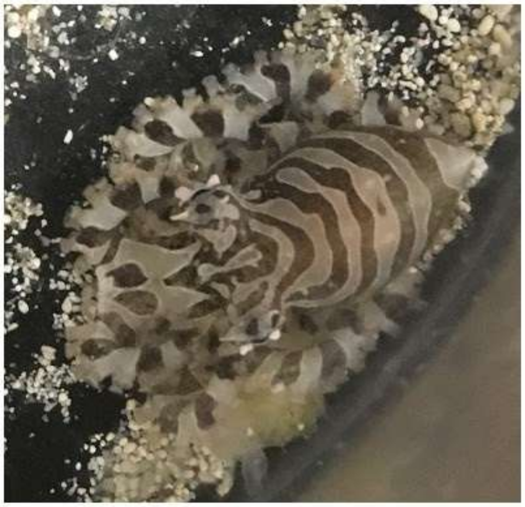

9. *Mark only one oval.*

- match
- no match

10

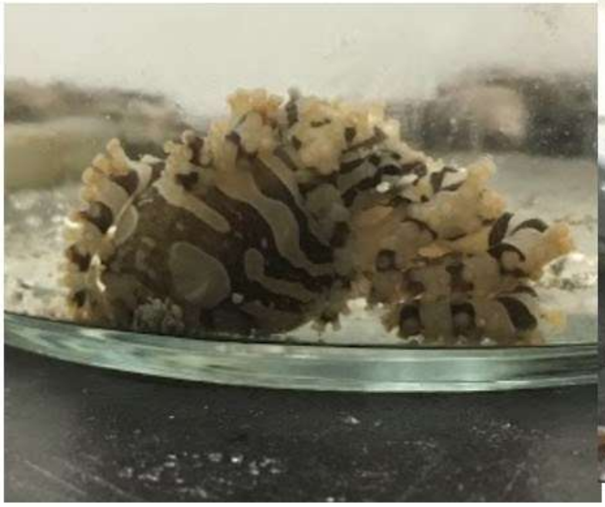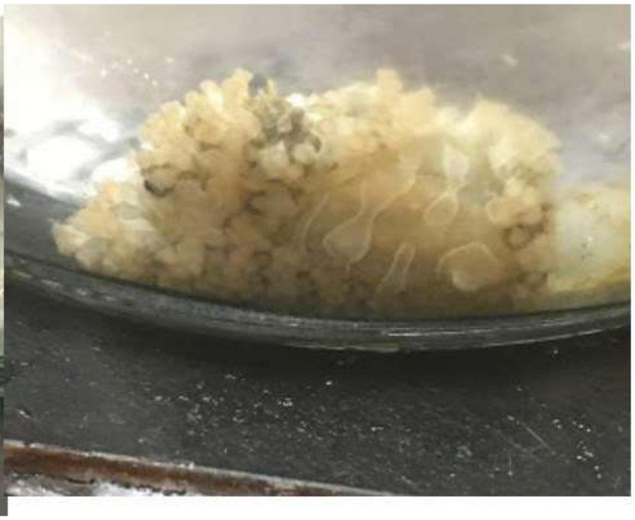

10\. *Mark only one oval.*

- match
- no match

11

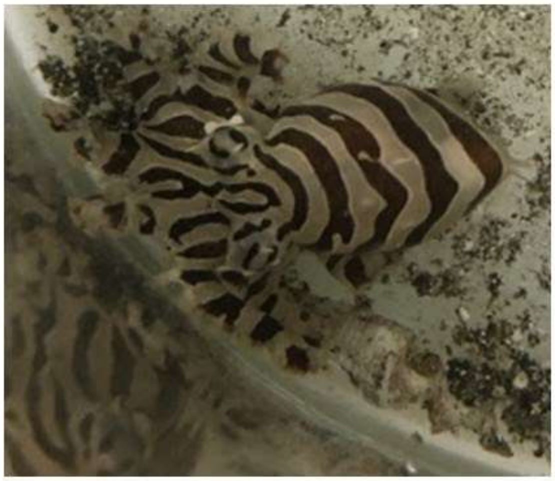

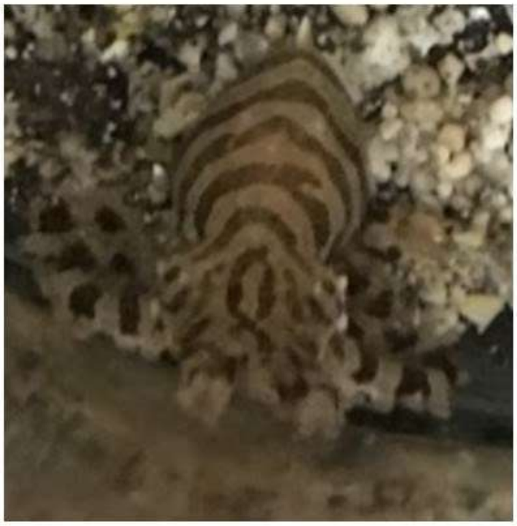

11.

Mark only one oval.

- match
- no match

12

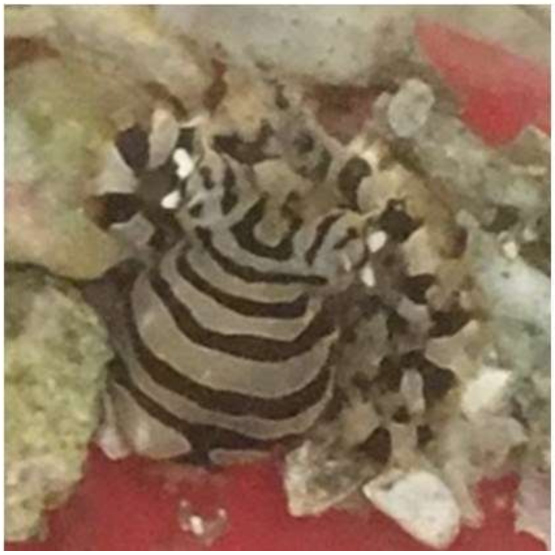

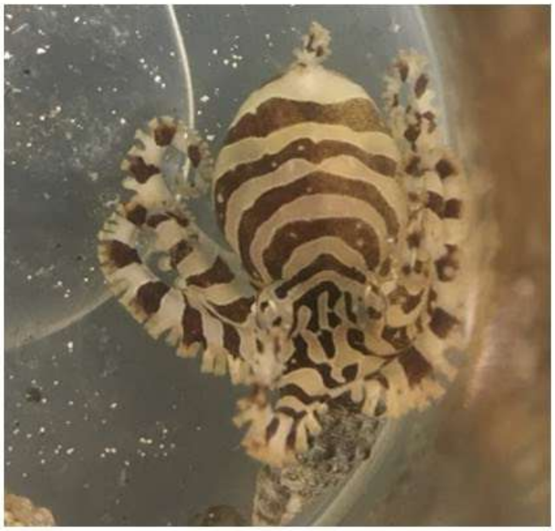

12.
Mark only one oval.

- ( ) match
- ( ) no match

13

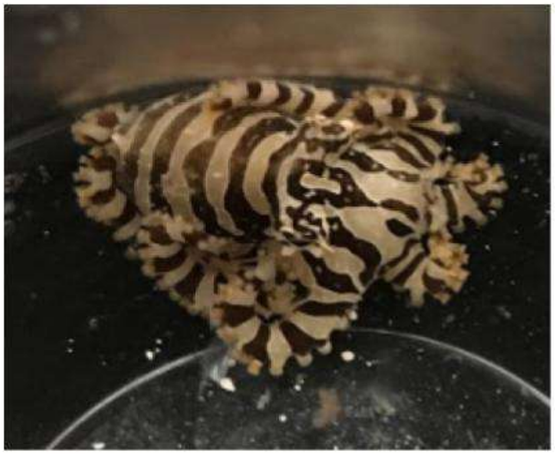

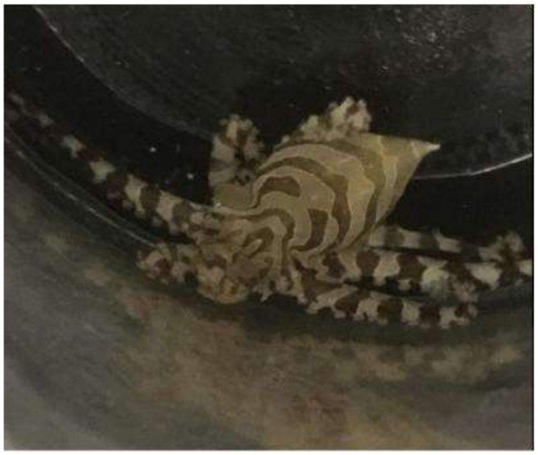

13.

Mark only one oval.

- match
- no match

14

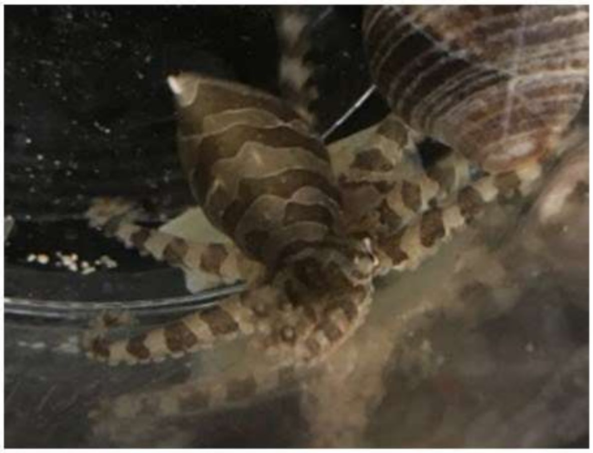

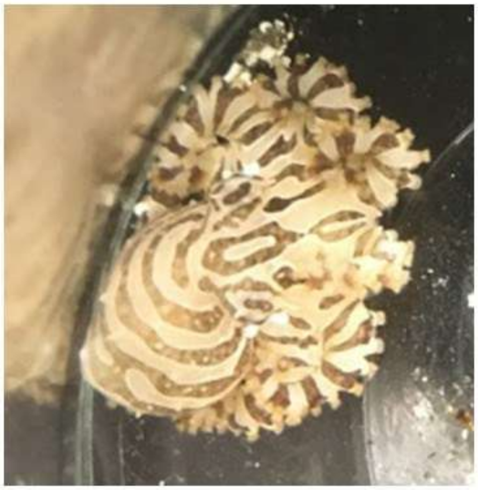

14.

Mark only one oval.

- match
- no match

15

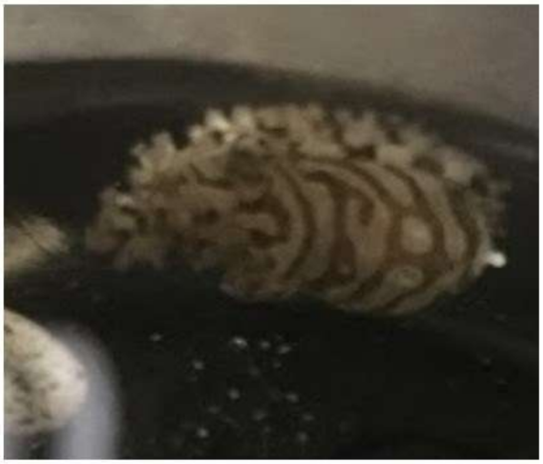

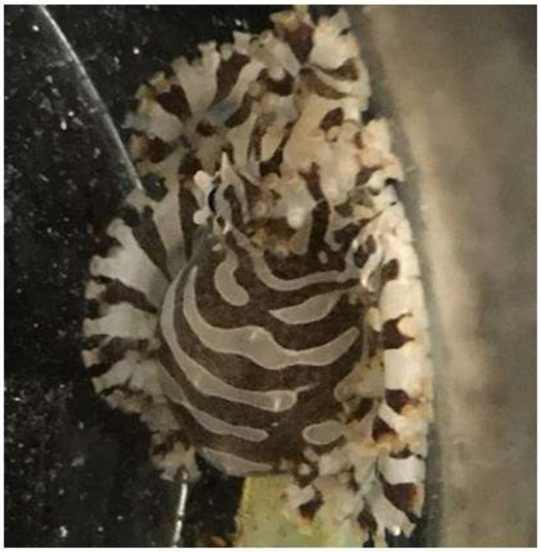

15.
Mark only one oval.

- match
- no match

16

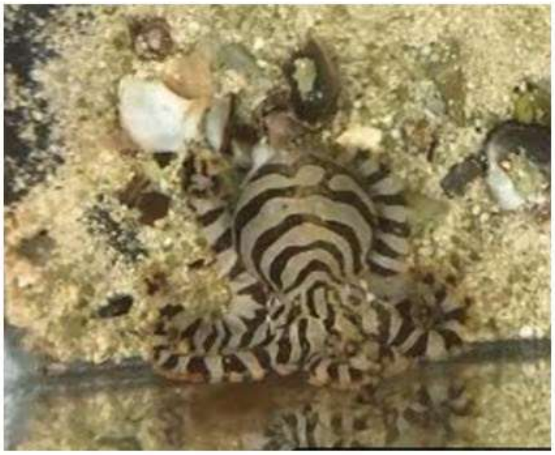

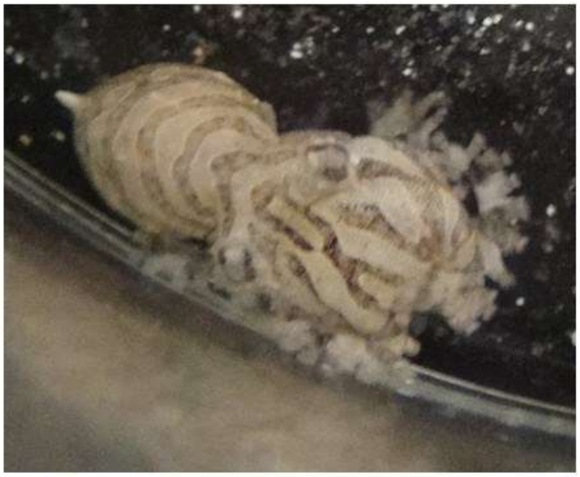

16.

Mark only one oval.

- match
- no match

17

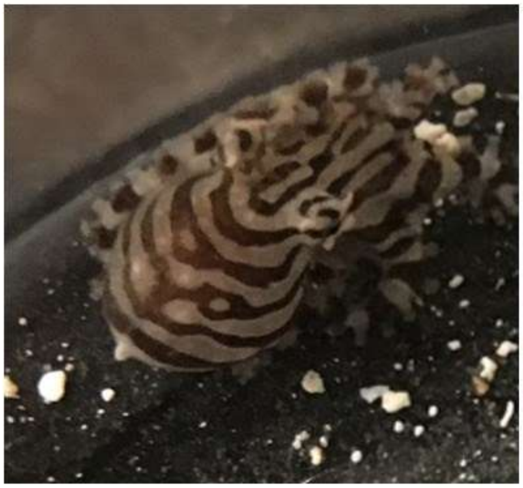

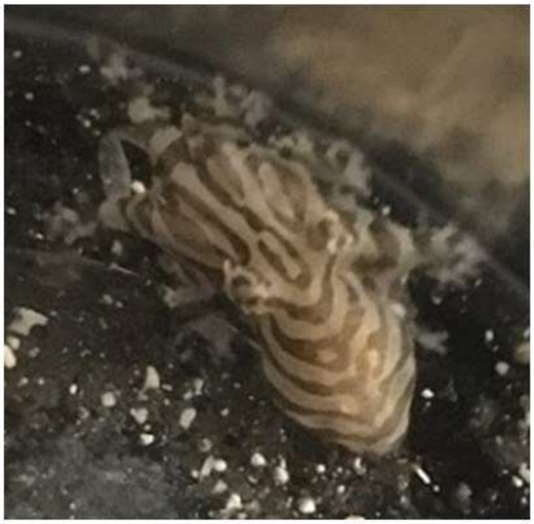

17.

Mark only one oval.

- match
- no match

18

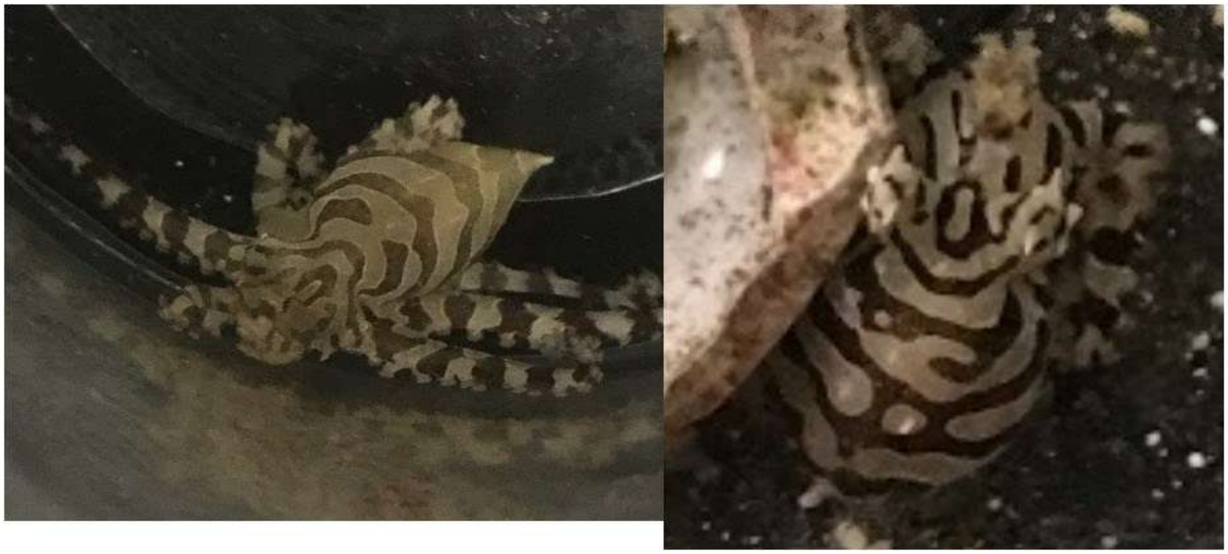

18.

Mark only one oval.

- match
- no match

19

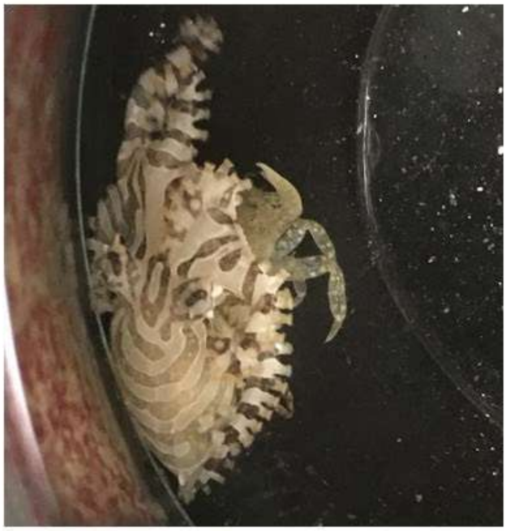

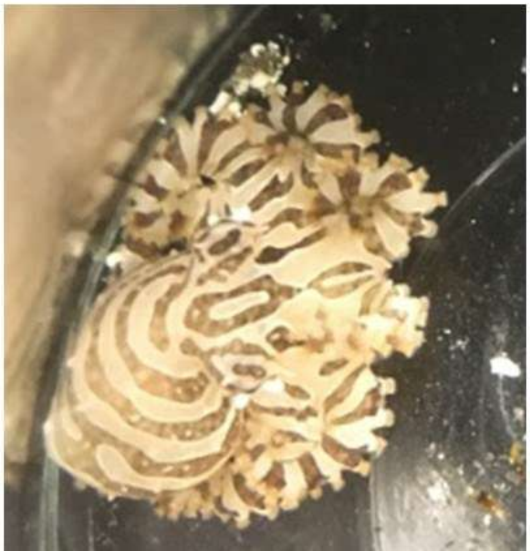

19.

Mark only one oval.

- match
- no match

20

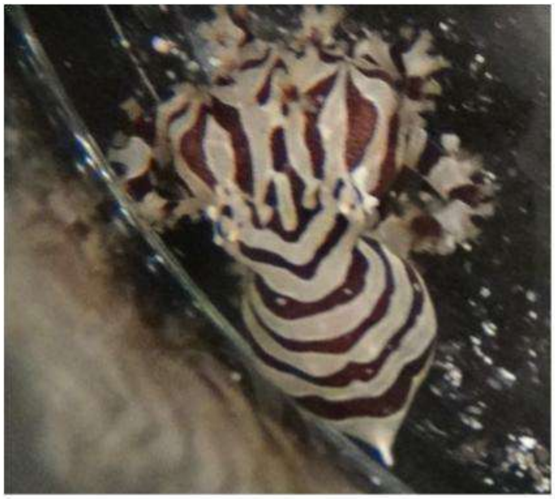

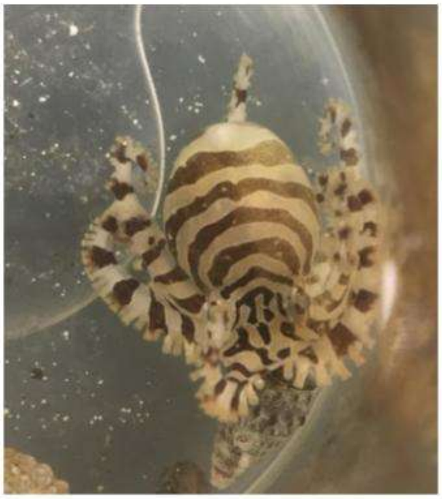

20.

Mark only one oval.

- match
- no match

21

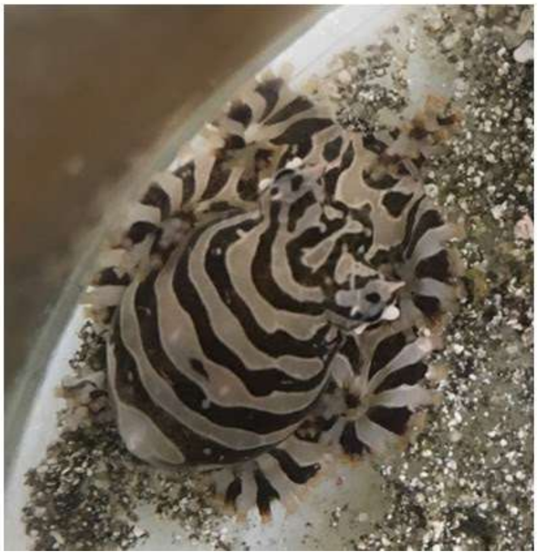

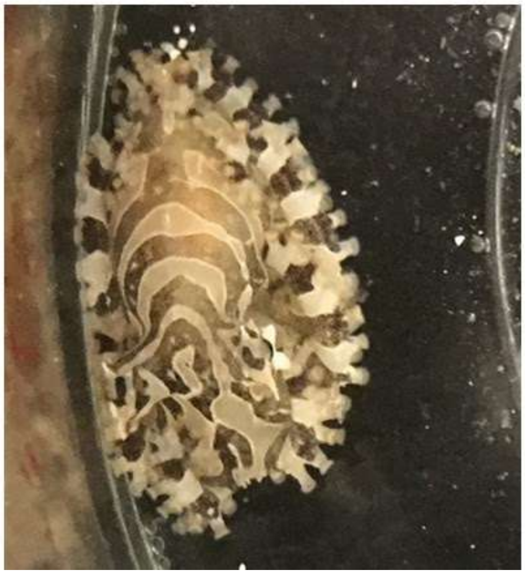

21.

Mark only one oval.

- match
- no match

22

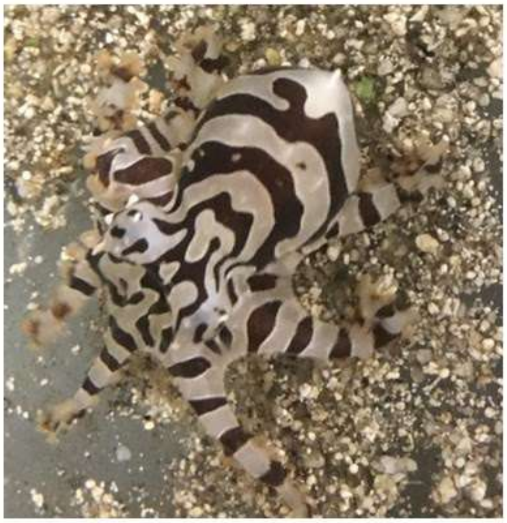

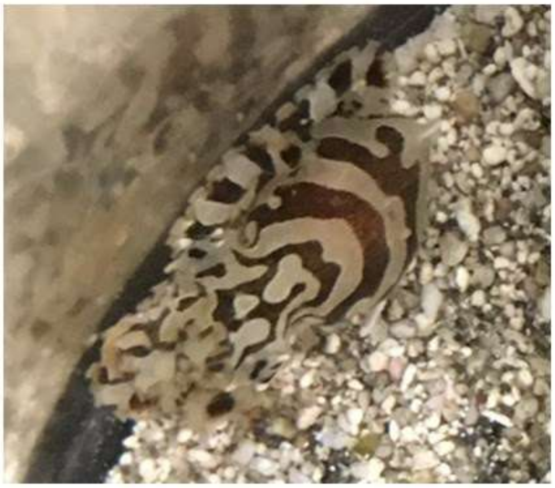

22.

Mark only one oval.

- match
- no match

23

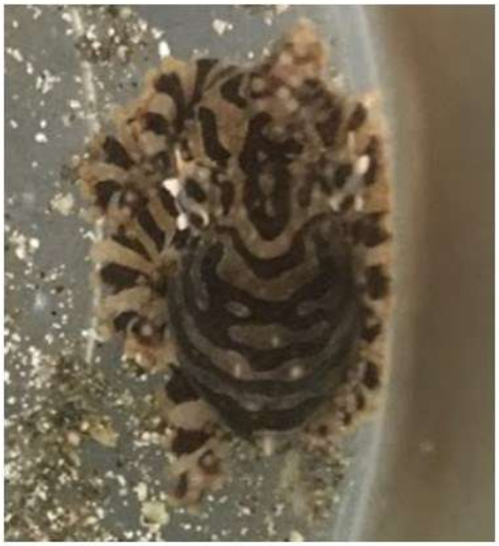

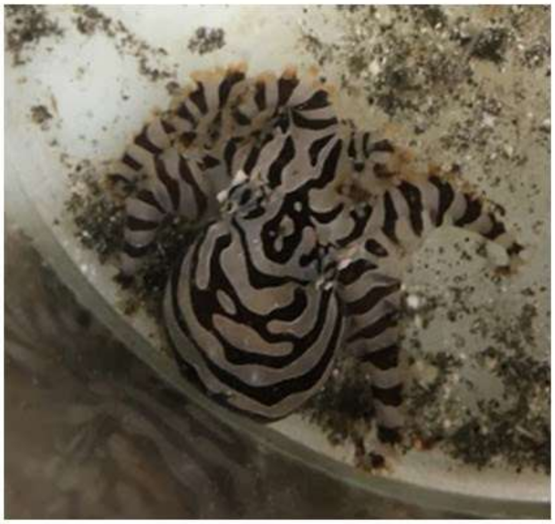

23.

Mark only one oval.

- match
- no match

24

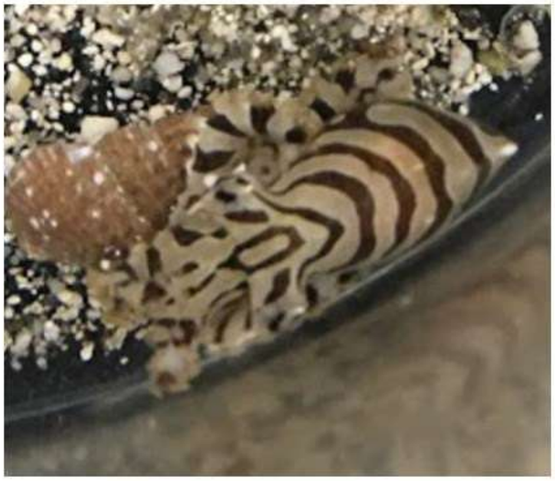

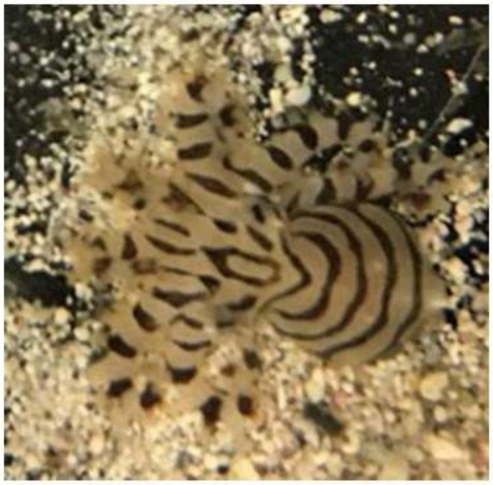

24.

Mark only one oval.

- match
- no match

25

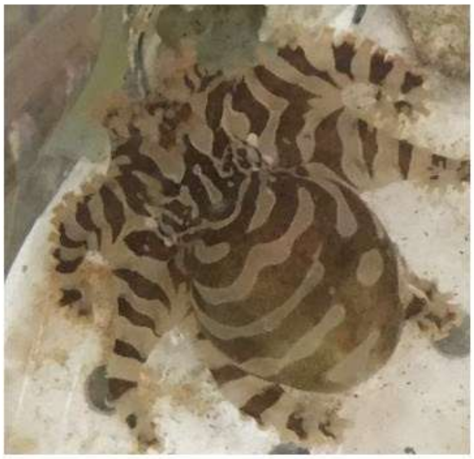

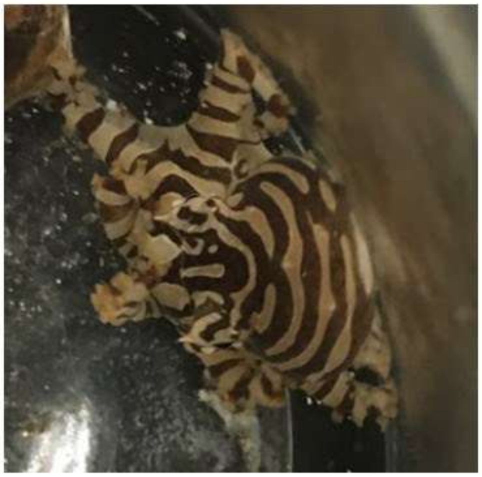

25.

Mark only one oval.

- match
- no match

26

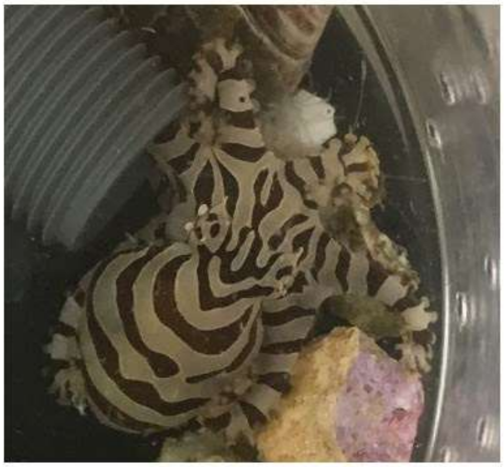

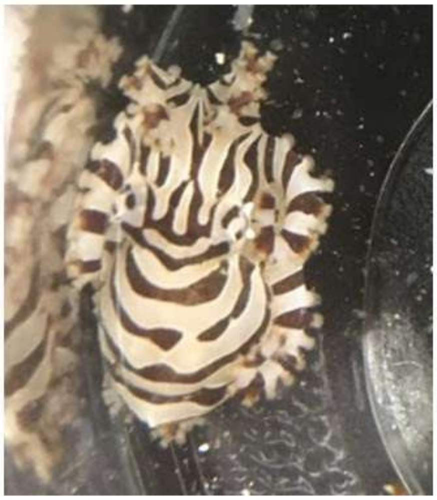

26.

Mark only one oval.

- match
- no match

27

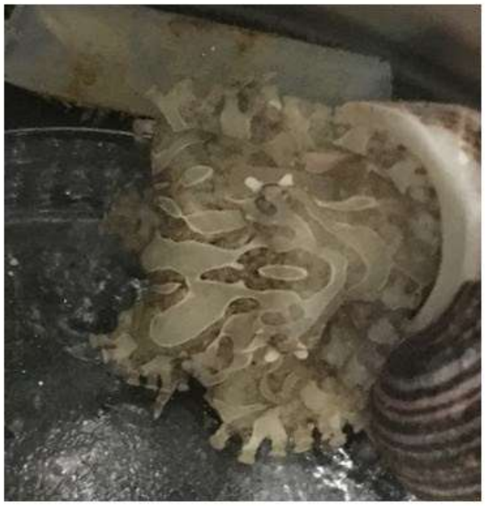

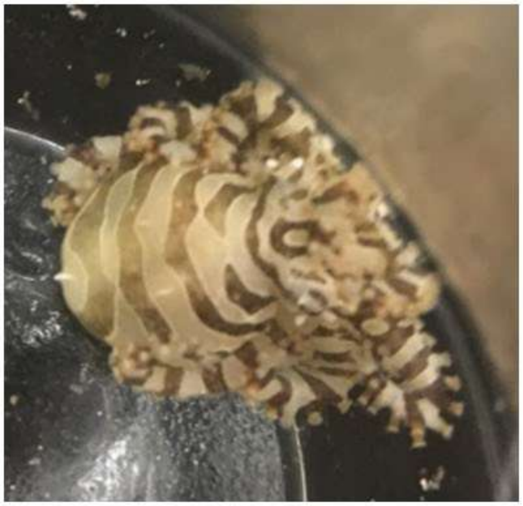

27.

Mark only one oval.

- match
- no match

28

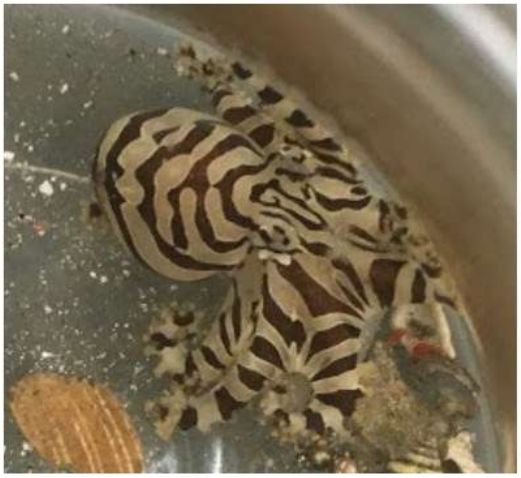

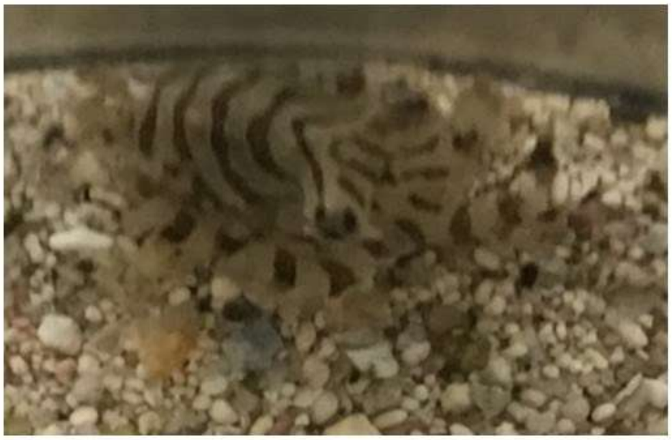

28.

Mark only one oval.

- ( ) match
- ( ) no match

29

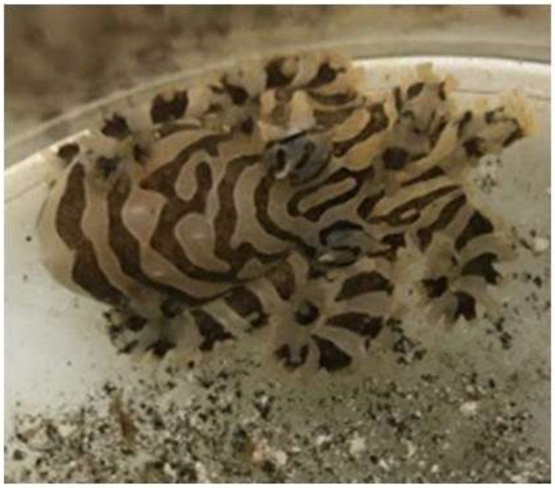

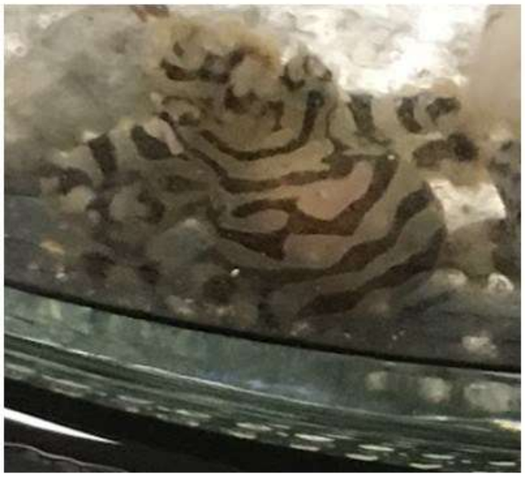

29.

Mark only one oval.

- match
- no match

30

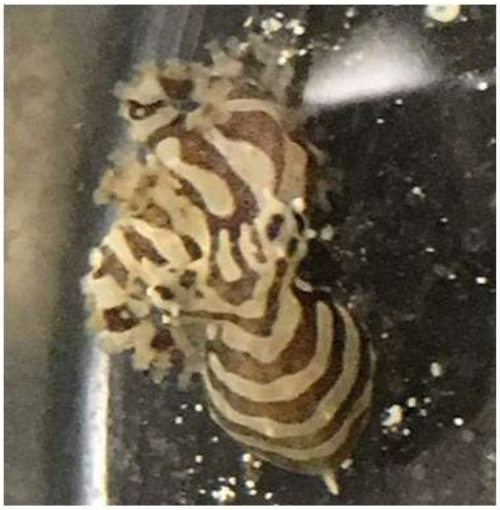

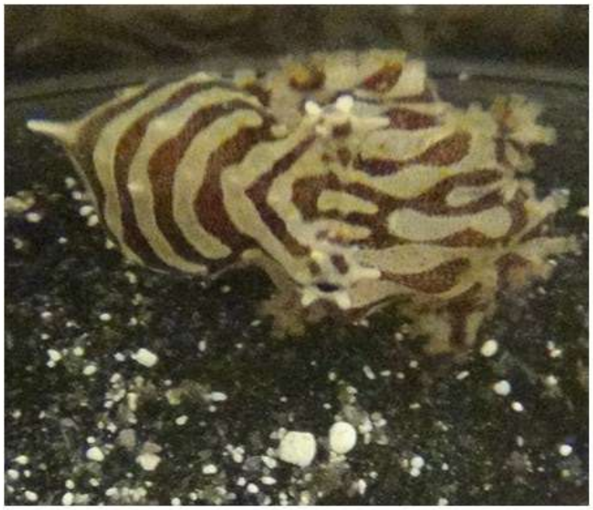

30.

Mark only one oval.

- match
- no match

31

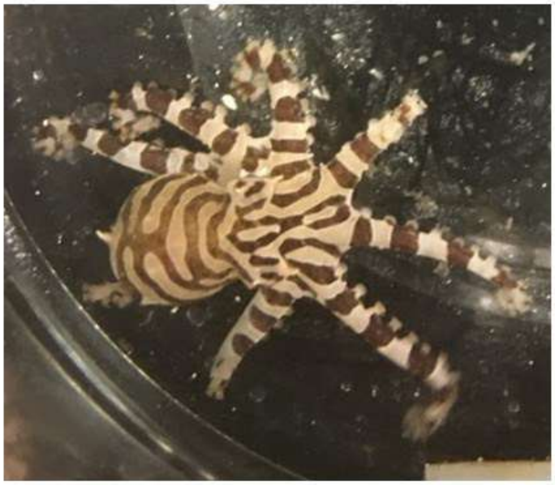 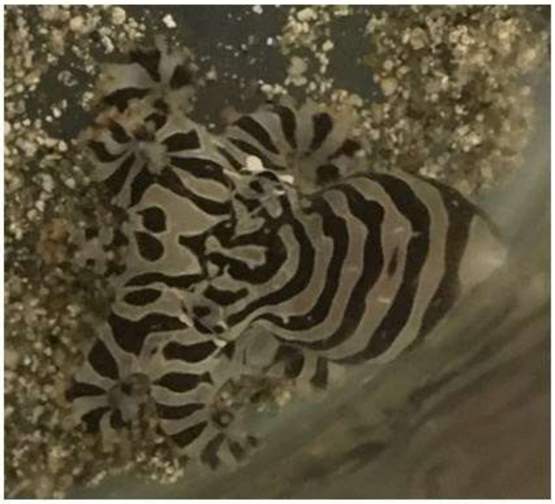

31.
Mark only one oval.

- match
- no match

32

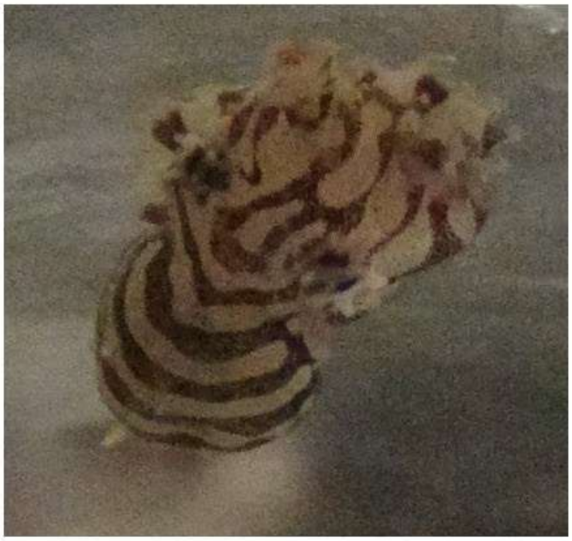

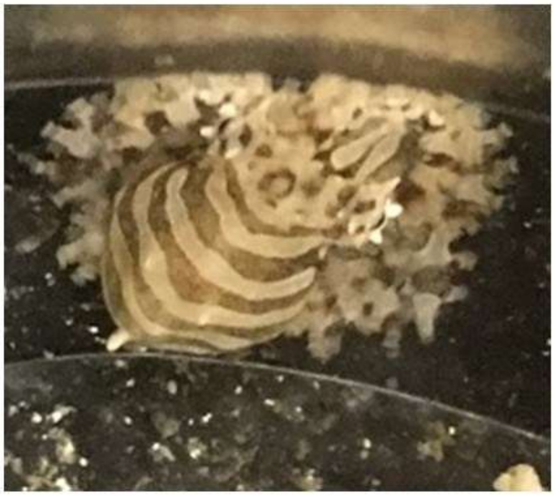

32.
Mark only one oval.

- match
- no match

33

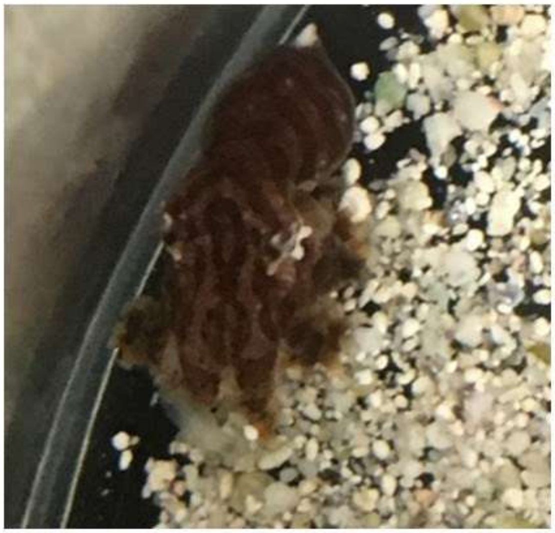

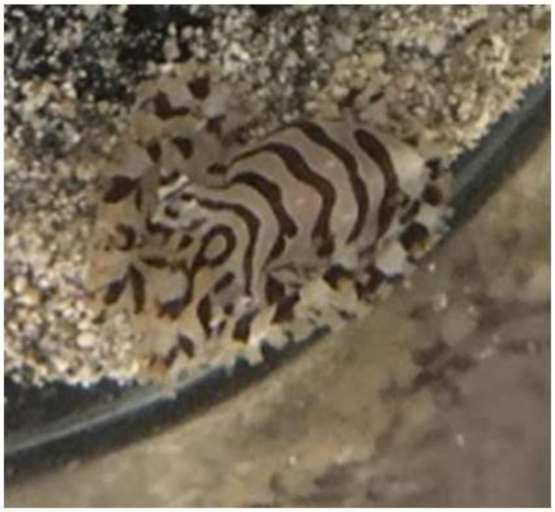

33.

Mark only one oval.

- match
- no match

34

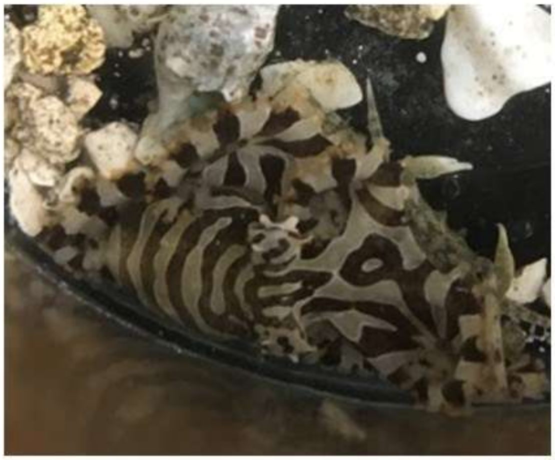

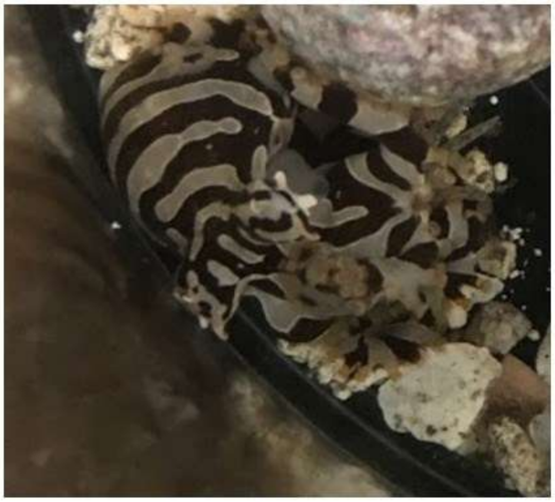

34.

Mark only one oval.

- match
- no match

35

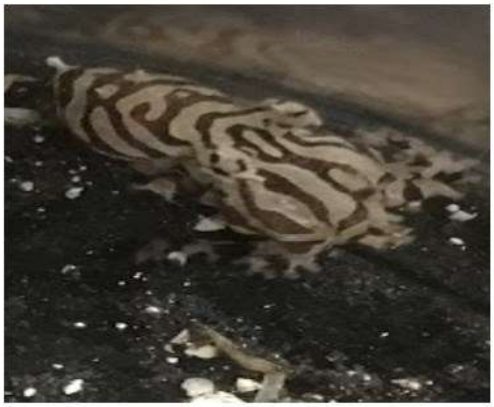

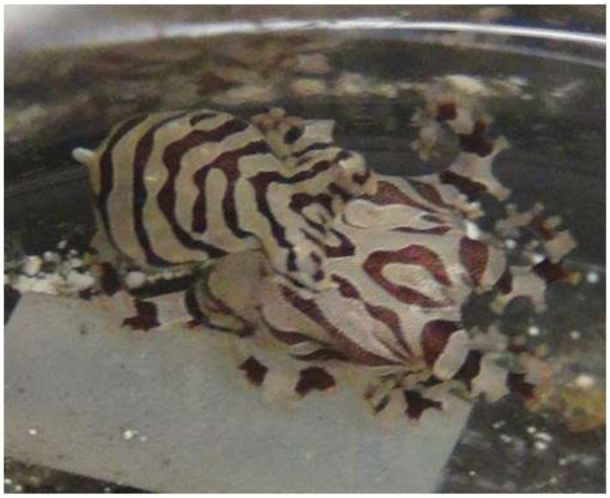

35.
Mark only one oval.

- ( ) match
- ( ) no match

36

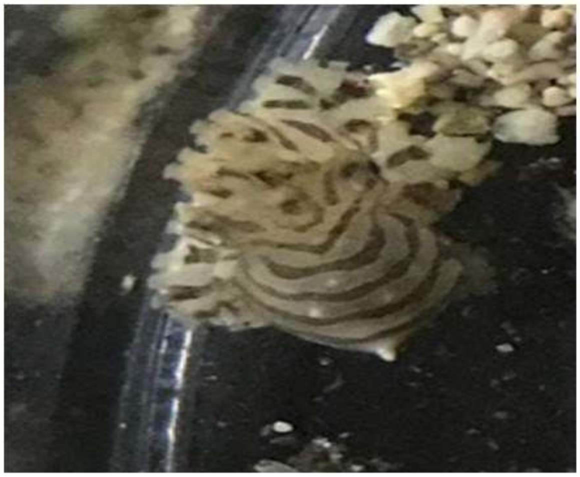

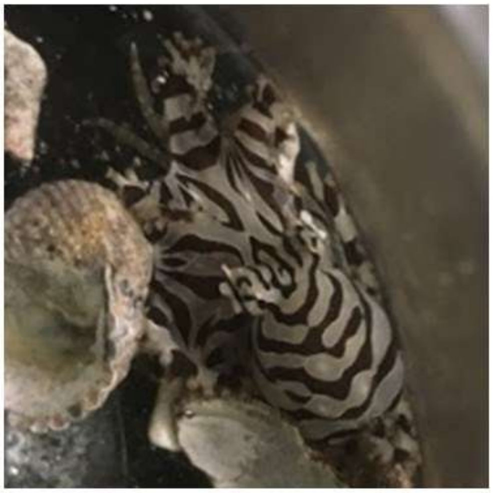

36. *Mark only one oval.*

- match
- no match

37

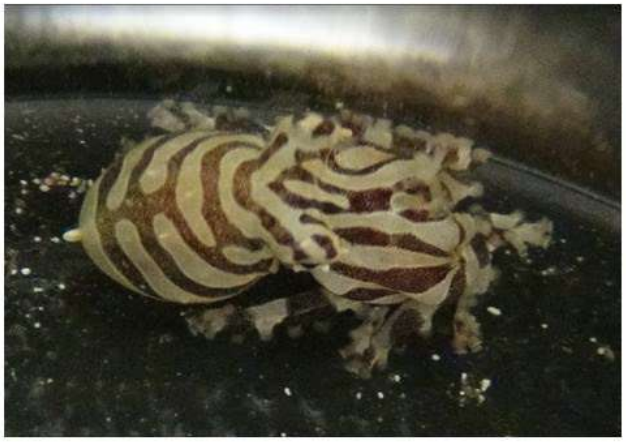

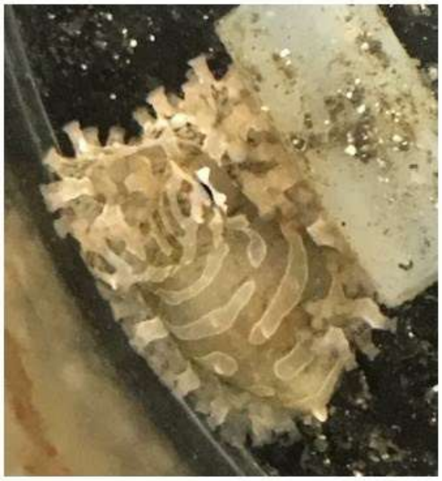

37.

Mark only one oval.

- match
- no match

38

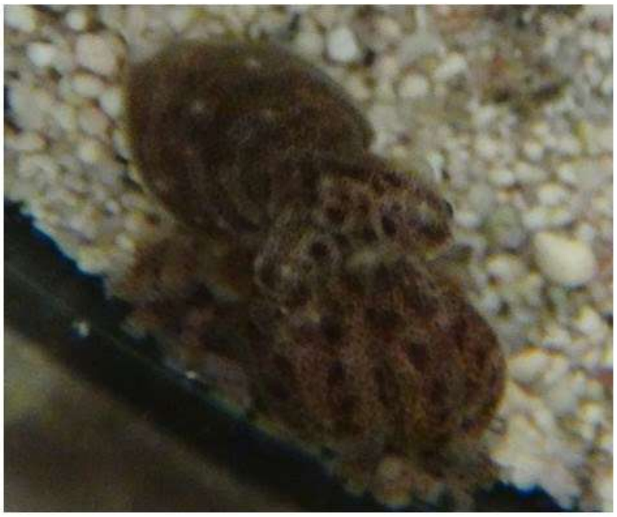

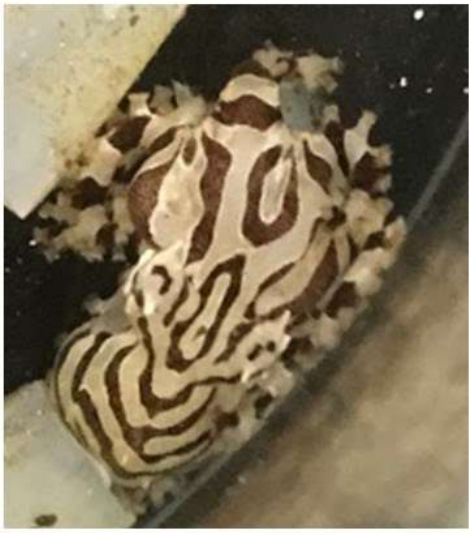

38.

Mark only one oval.

- match
- no match

39

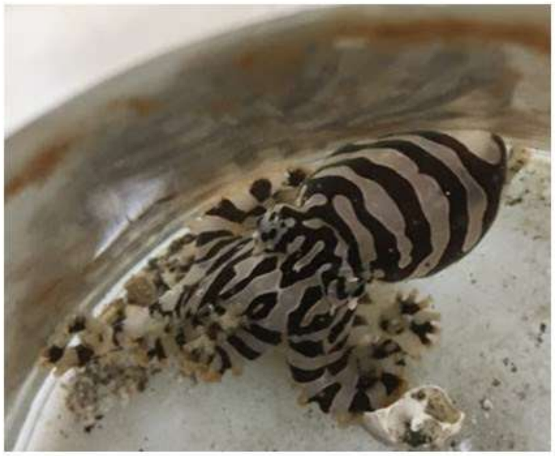

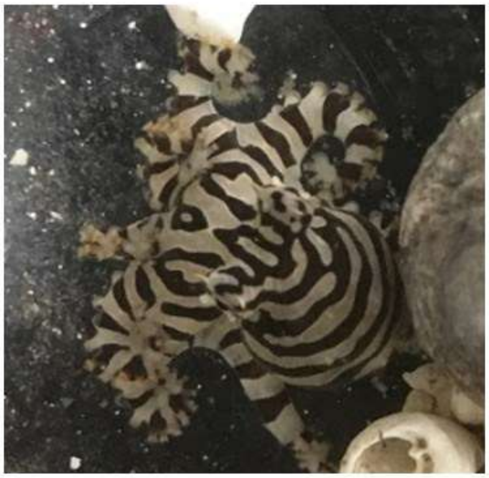

39.

Mark only one oval.

- match
- no match

40

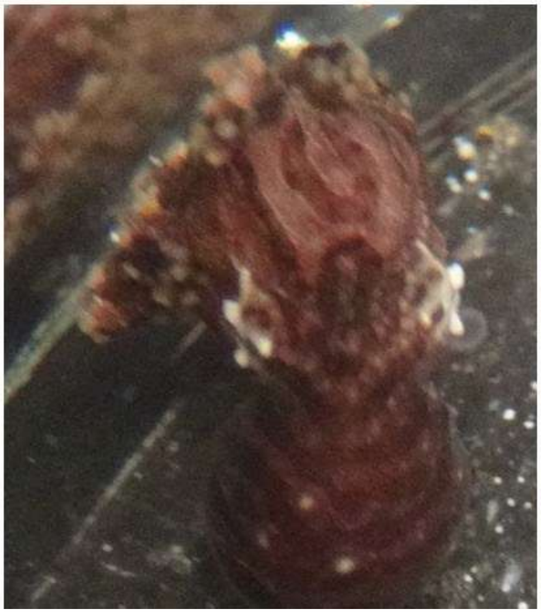

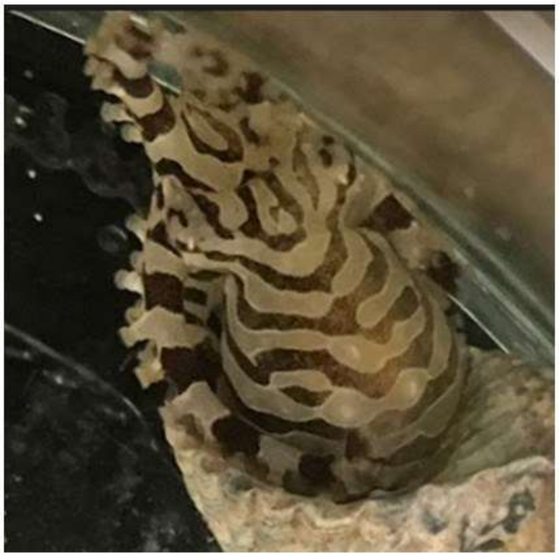

40.

Mark only one oval.

- match
- no match

41

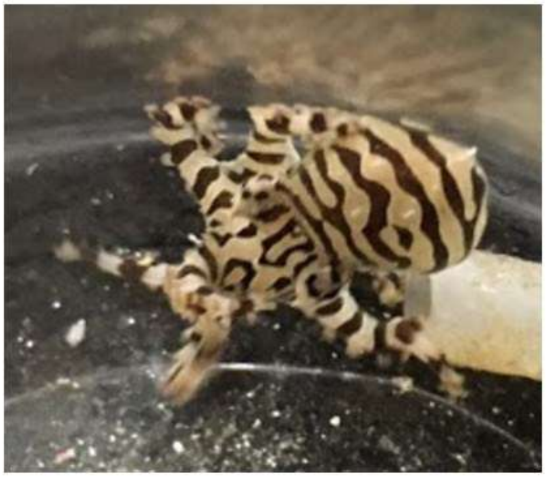

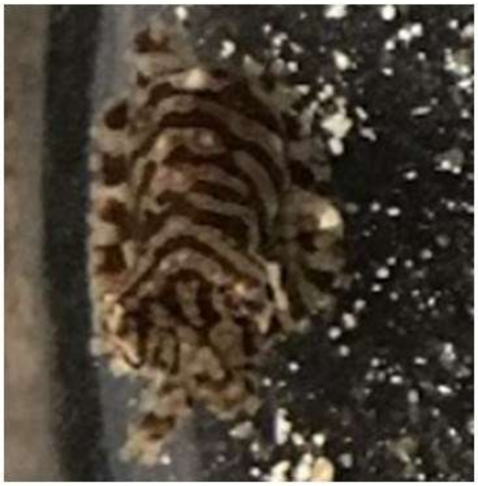

41.

Mark only one oval.

- match
- no match

42

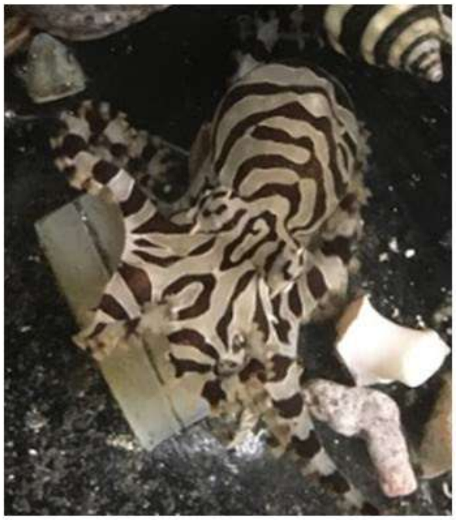

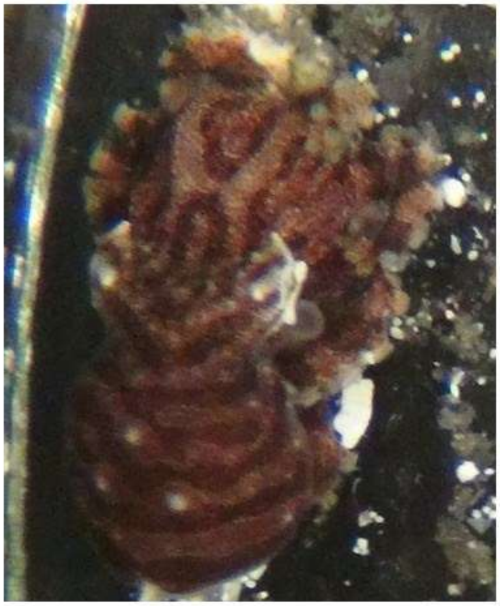

42.

Mark only one oval.

- match
- no match

43

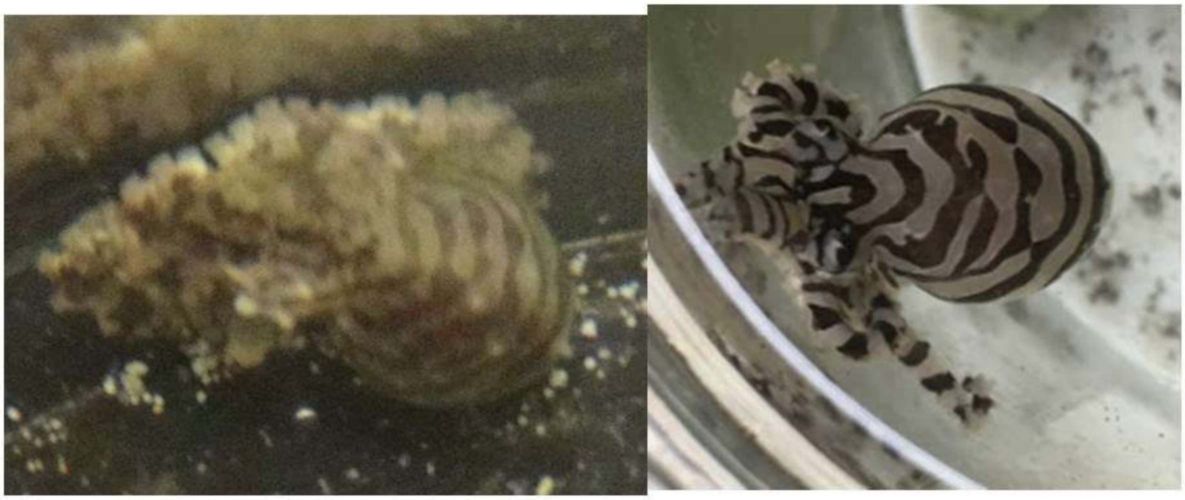

43.

Mark only one oval.

- match
- no match

44

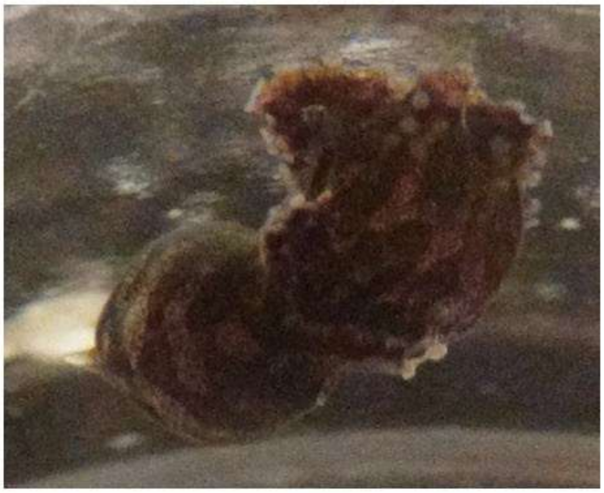

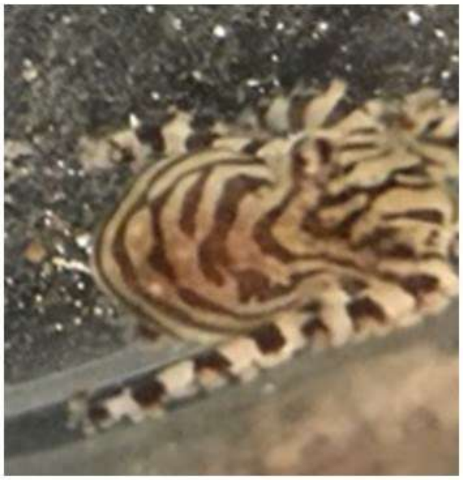

44.

Mark only one oval.

- match
- no match

45

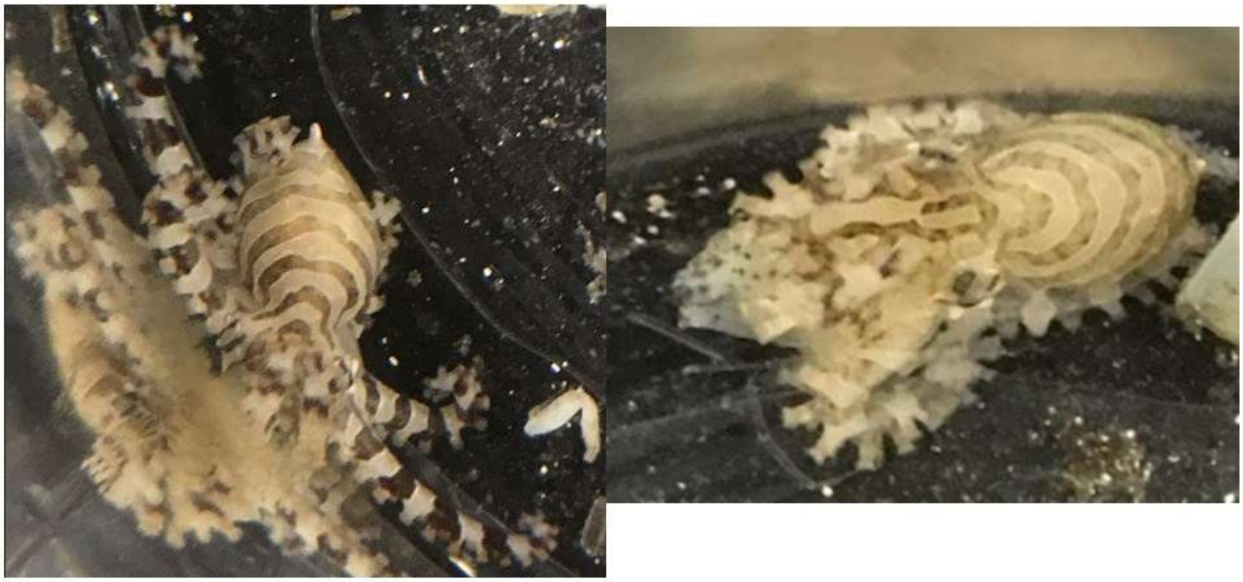

45\.

Mark only one oval.

- ⬭ match
- ⬭ no match

46

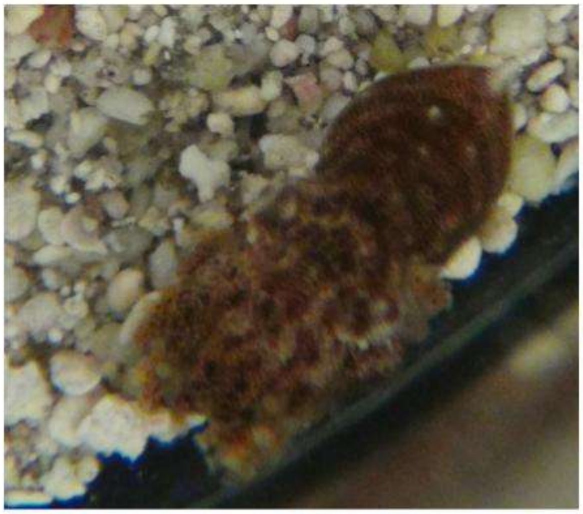

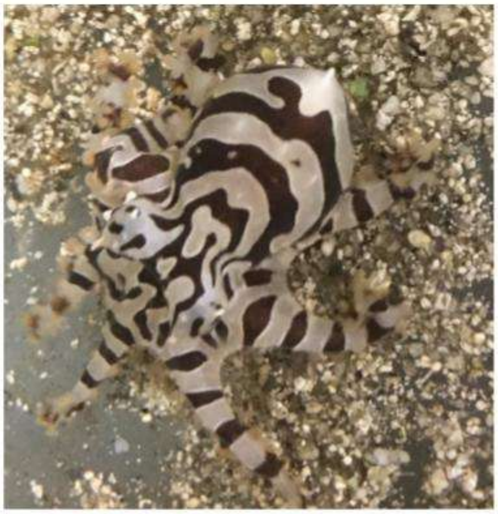

46. *Mark only one oval.*

- match
- no match

47

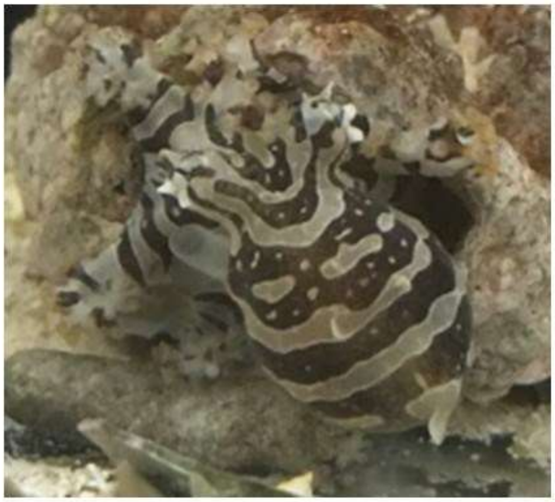

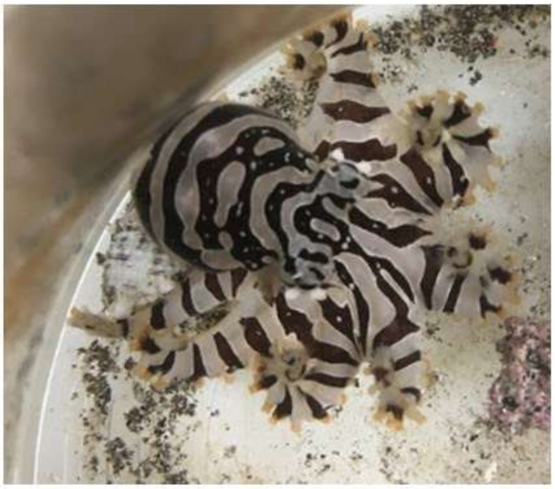

47.

Mark only one oval.

- match
- no match

48

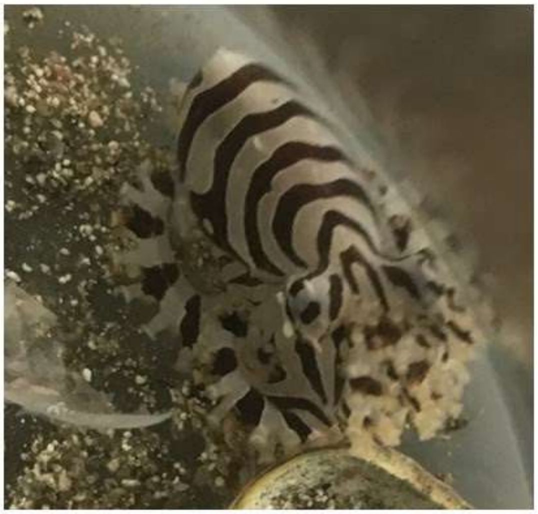

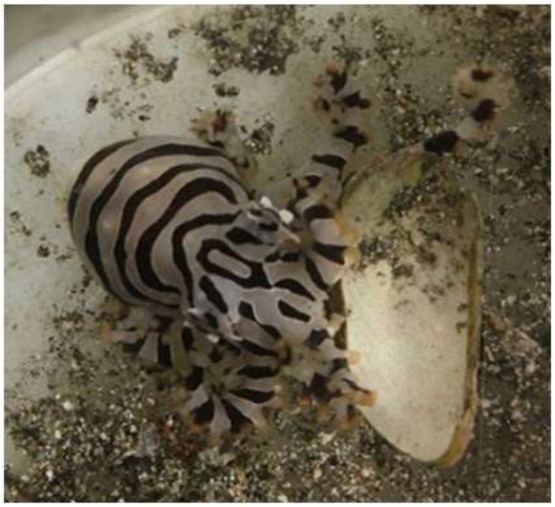

48.

Mark only one oval.

- match
- no match

49

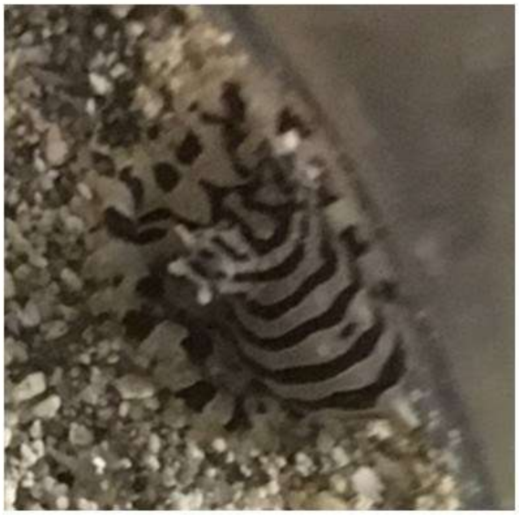

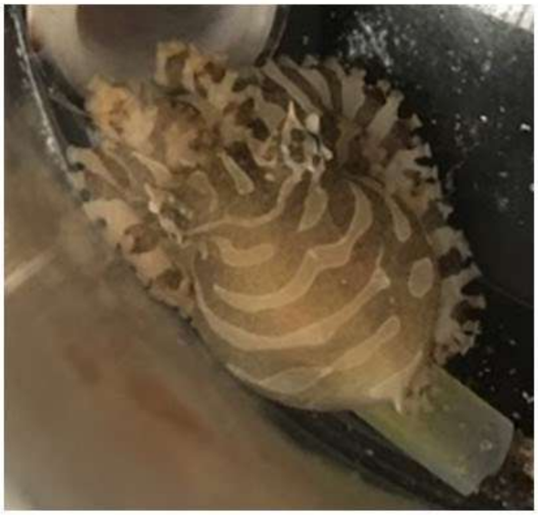

49.

Mark only one oval.

- match
- no match

50

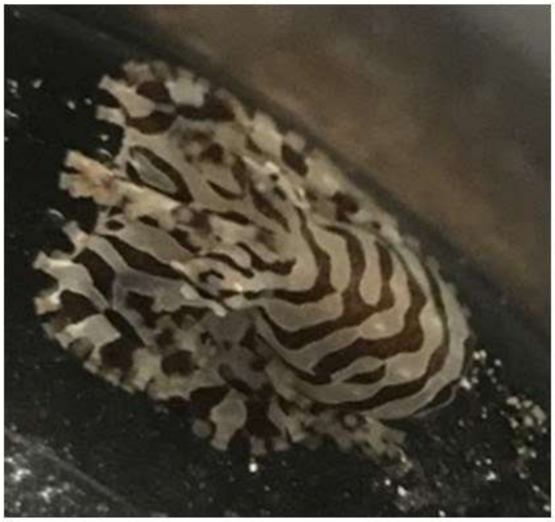

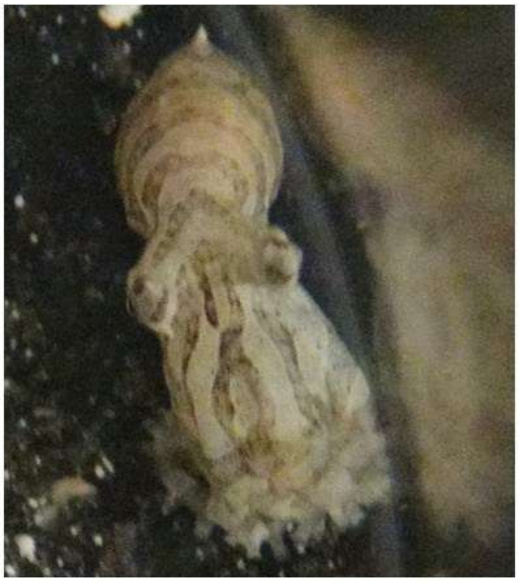

50.

Mark only one oval.

- match
- no match

This content is neither created nor endorsed by Google.

Google Forms

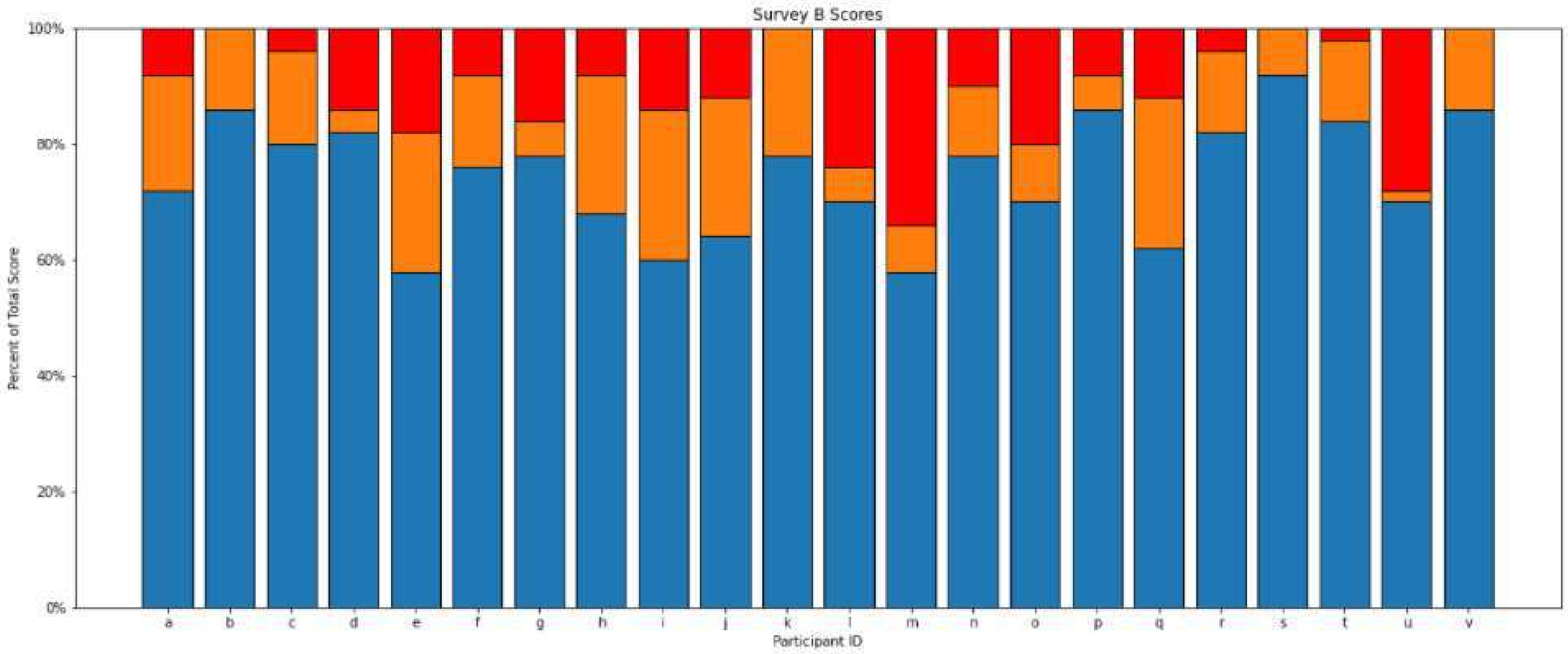
Survey B Scores
100%
80%
60%
40%
20%
0%
Percent of Total Score
a
b
c
d
e
f
g
h
i
j
k
l
m
n
o
p
q
r
s
t
u
v
Participant ID

Octopus Chierchiae Photo identification Survey B

Each of the following questions displays 2 photos (pattern traces provided directly below each photo for assistance).

Please mark "Match" if you think the photos are of the same individual or "no match" if you think they are two different animals.

(Note: the images may be taken from different angles and the animals can distort their bodies and colors)

1

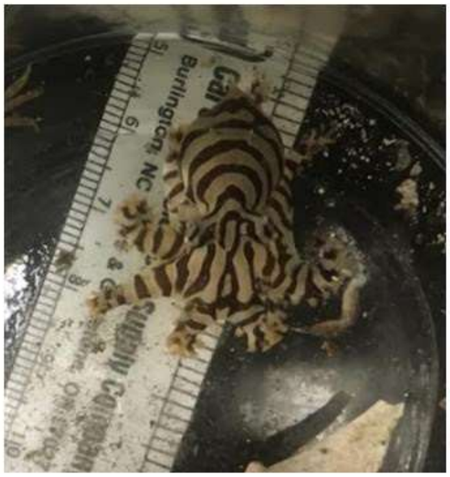

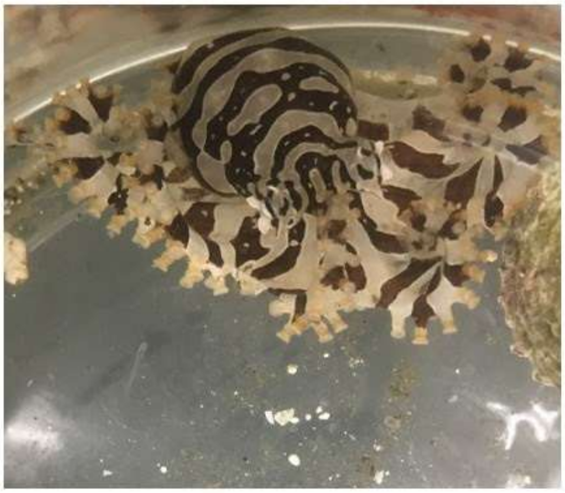

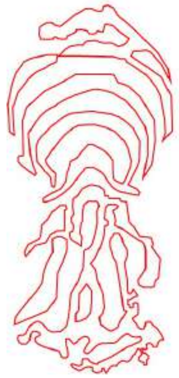

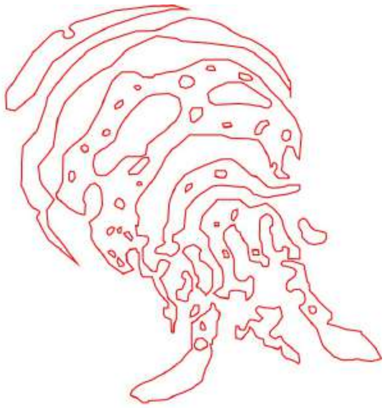

1. *Mark only one oval.*

- ( ) match
- ( ) no match

2

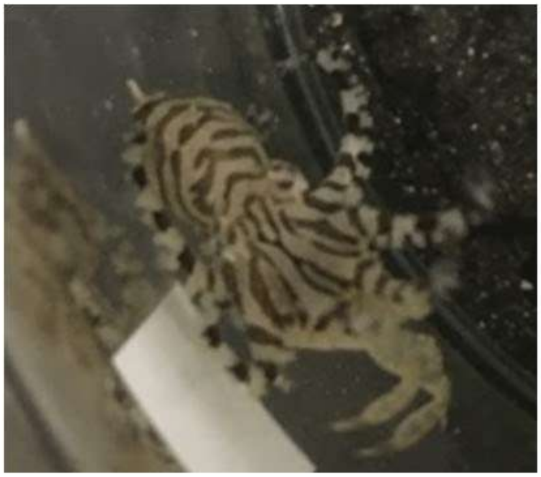

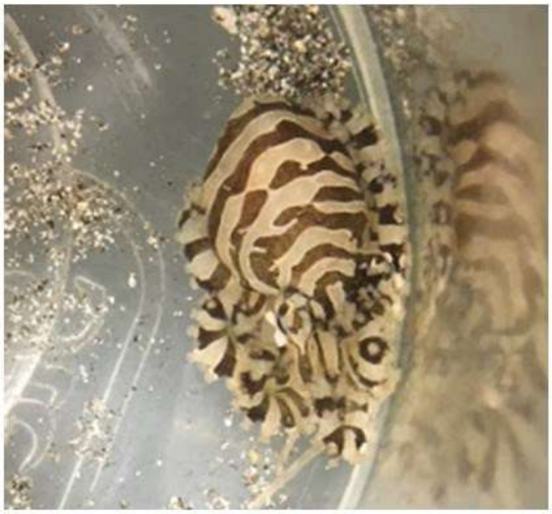

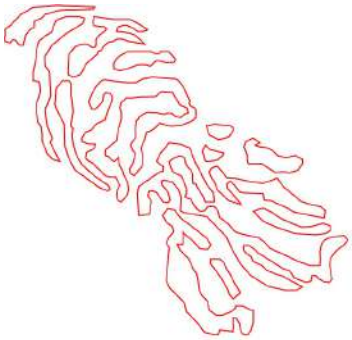

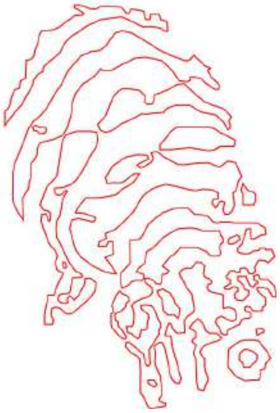

2.
Mark only one oval.

- match
- no match

3

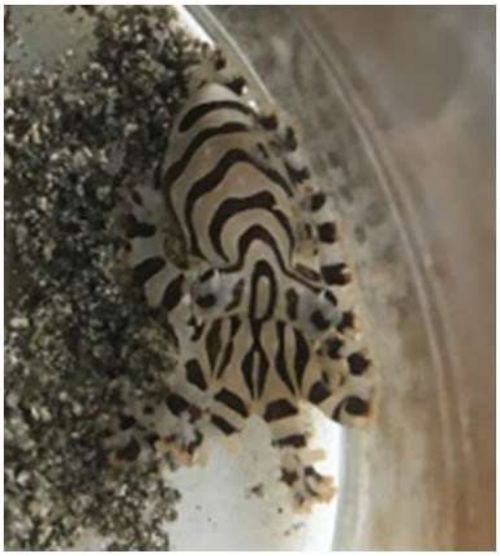

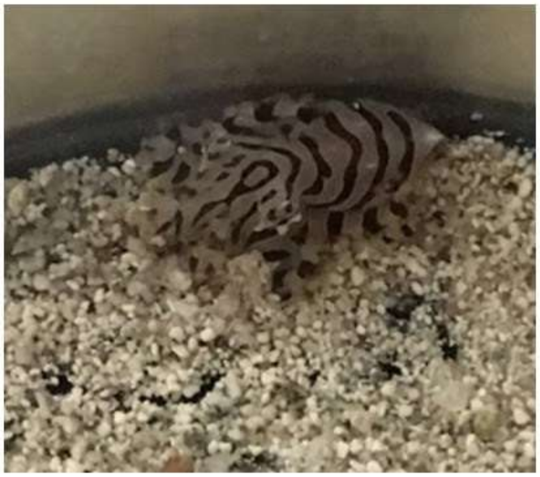

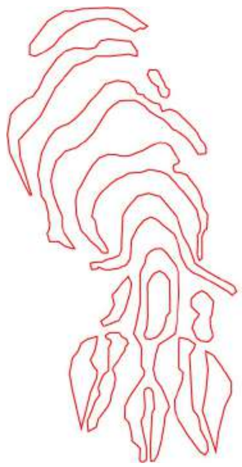

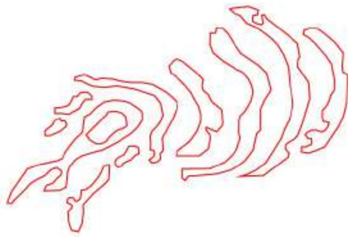

3.
Mark only one oval.

- match
- no match

4

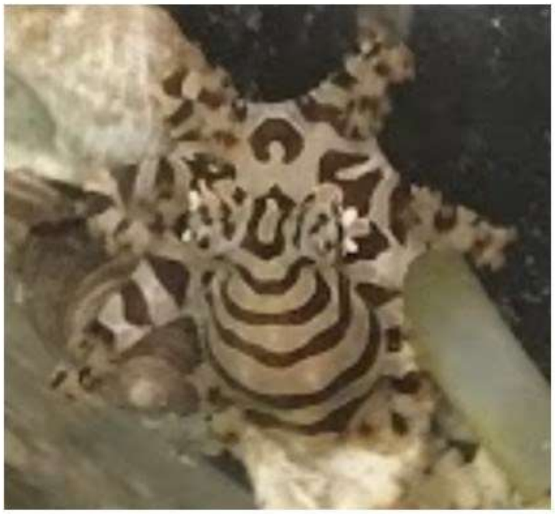

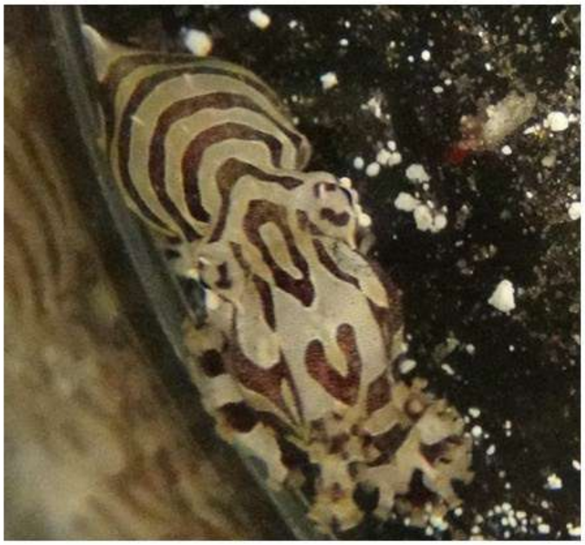

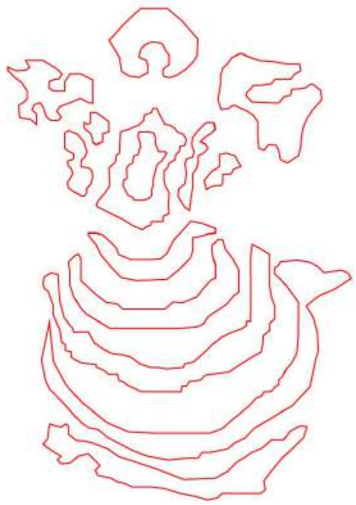

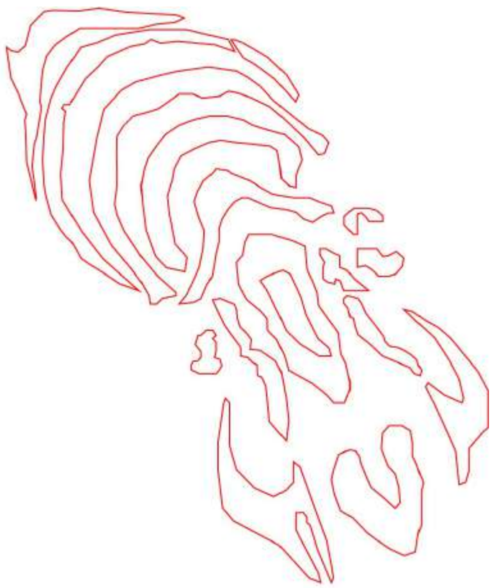

4.
Mark only one oval.

- match
- no match

5

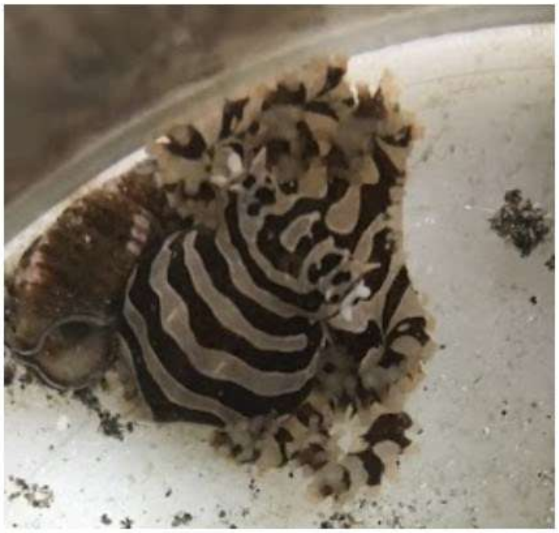

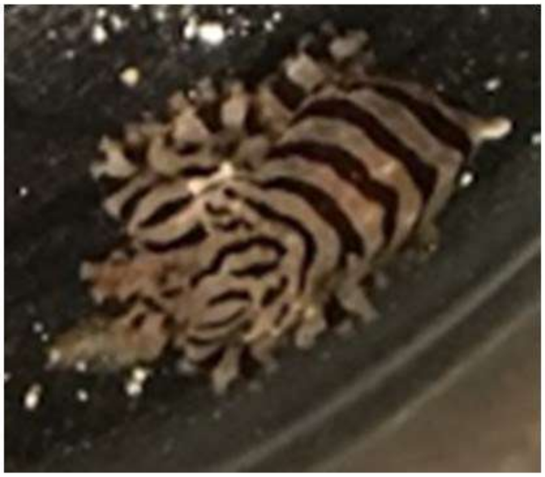

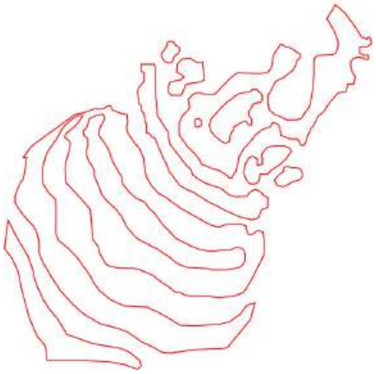

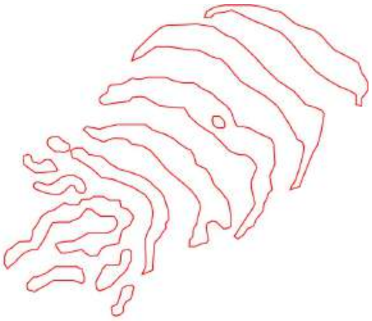

5.
Mark only one oval.

- match
- no match

6

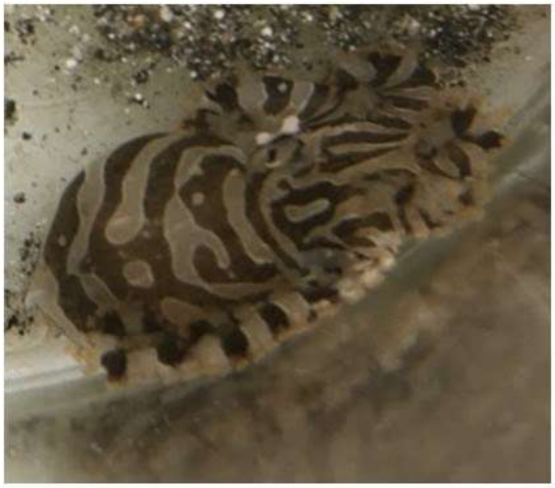

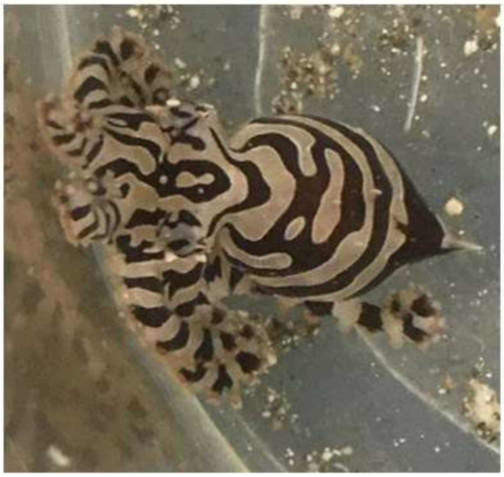

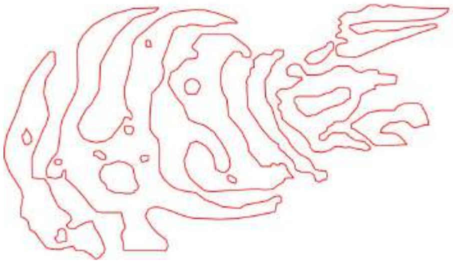

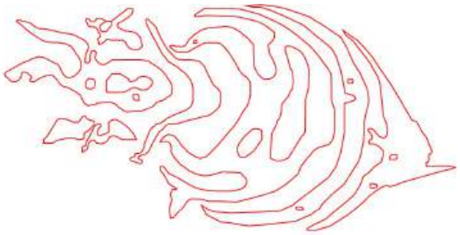

6.

Mark only one oval.

- match
- no match

7

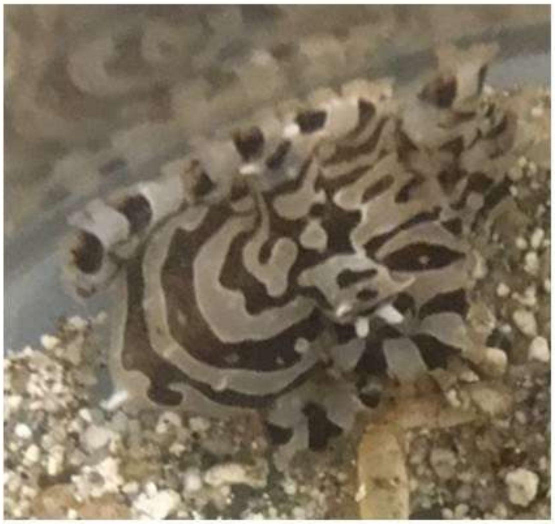

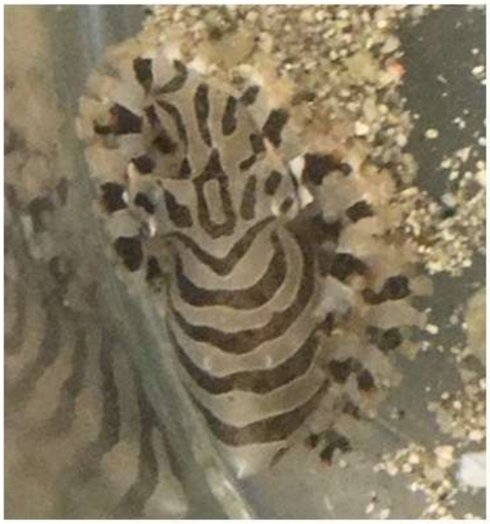

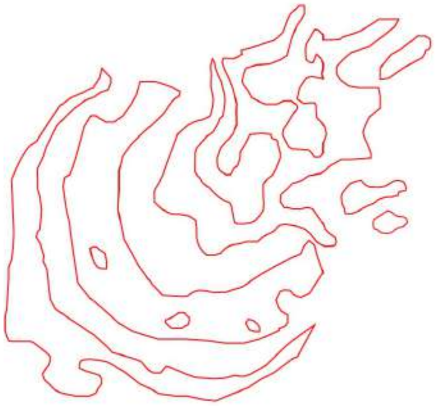

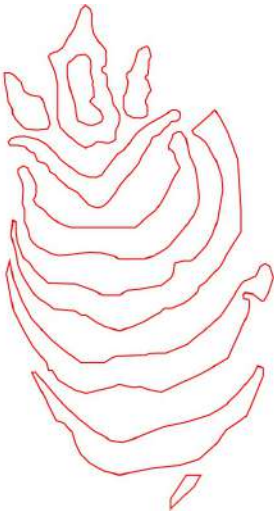

7\. *Mark only one oval.*

- ◯ match
- ◯ no match

8

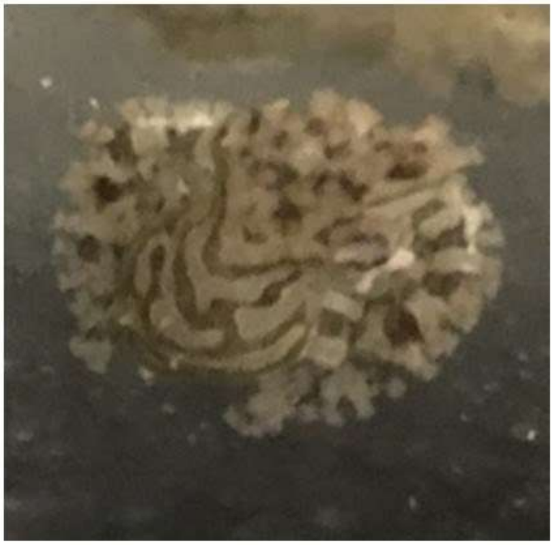

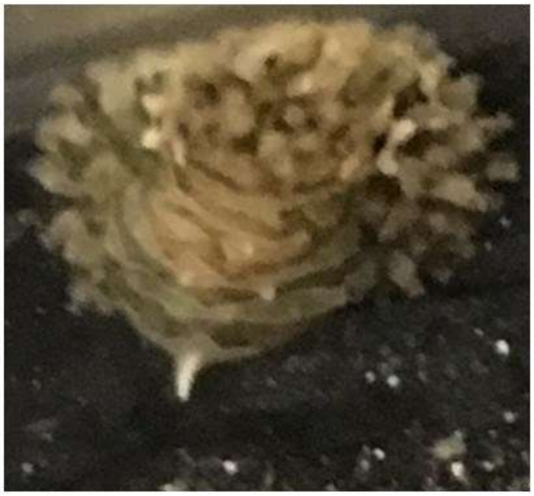

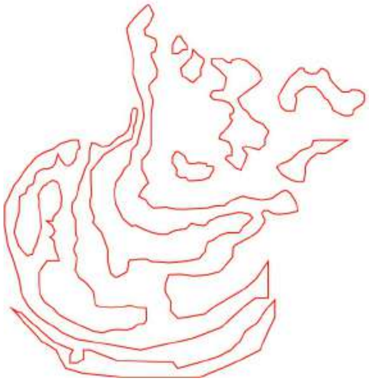

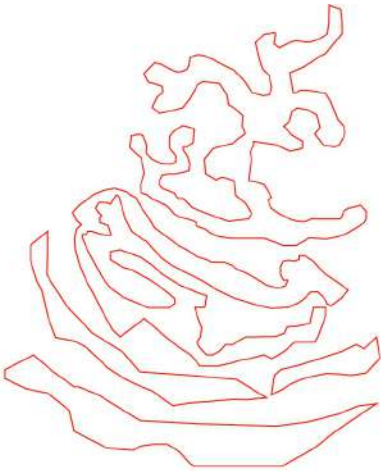

8\.

Mark only one oval.

- match
- no match

9

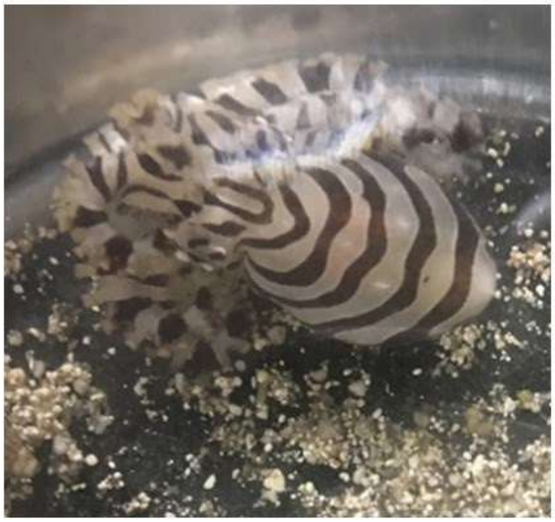

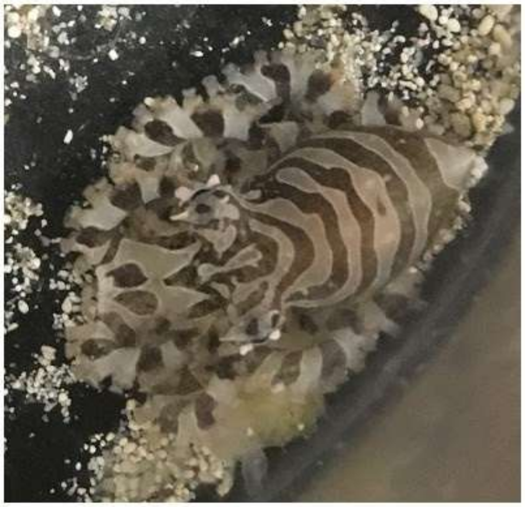

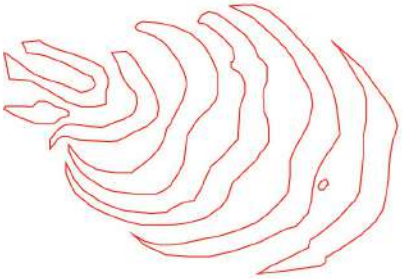

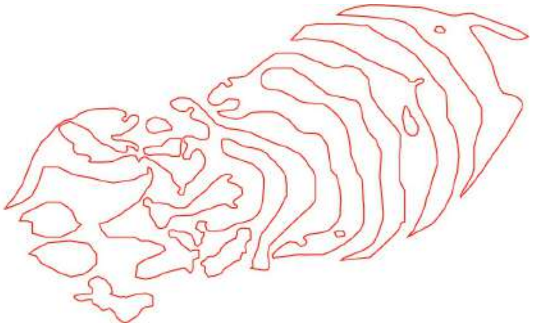

9.

Mark only one oval.

- ◯ match
- ◯ no match

10

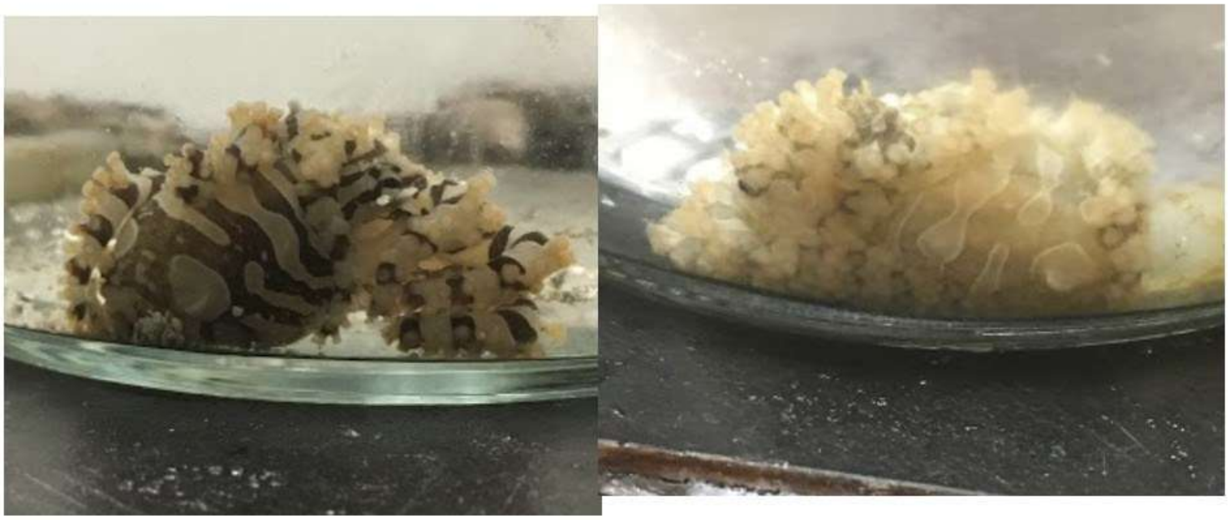

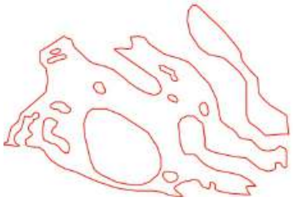

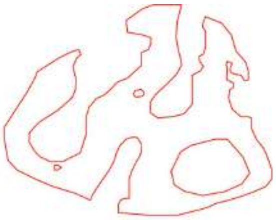

10.

Mark only one oval.

- ( ) match
- ( ) no match

11

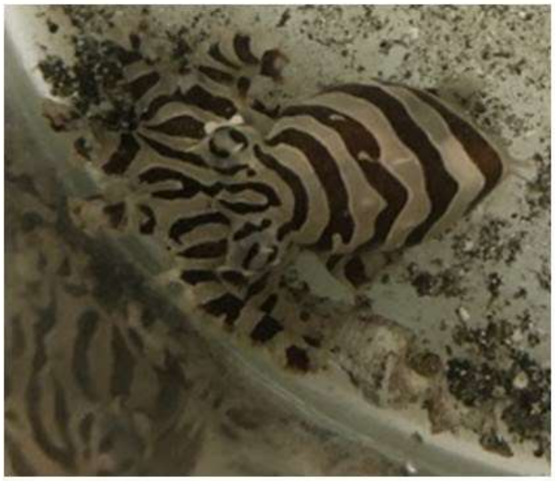

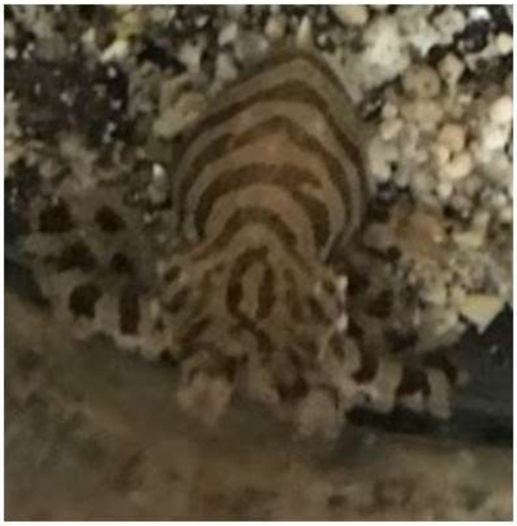

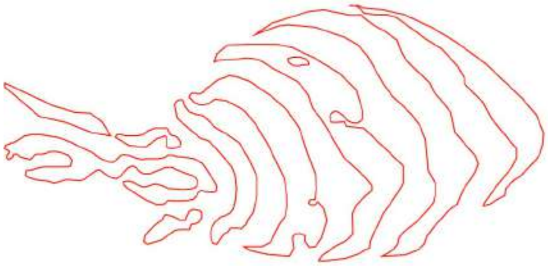

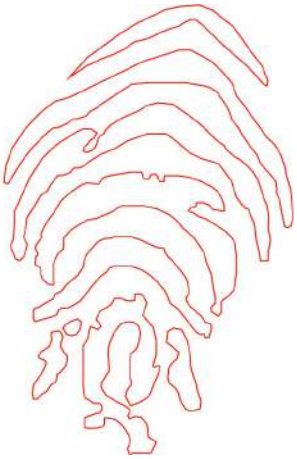

11.

Mark only one oval.

- match
- no match

12

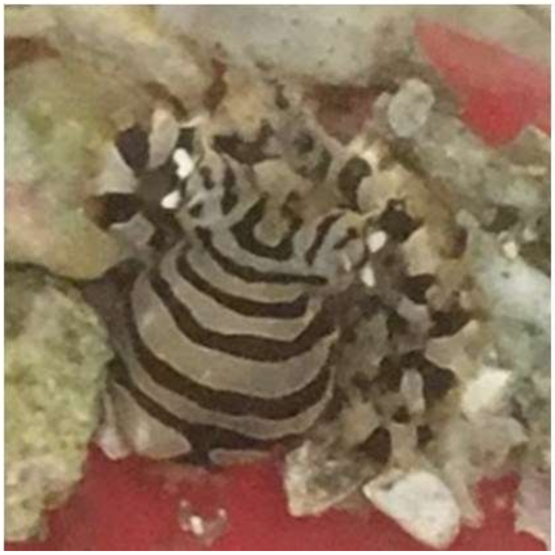

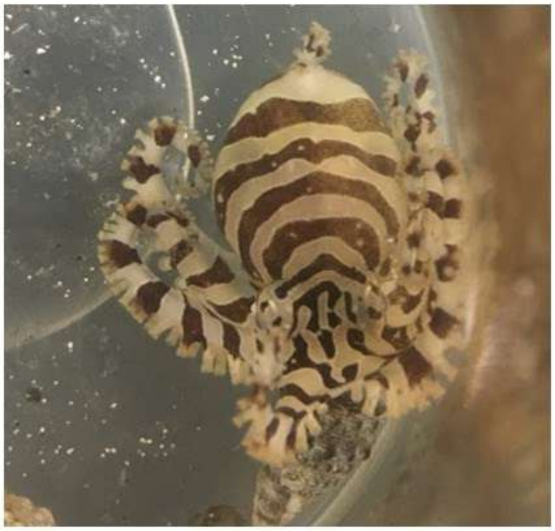

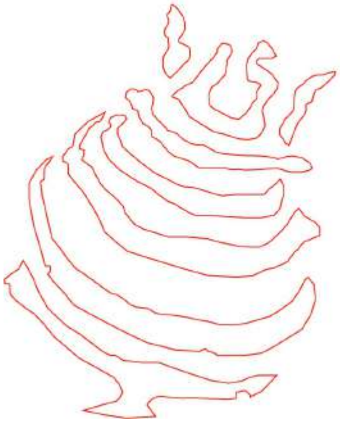

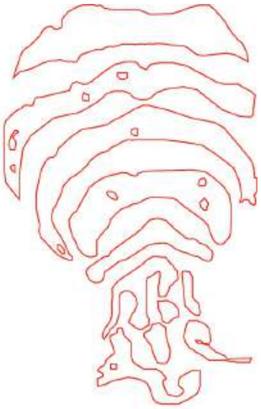

12.

Mark only one oval.

- match
- no match

13

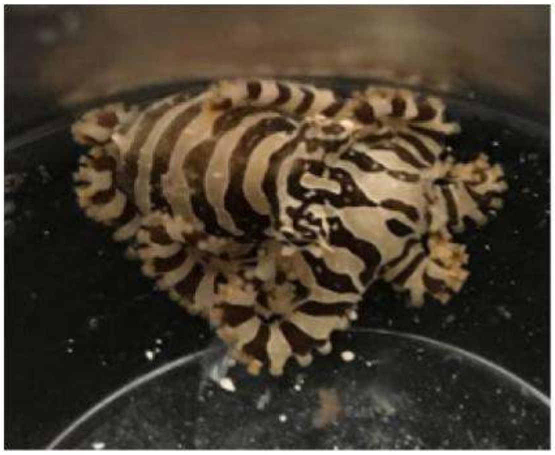

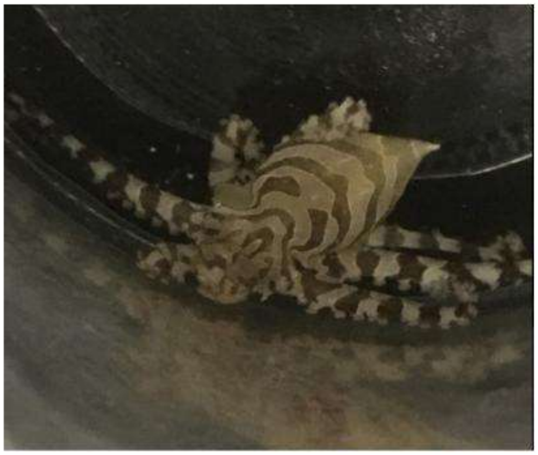

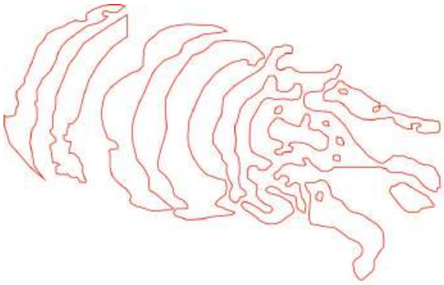

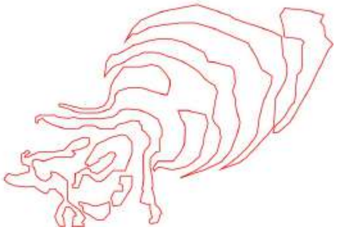

13.

Mark only one oval.

- match
- no match

14

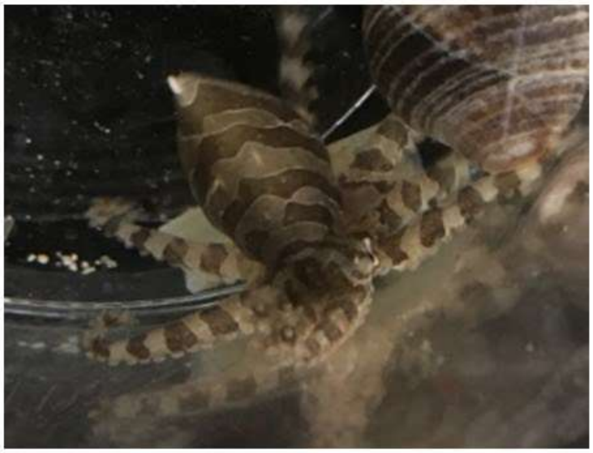

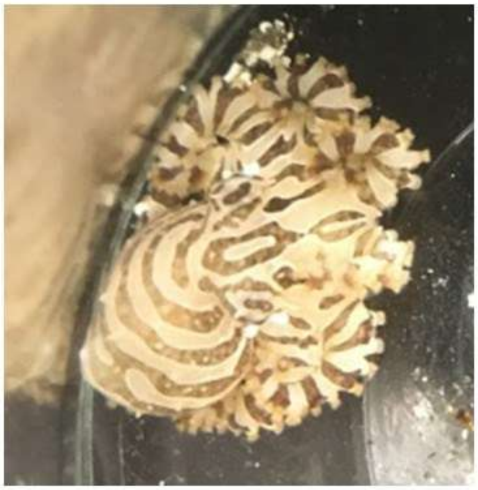

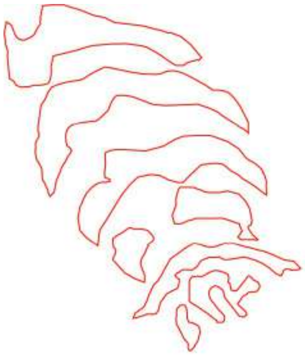

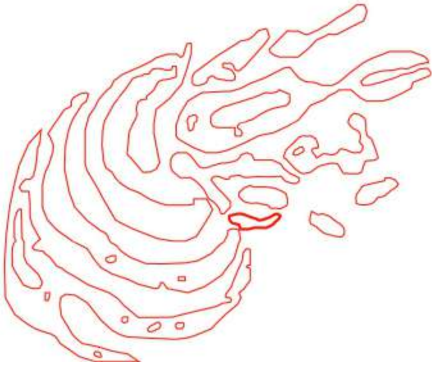

14.

Mark only one oval.

- match
- no match

15

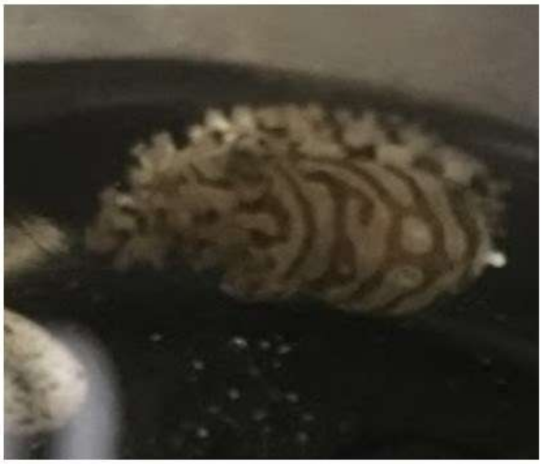

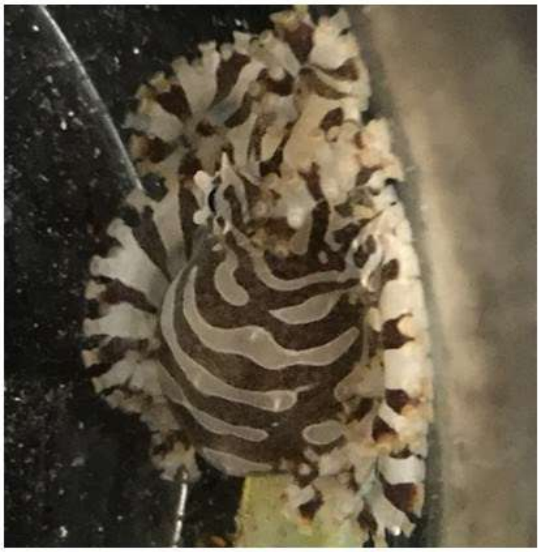

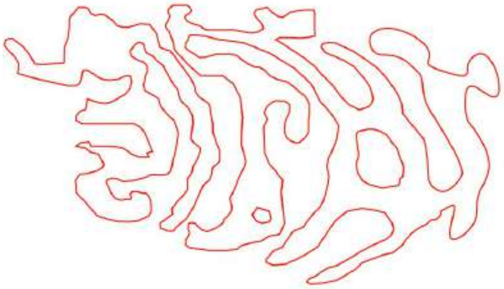

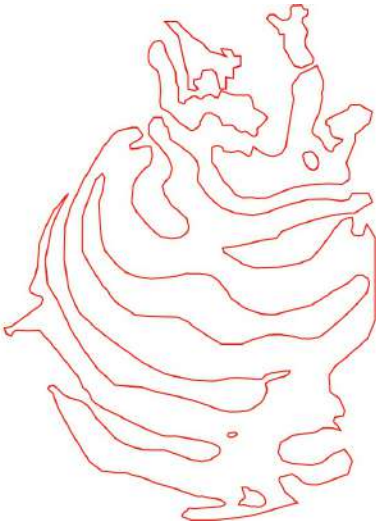

15.
Mark only one oval.

- match
- no match

16

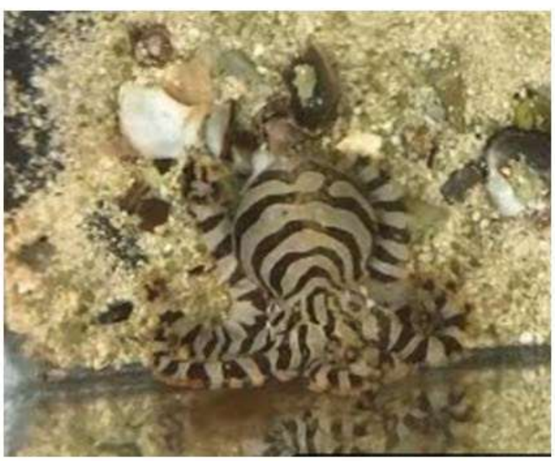
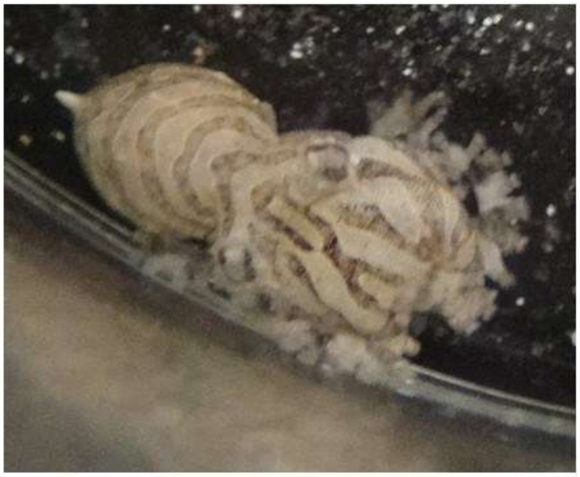

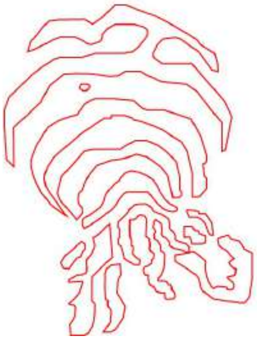
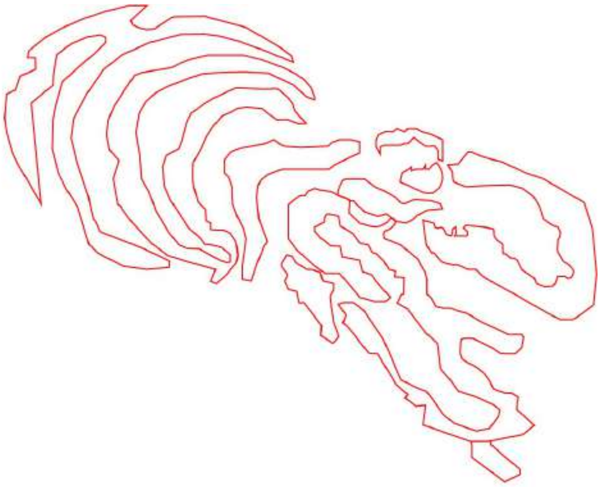

16.

Mark only one oval.

- ⬭ match
- ⬭ no match

17

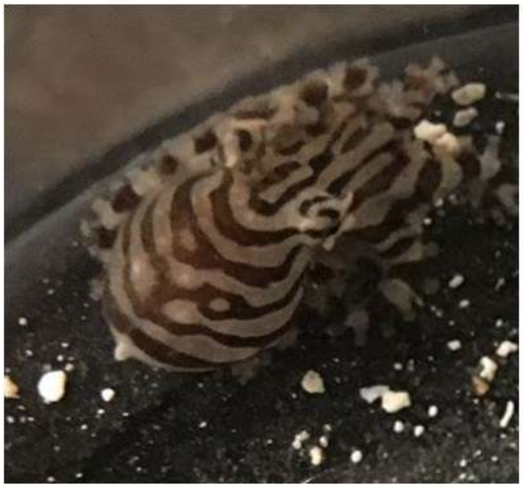

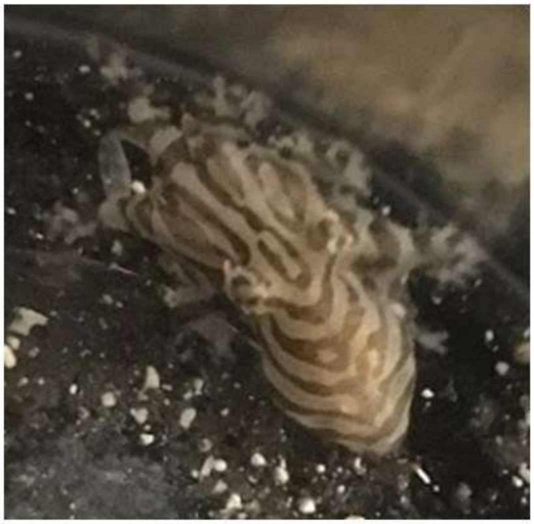

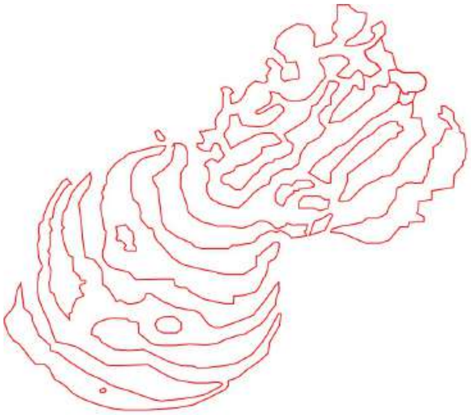

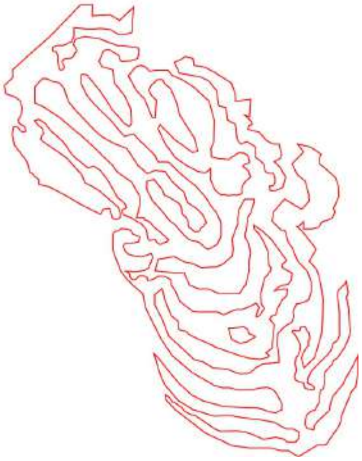

17.

Mark only one oval.

- match
- no match

18

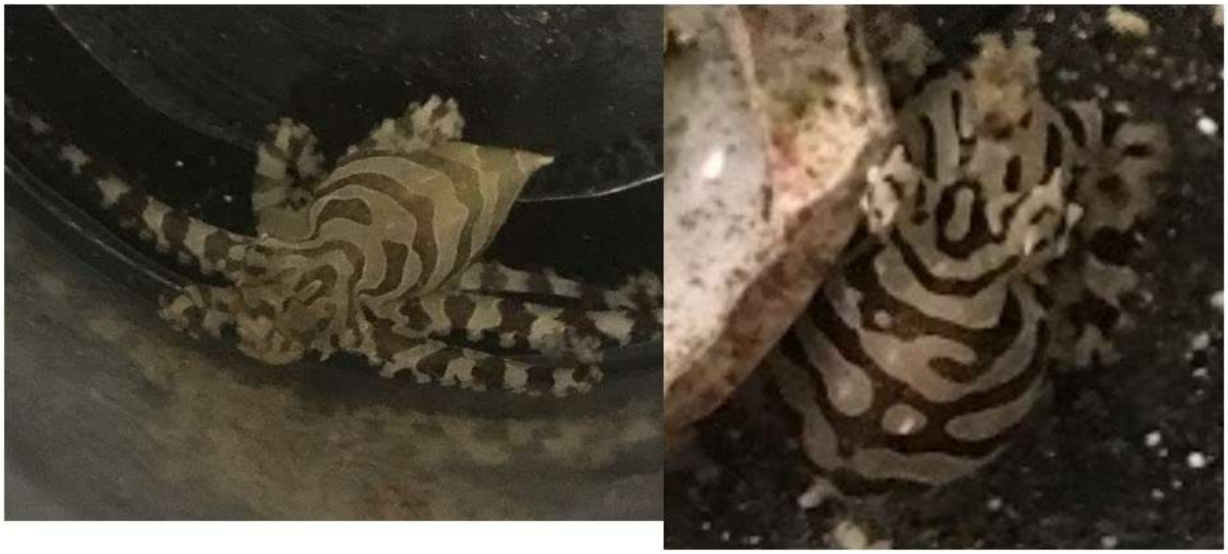

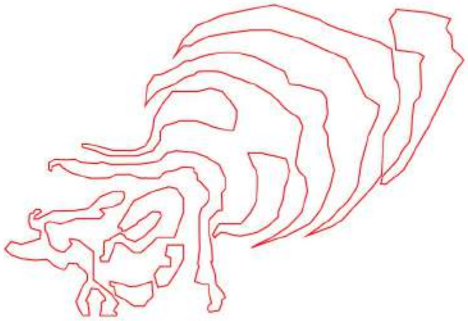

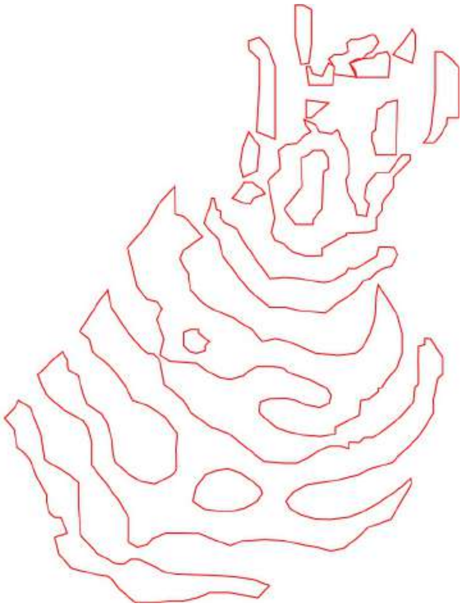

18.
Mark only one oval.

- ( ) match
- ( ) no match

19

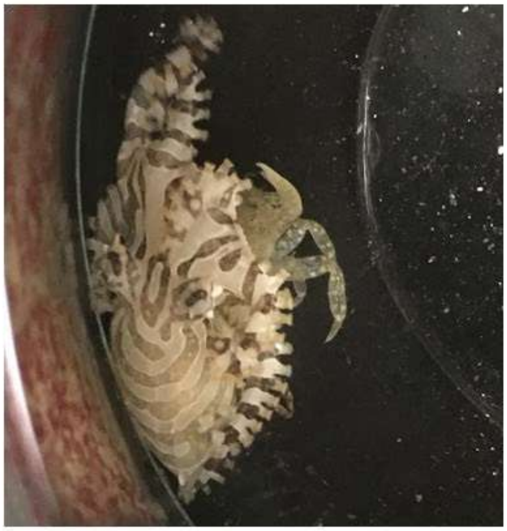

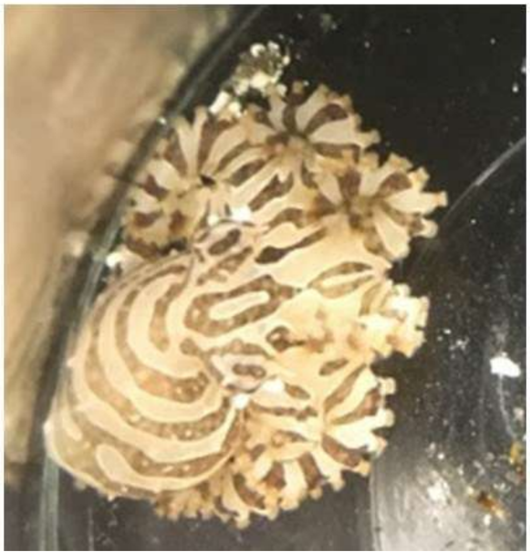

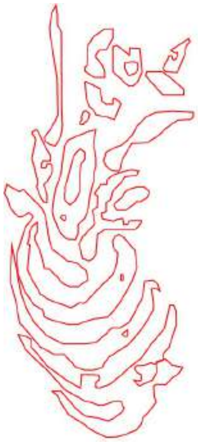

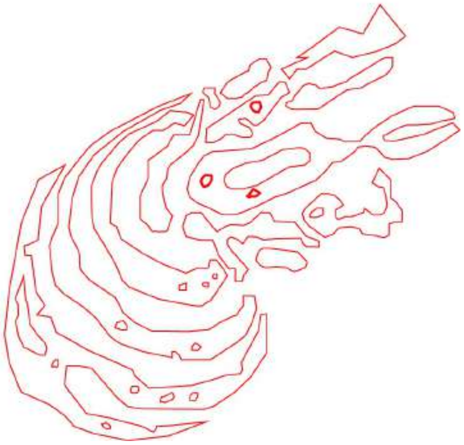

19.

Mark only one oval.

- match
- no match

20

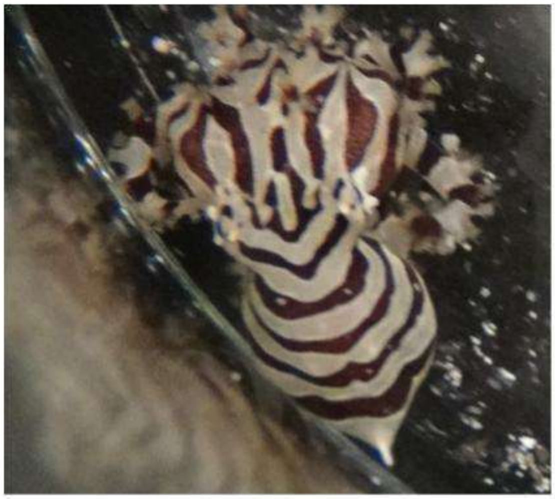

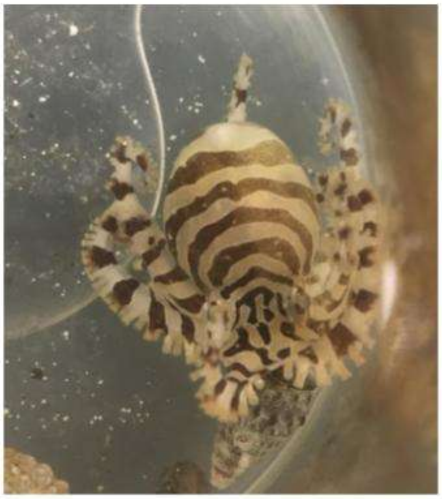

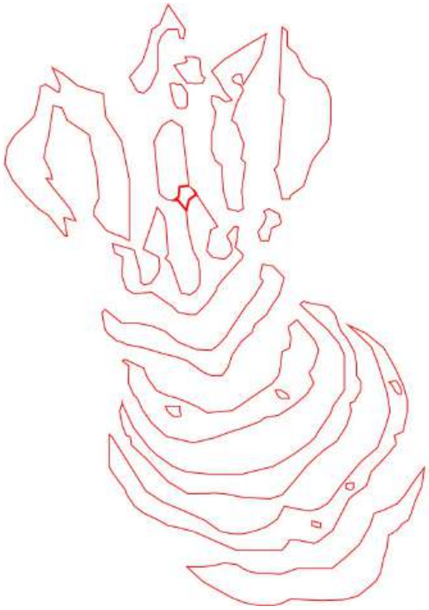

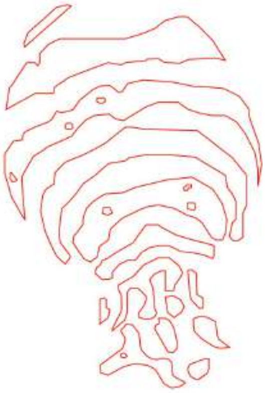

20.

Mark only one oval.

- match
- no match

21

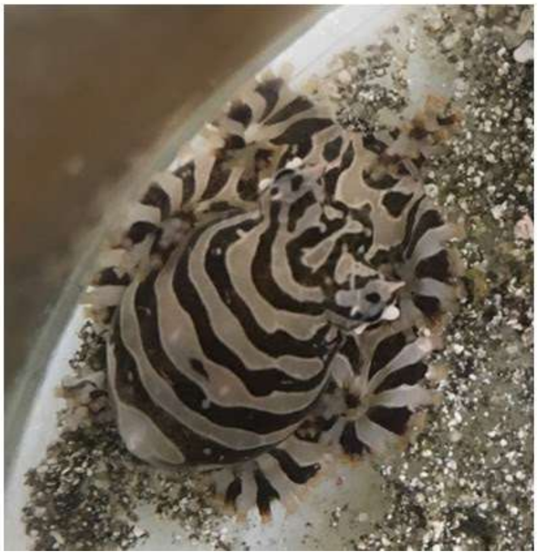

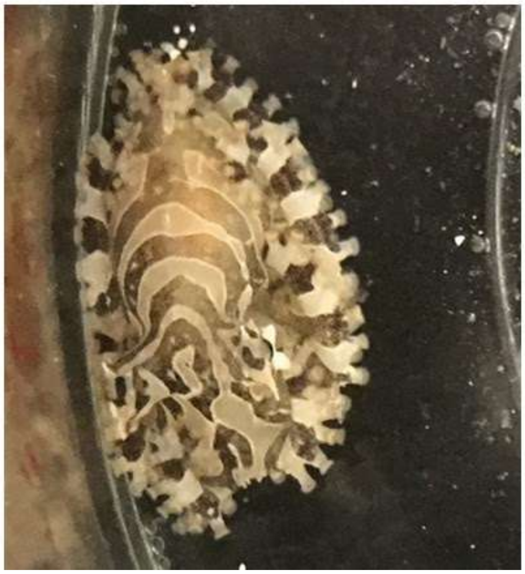

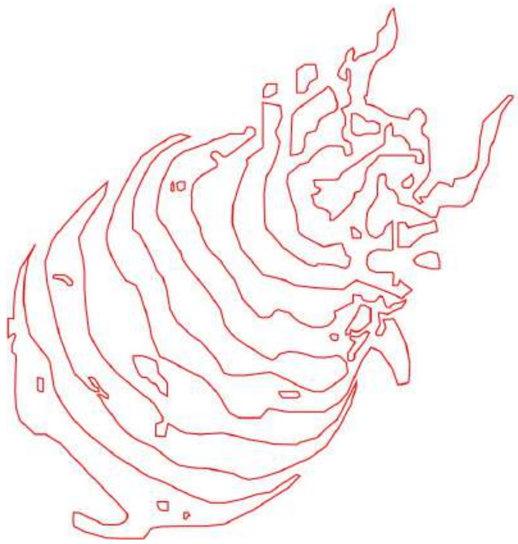

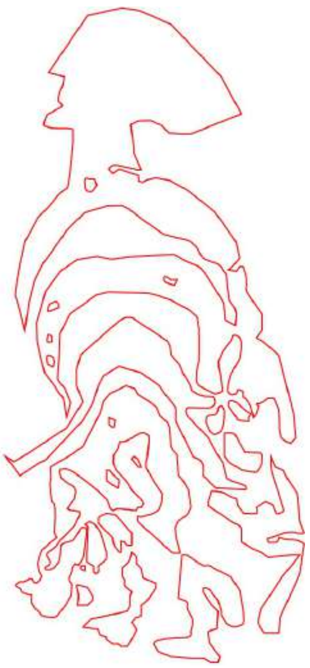

21.

Mark only one oval.

- ( ) match
- ( ) no match

22

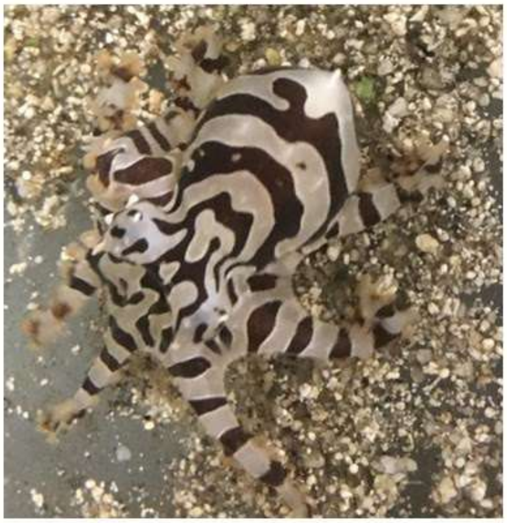

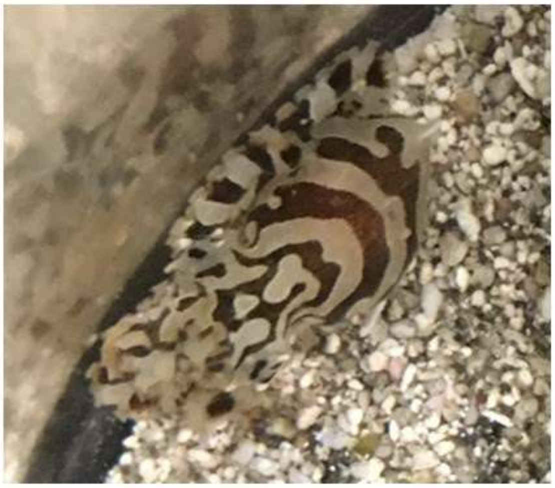

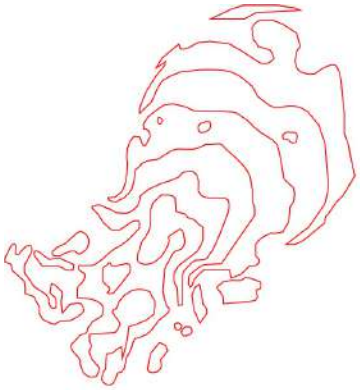

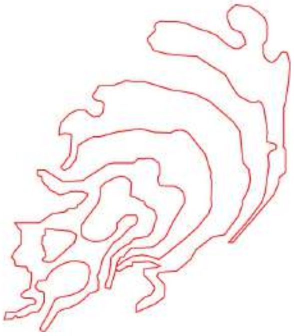

22.

Mark only one oval.

- match
- no match

23

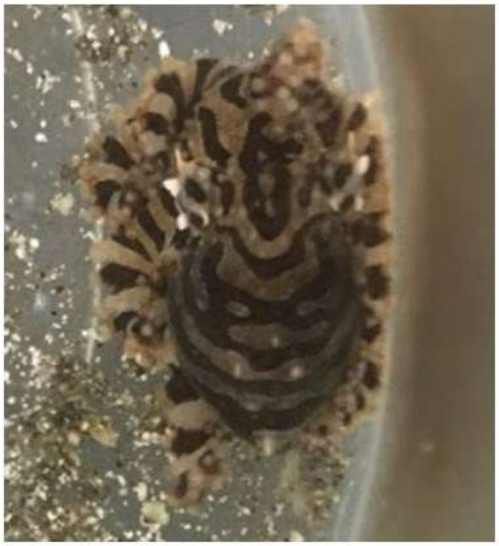

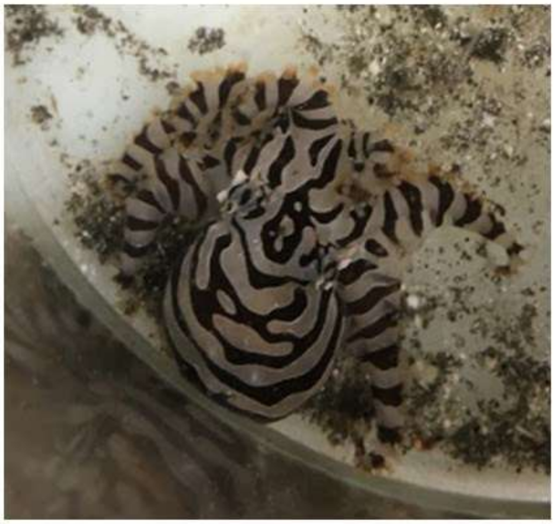

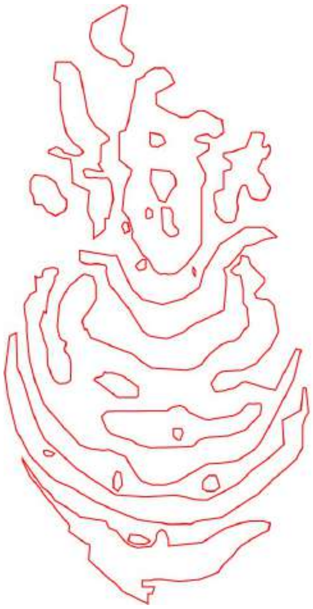

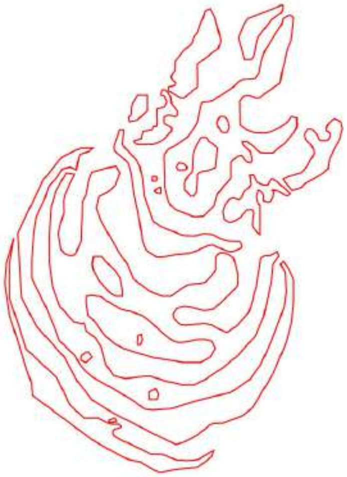

23.

Mark only one oval.

- match
- no match

24

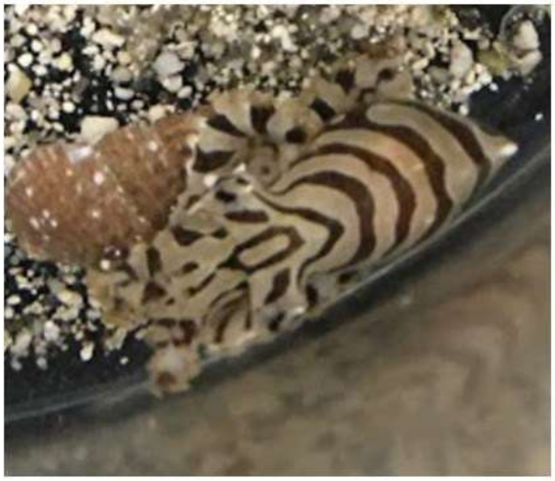

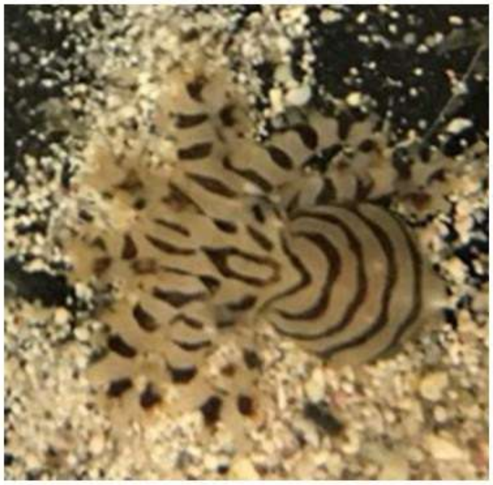

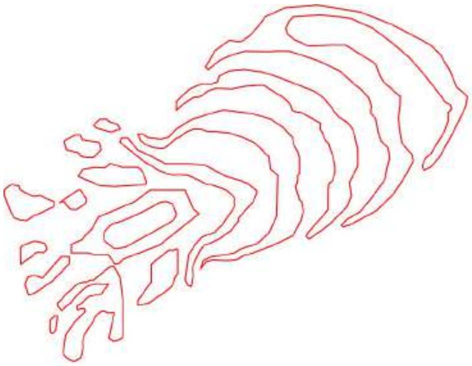

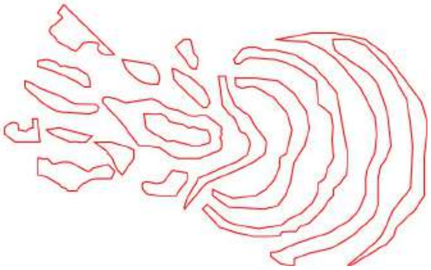

24. *Mark only one oval.*

- match
- no match

25

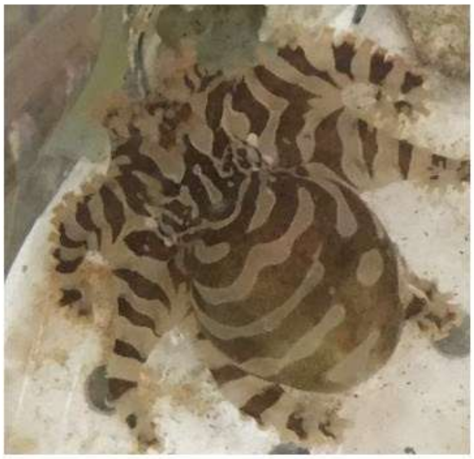

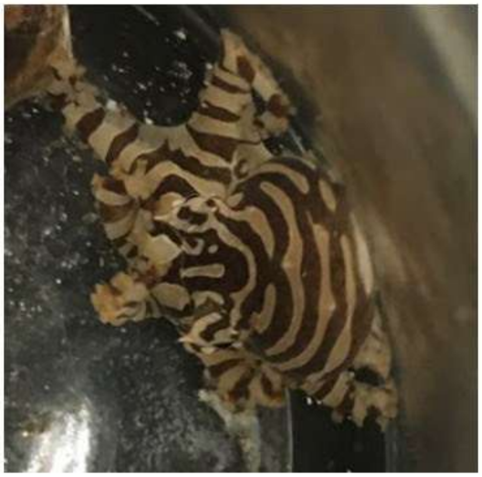

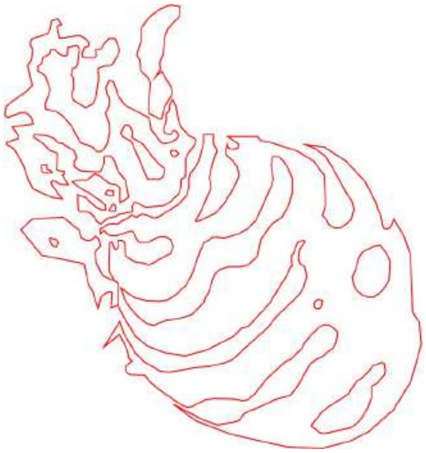

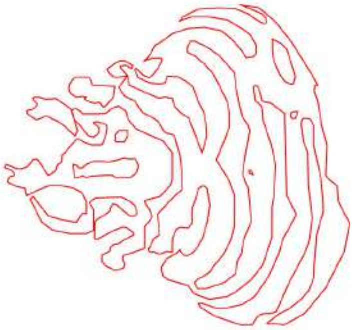

25.

Mark only one oval.

- match
- no match

26

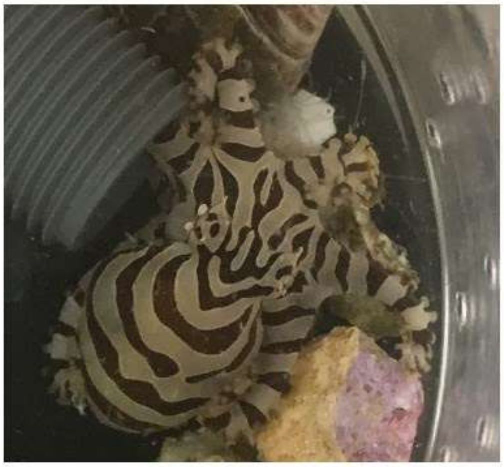

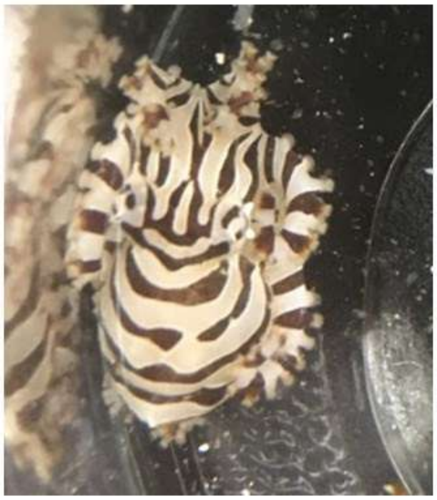

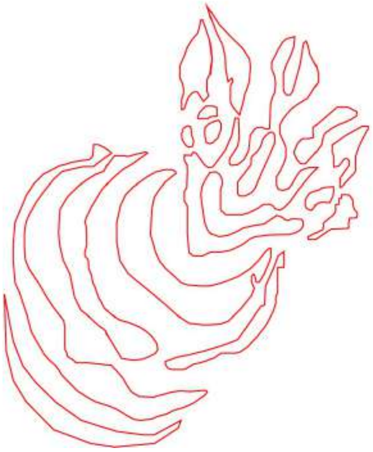

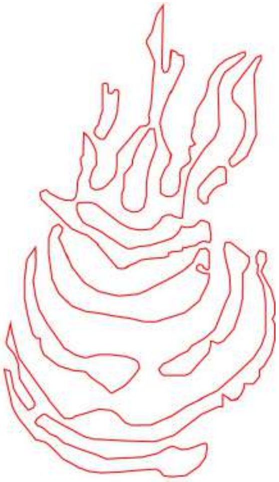

26. *Mark only one oval.*

- ( ) match
- ( ) no match

27

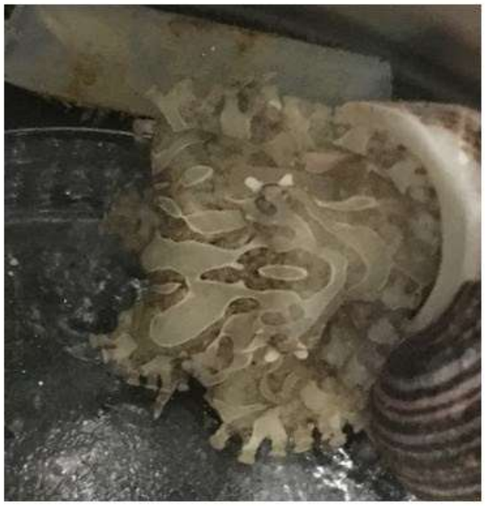
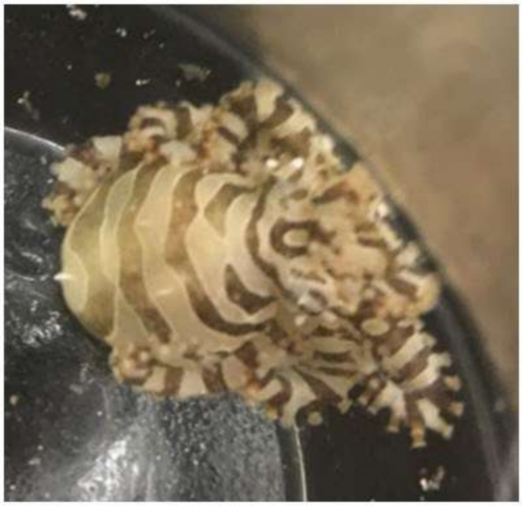
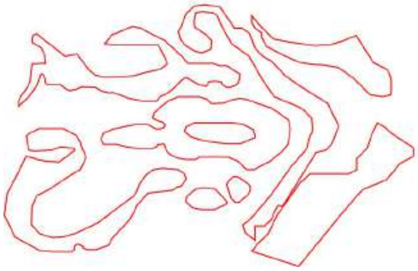
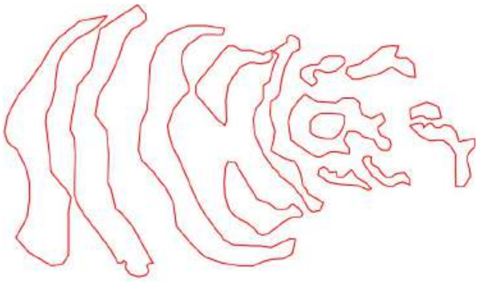

27.

Mark only one oval.

- match
- no match

28

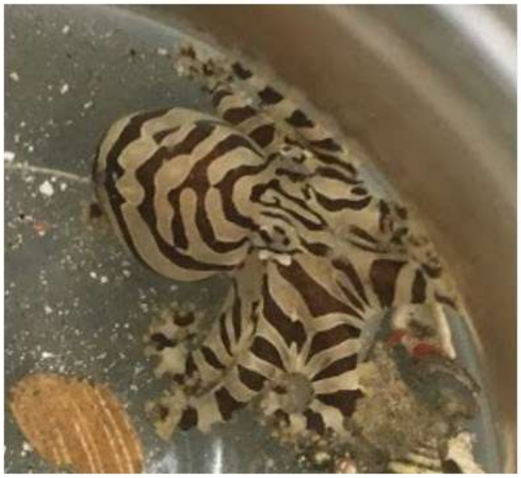

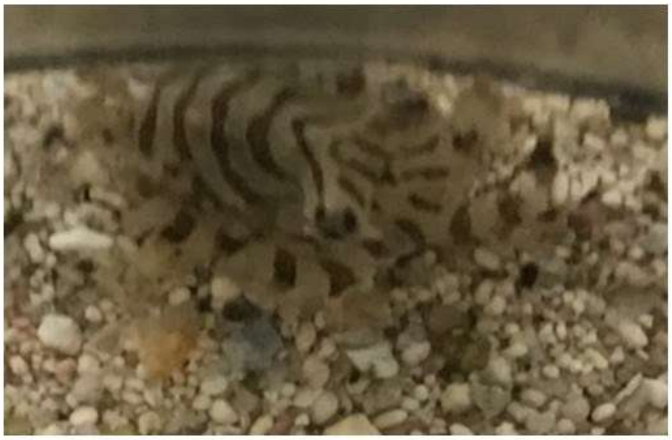

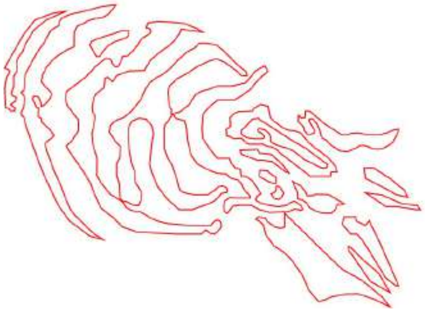

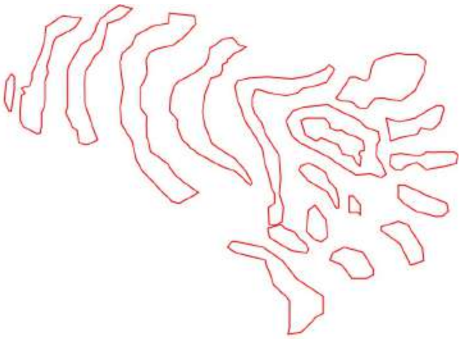

28.

Mark only one oval.

- match
- no match

29

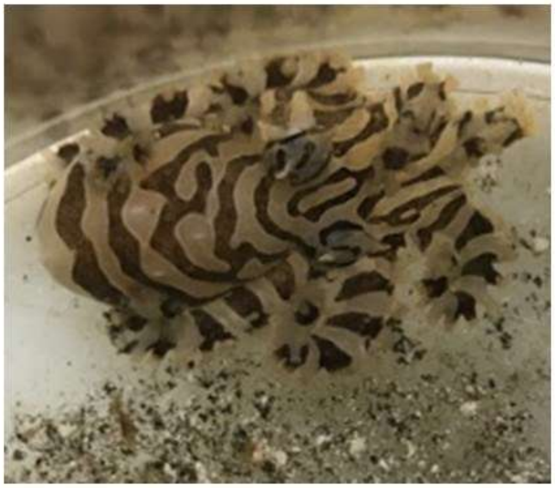

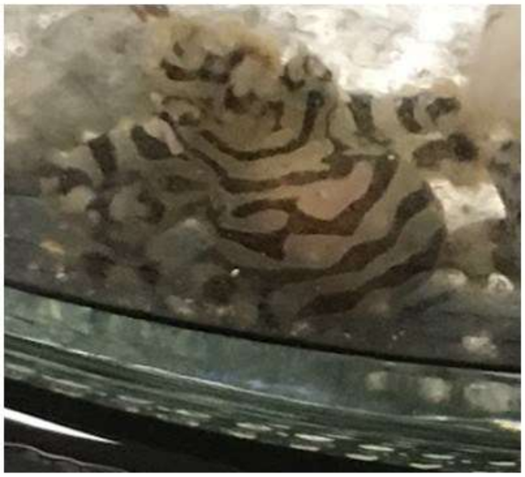

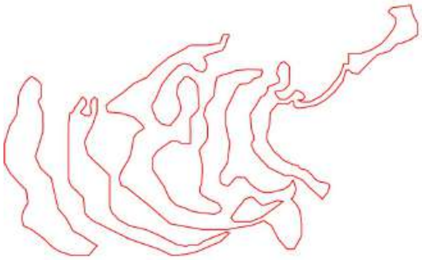

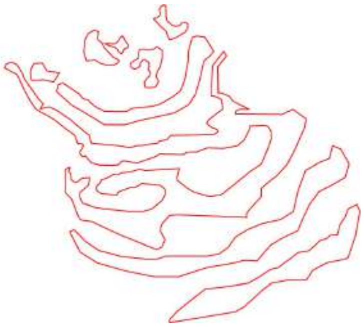

29.

Mark only one oval.

- match
- no match

30

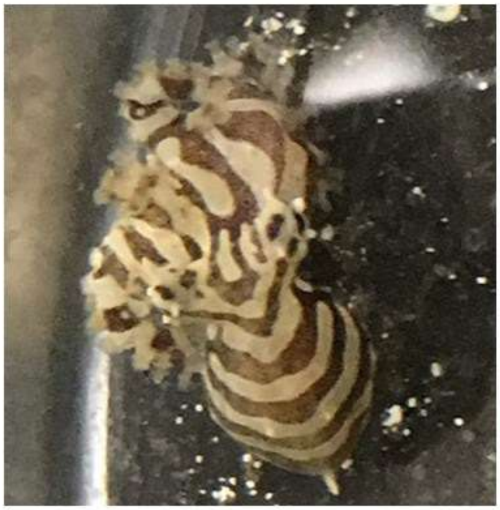

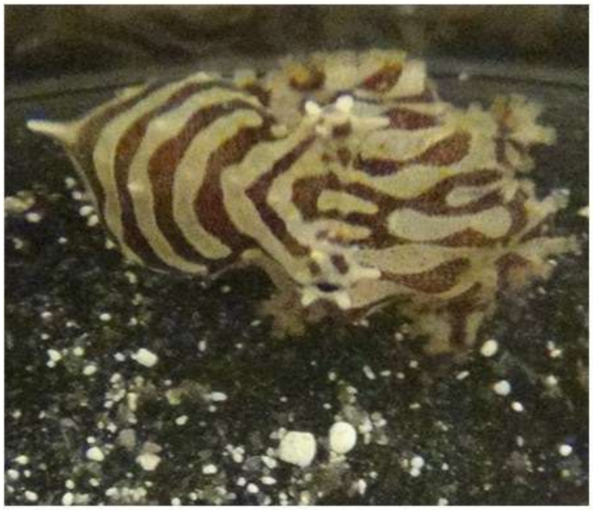

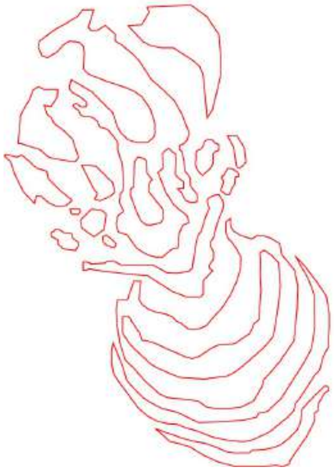

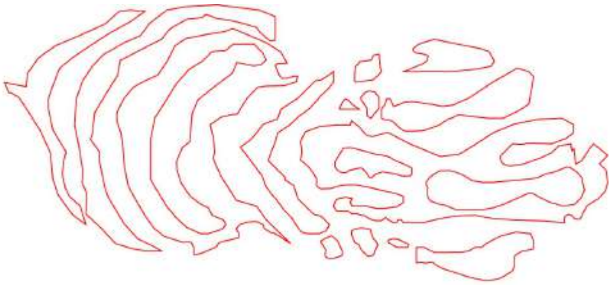

30. *Mark only one oval.*

- [ ] match
- [ ] no match

31

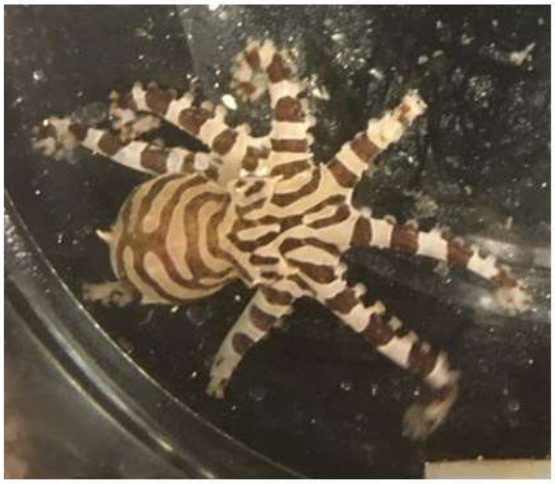
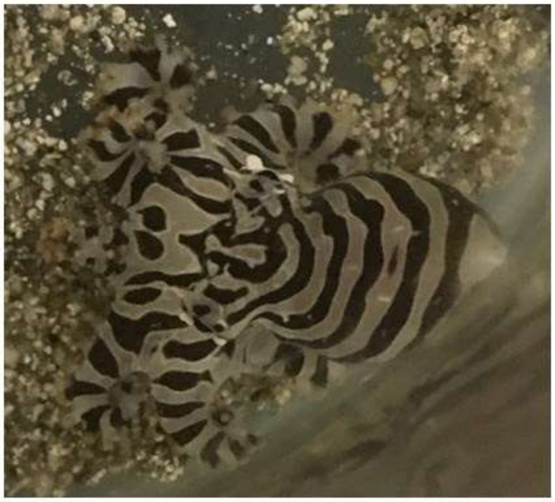
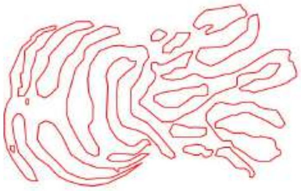
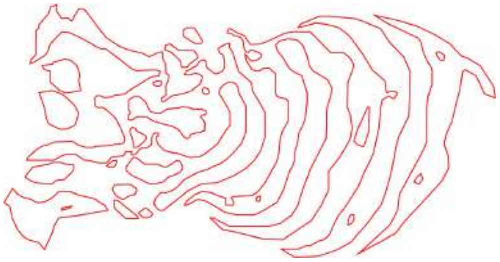

31.

Mark only one oval.

- match
- no match

32

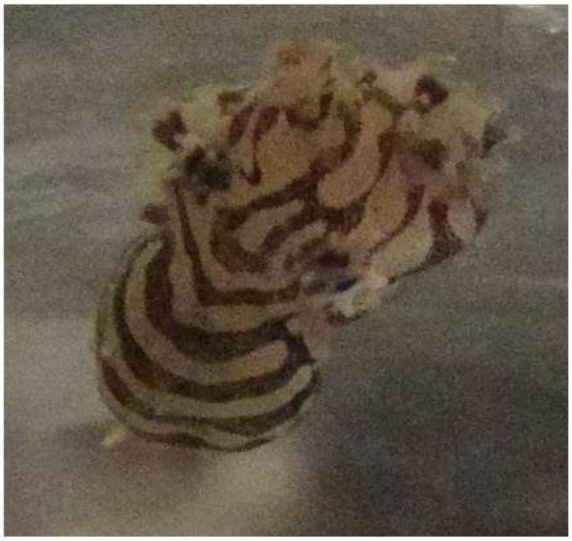
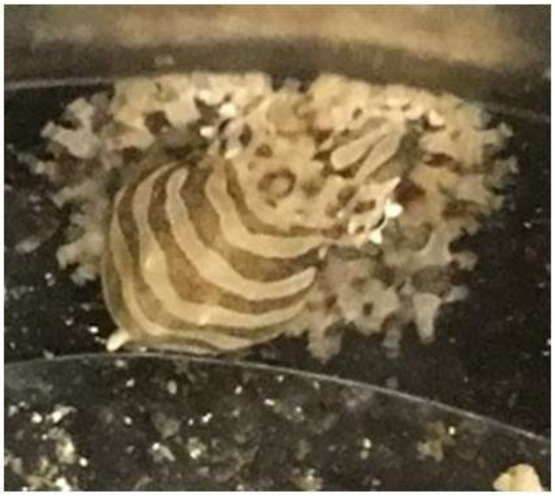

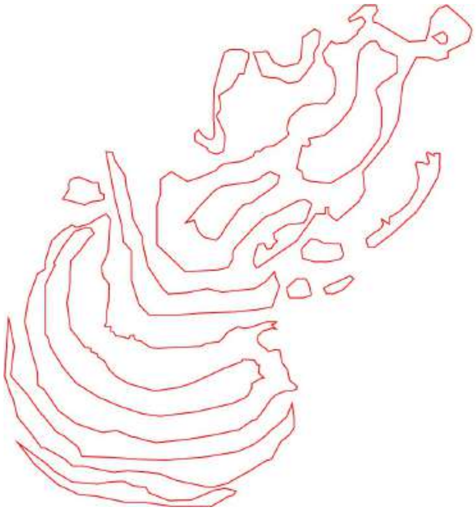
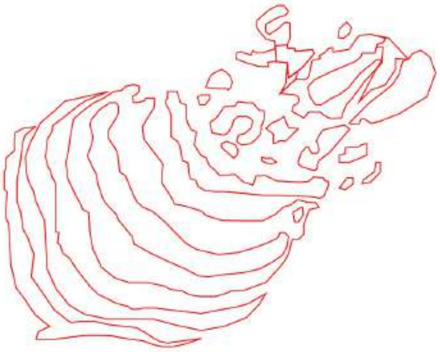

32.

Mark only one oval.

- match
- no match

33

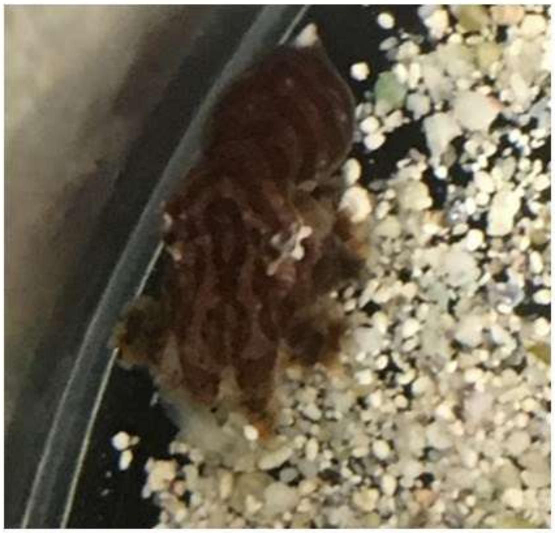

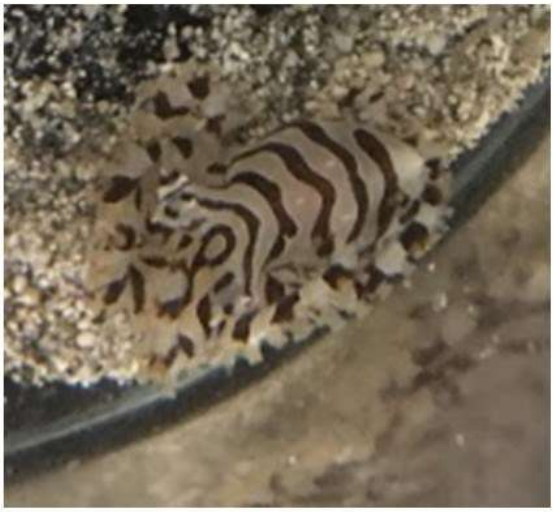

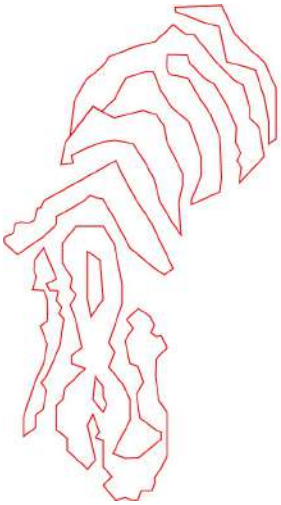

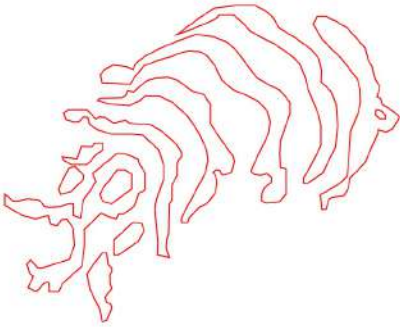

33.

Mark only one oval.

- match
- no match

34

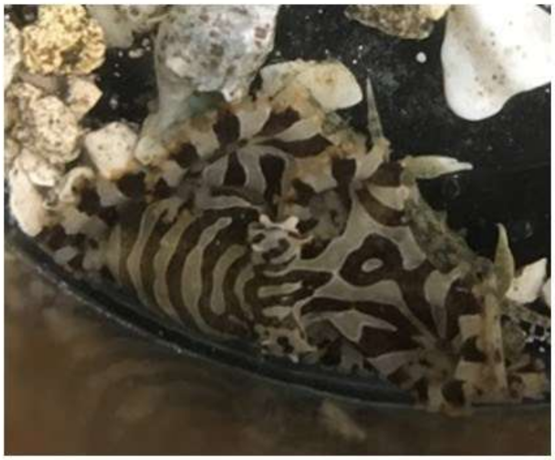
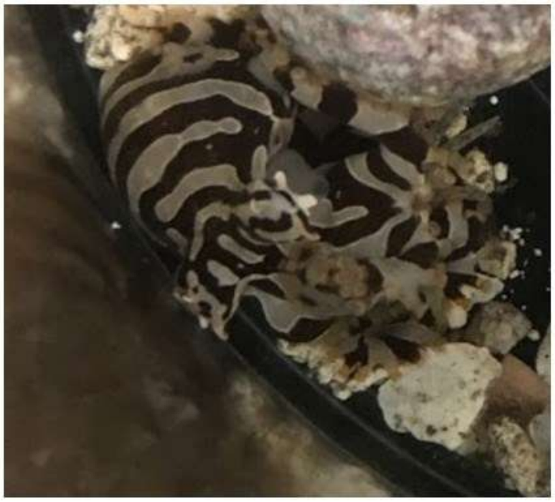

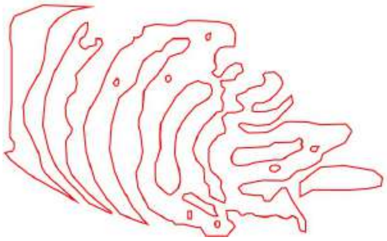
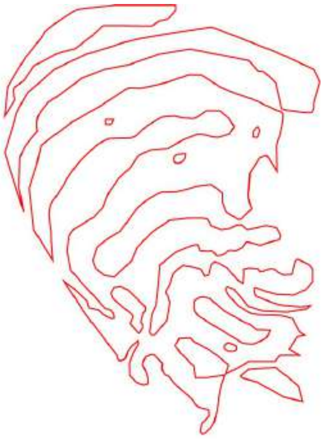

34.
Mark only one oval.

- match
- no match

35

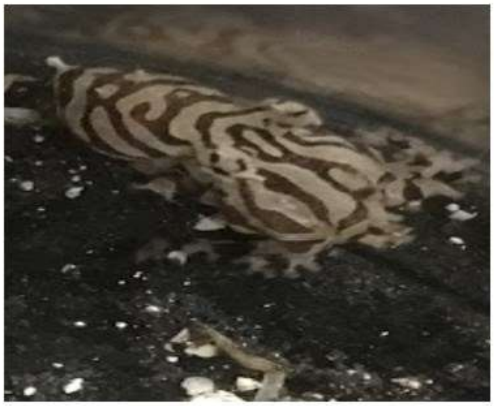

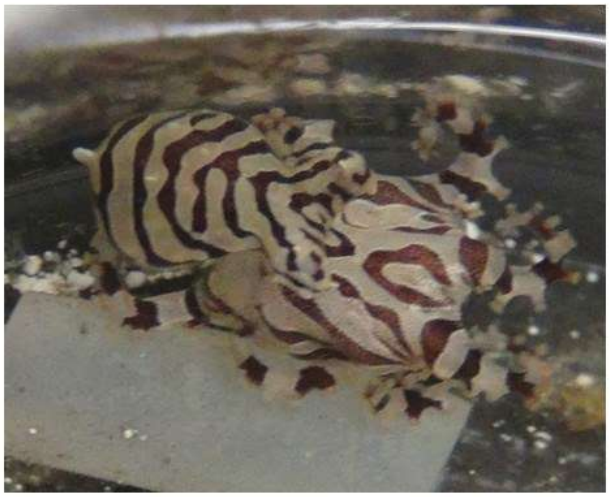

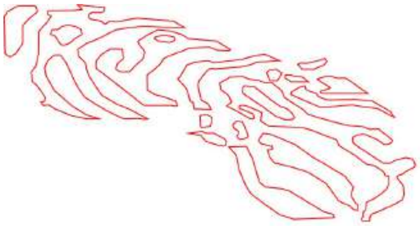

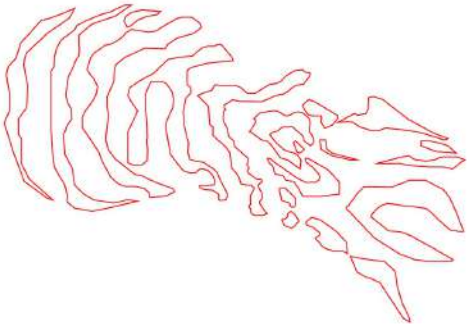

35. *Mark only one oval.*

- match
- no match

36

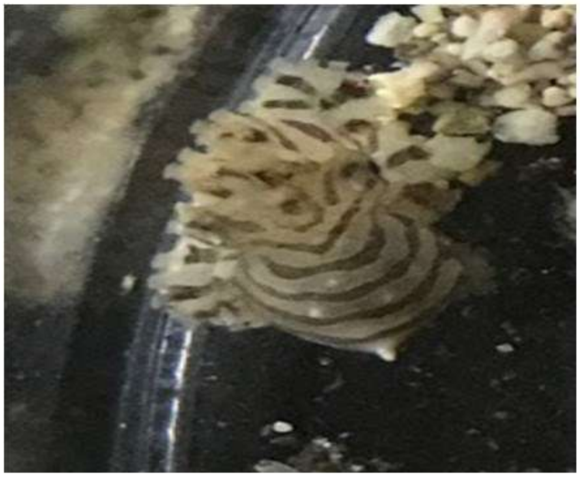

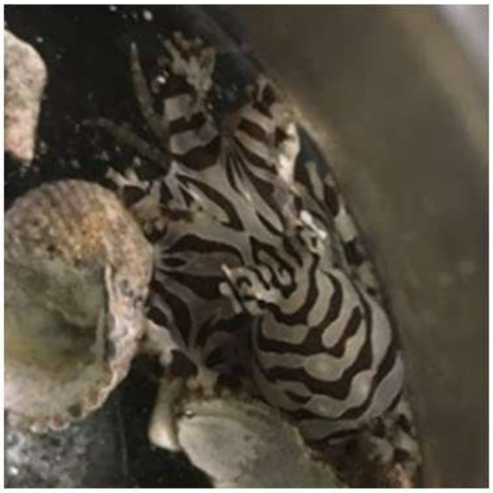

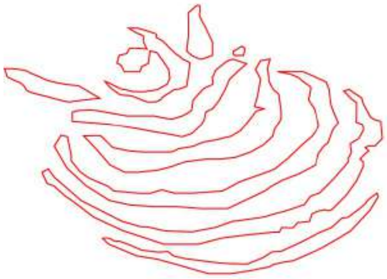

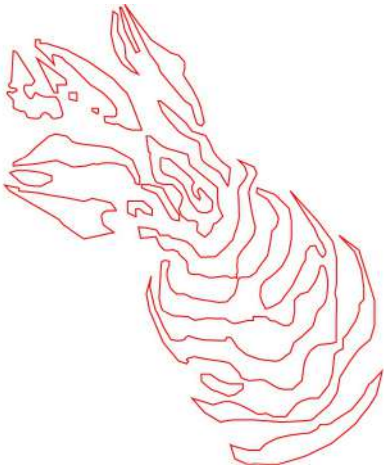

36.

Mark only one oval.

- match
- no match

37

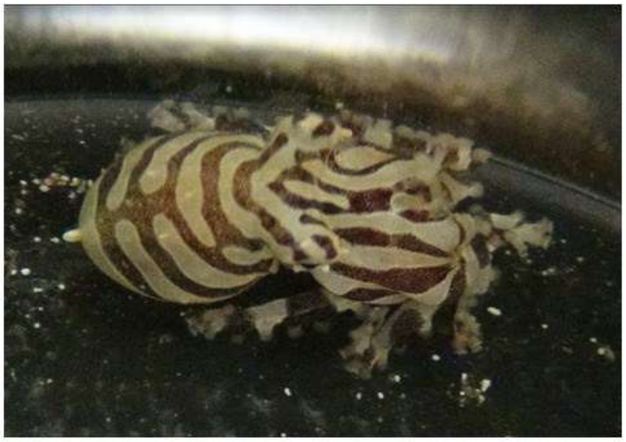

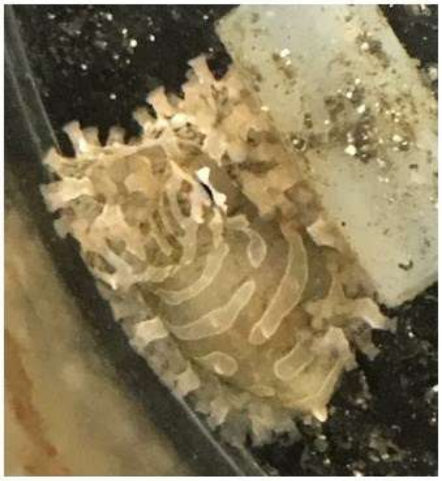

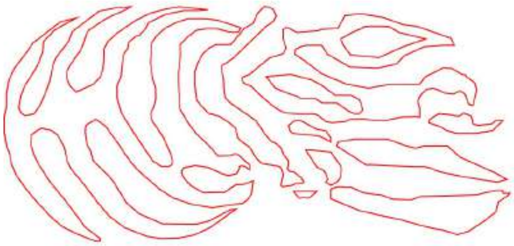

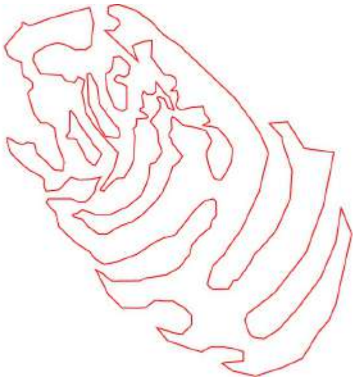

37. *Mark only one oval.*

- match
- no match

38

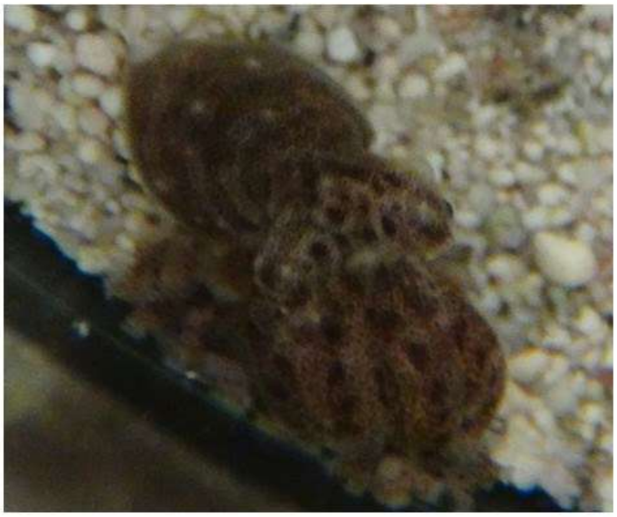

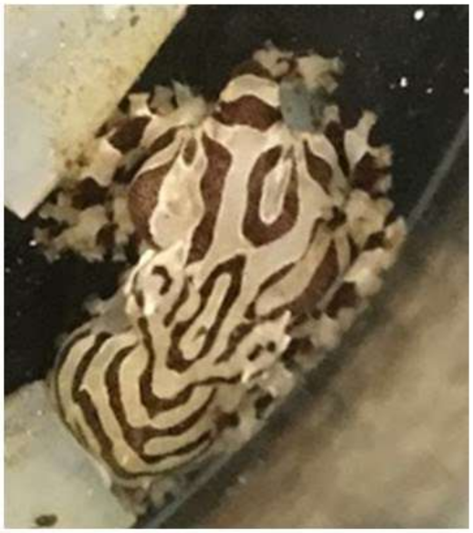

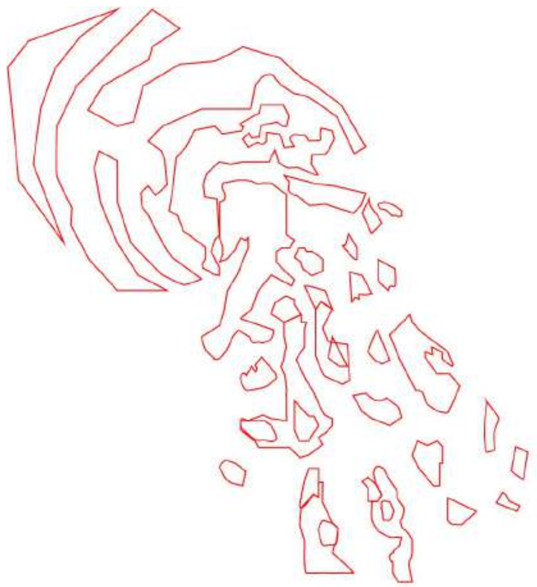

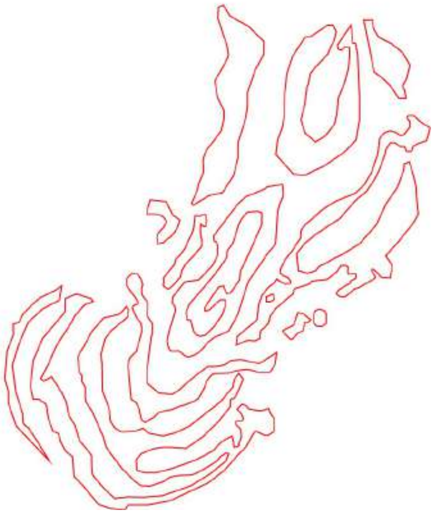

38.

Mark only one oval.

- ( ) match
- ( ) no match

39

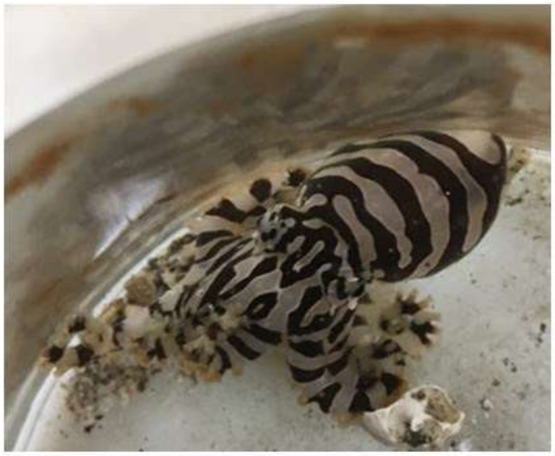

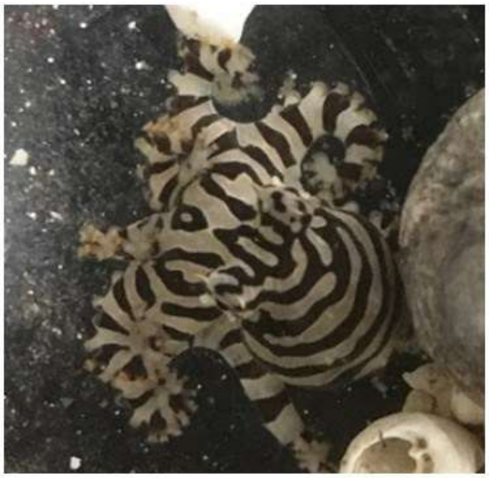

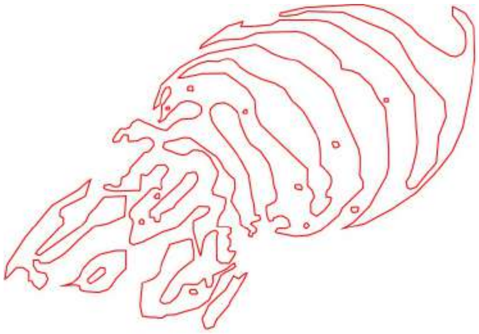

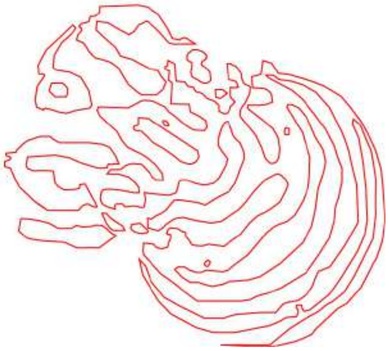

39.
Mark only one oval.

- match
- no match

40

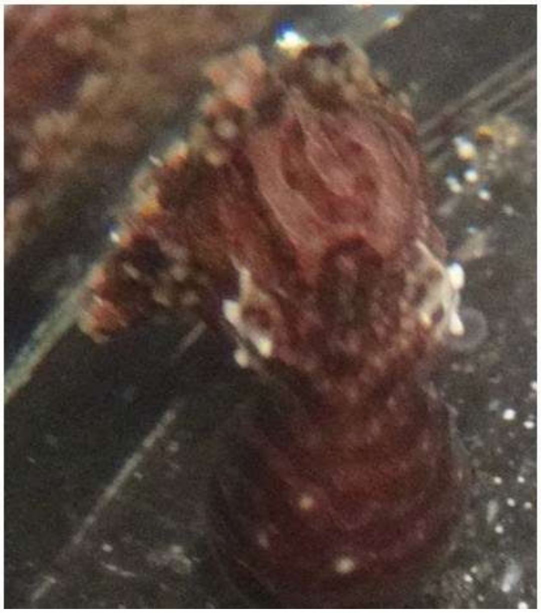

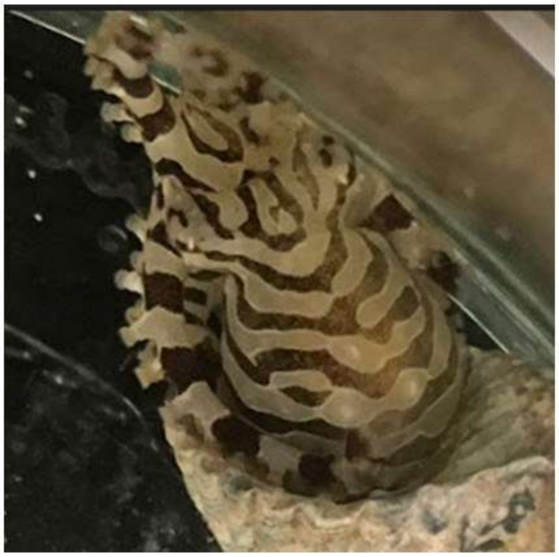

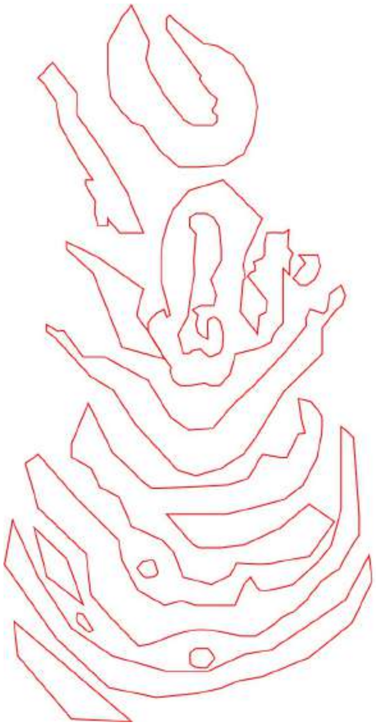

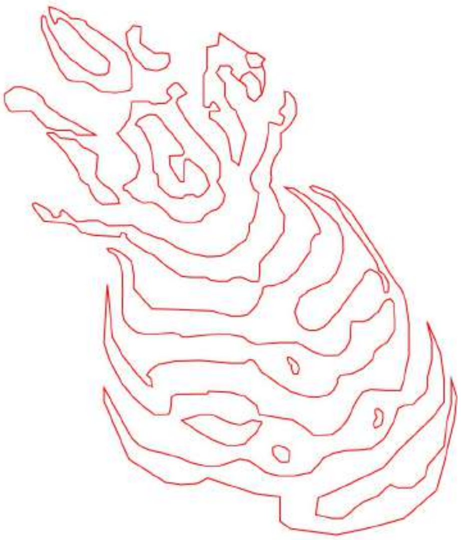

40.
Mark only one oval.

- match
- no match

41

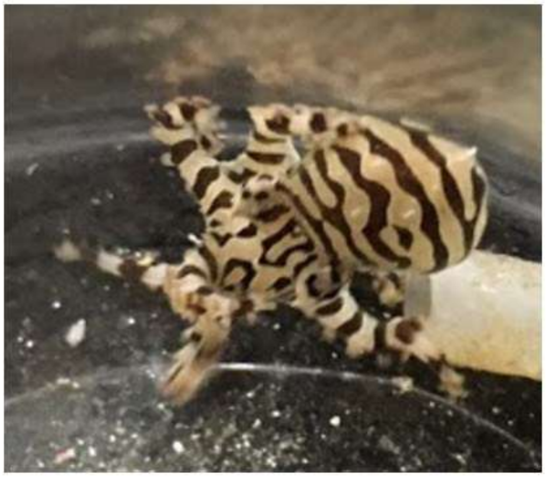

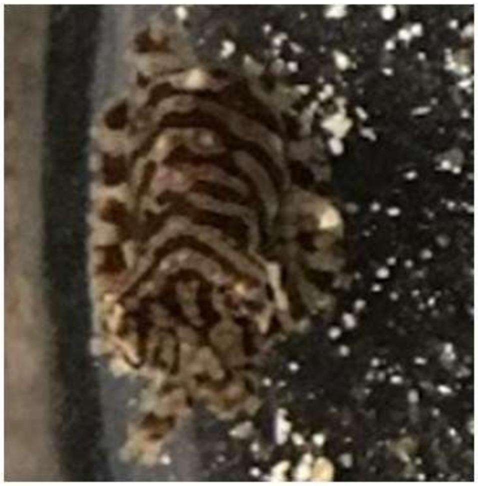

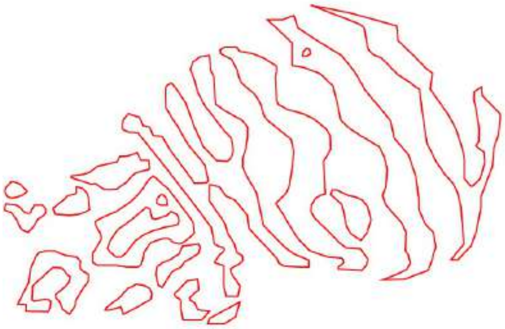

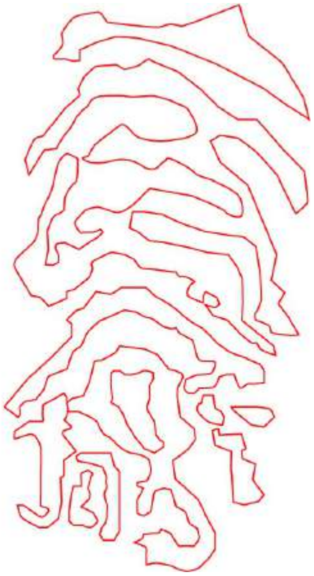

41.

Mark only one oval.

- match
- no match

42

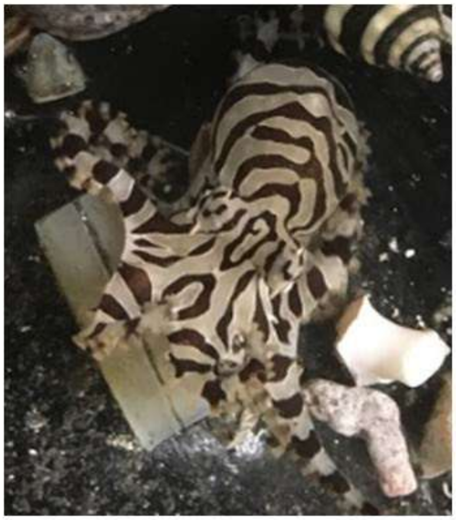

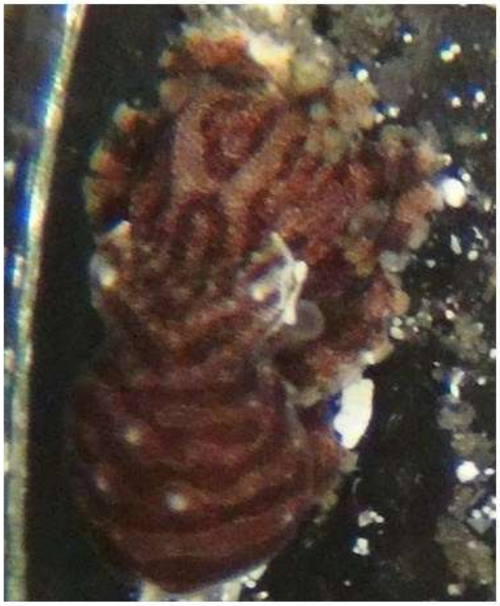

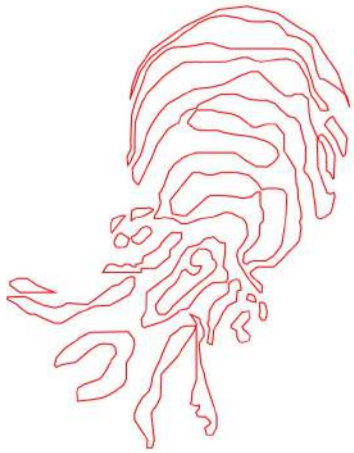

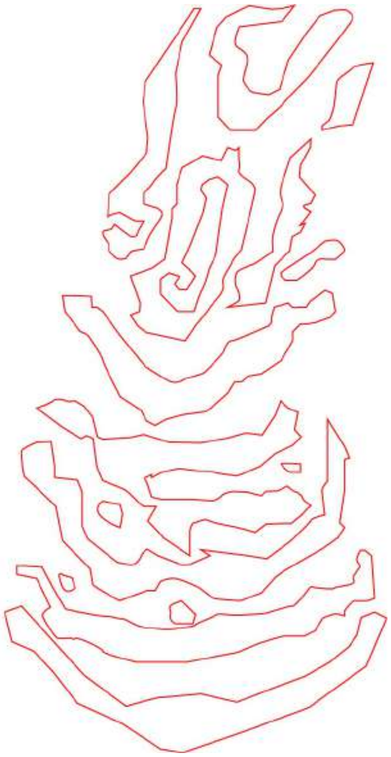

42.

Mark only one oval.

- match
- no match

43

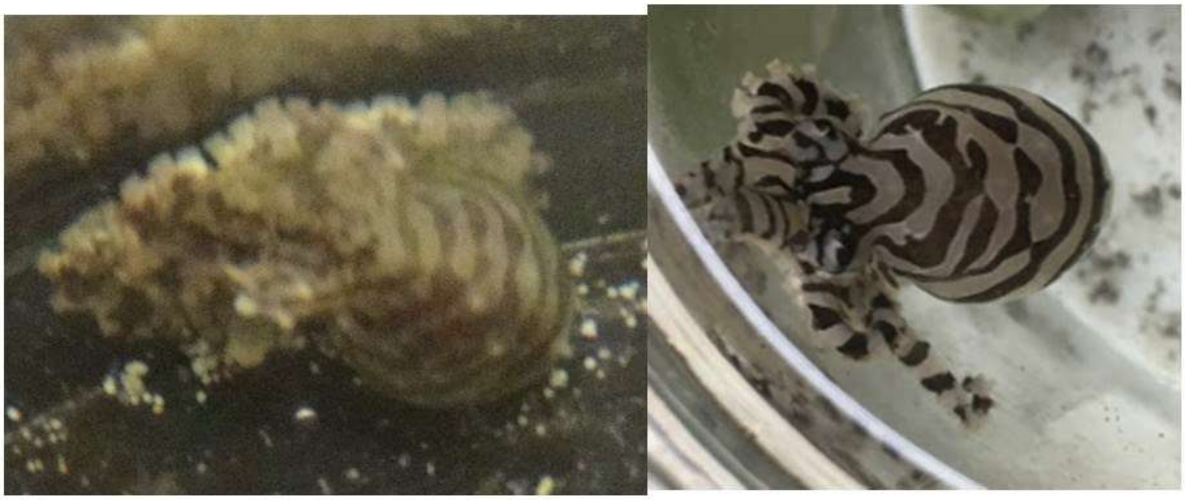

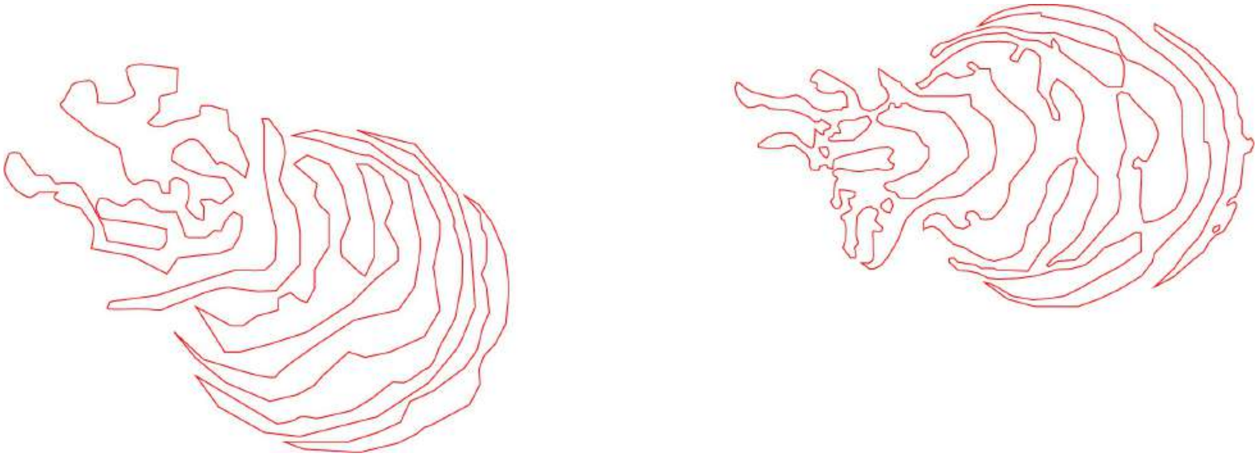

43.

Mark only one oval.

- ⬭ match
- ⬭ no match

44

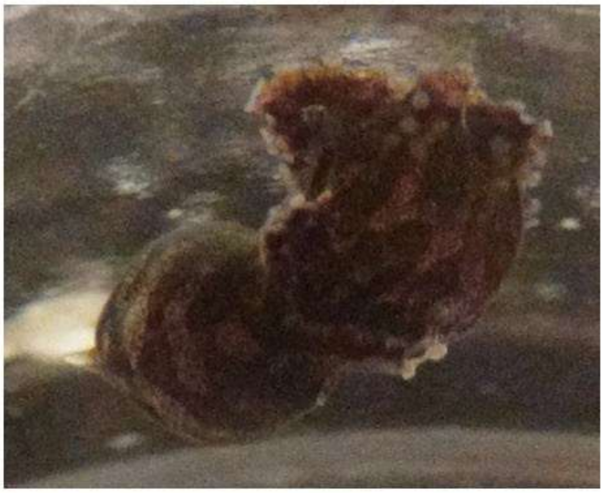

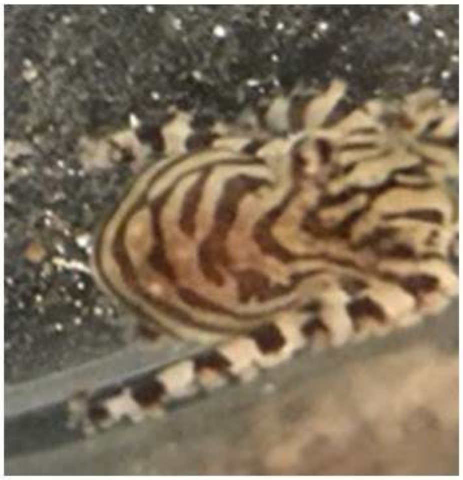

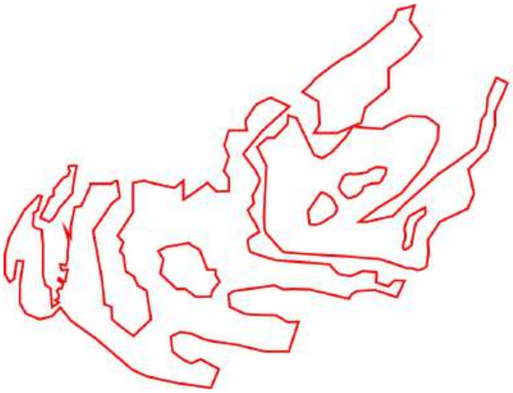

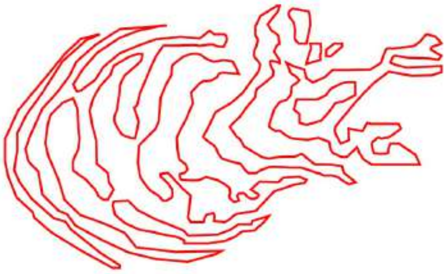

44.

Mark only one oval.

- match
- no match

45

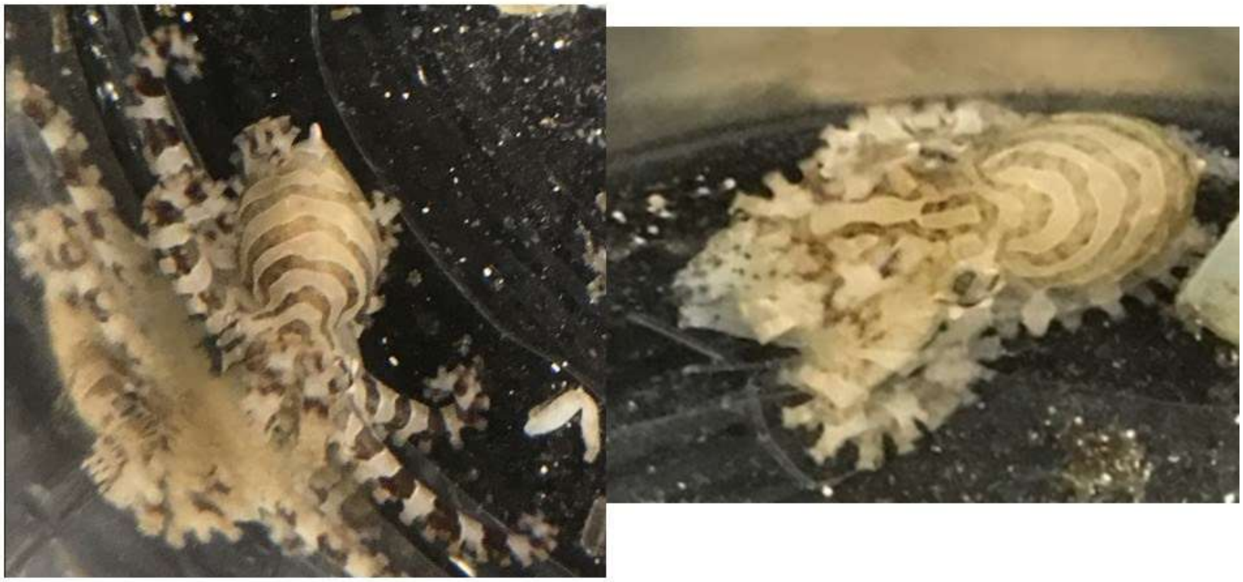

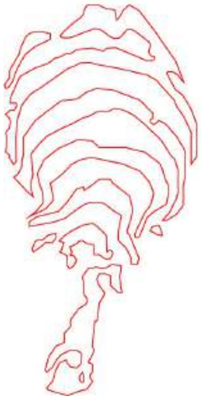

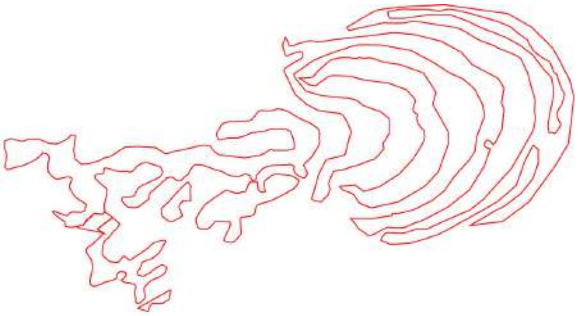

45.

Mark only one oval.

- match
- no match

47

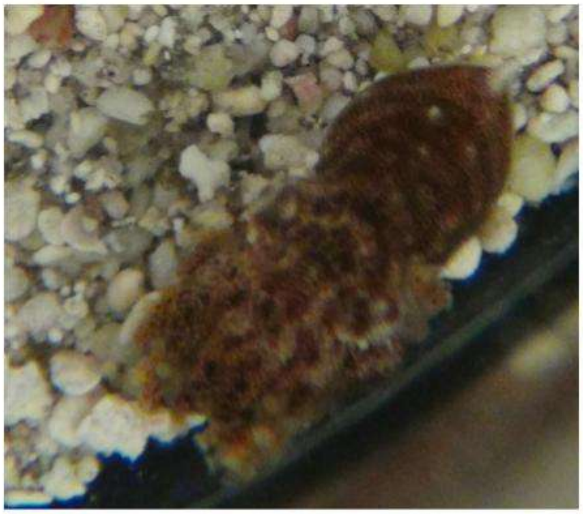

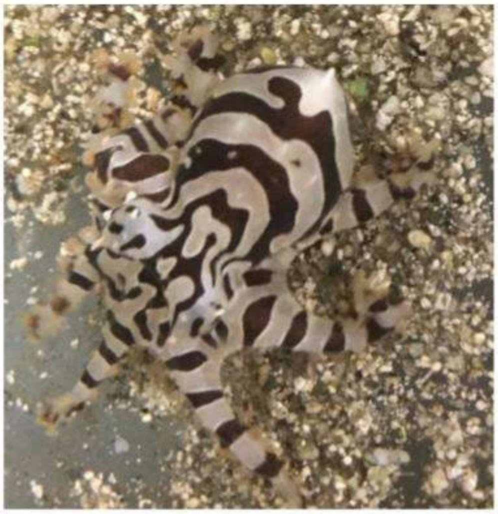

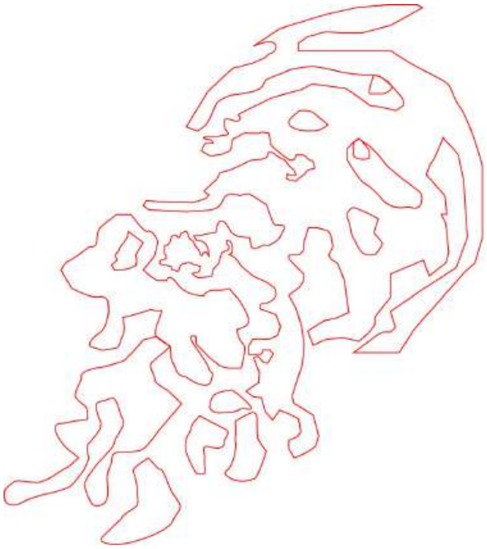

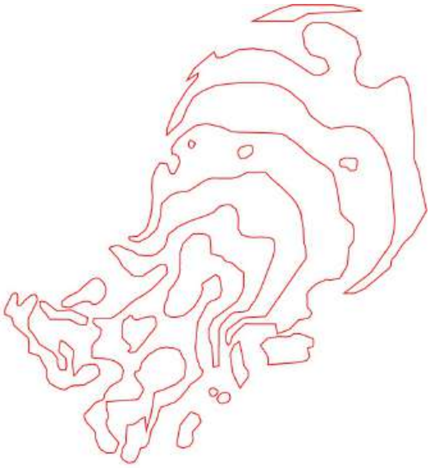

46.

Mark only one oval.

- match
- no match

47

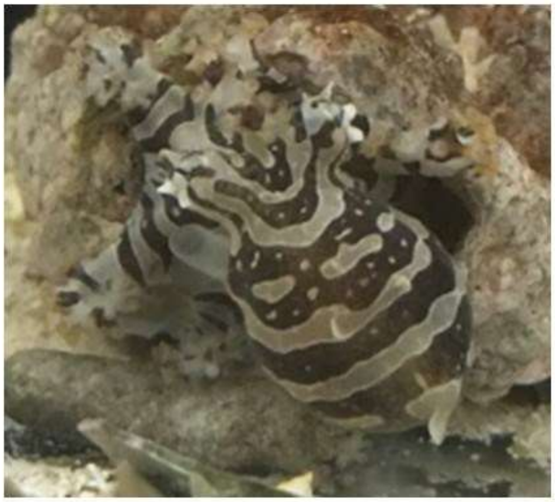

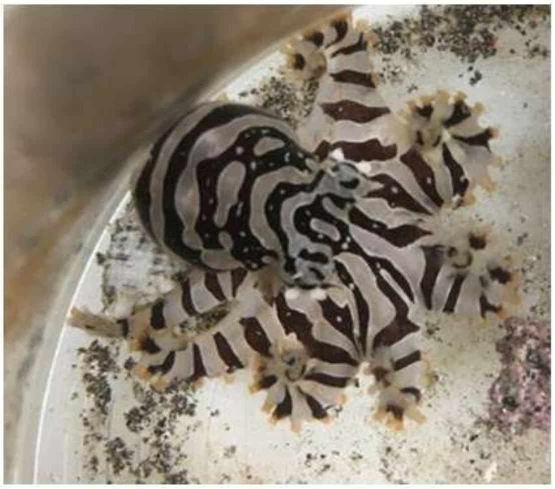

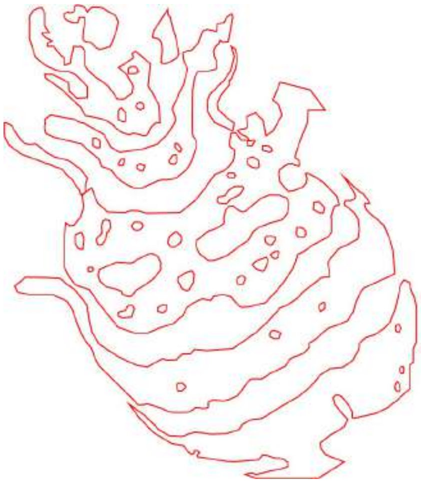

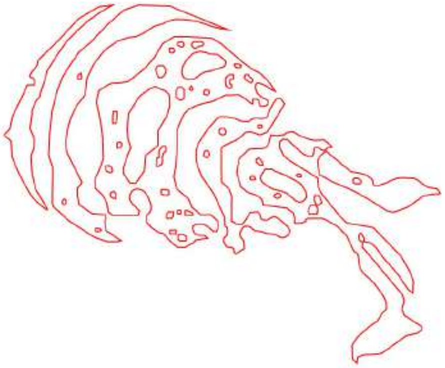

47.
Mark only one oval.

- match
- no match

48

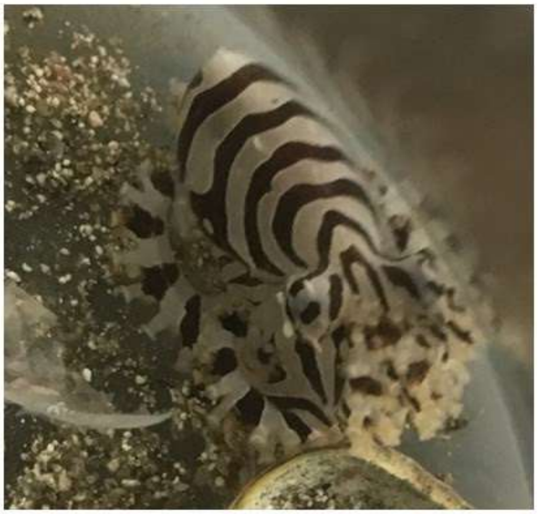

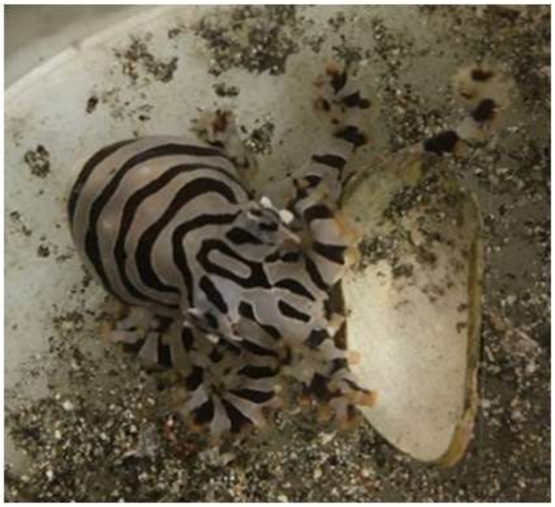

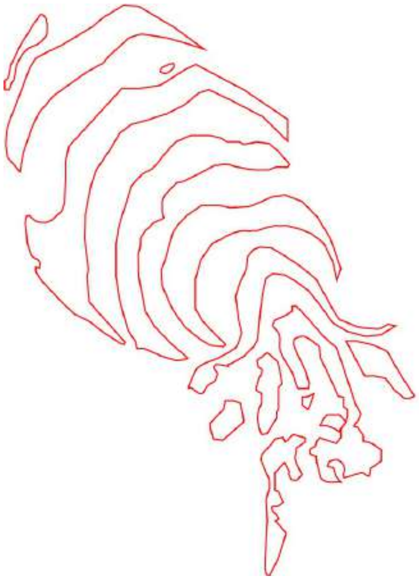

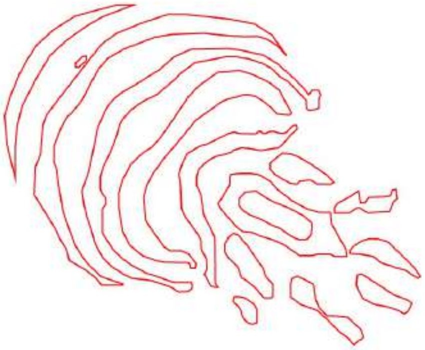

48.

Mark only one oval.

- match
- no match

49

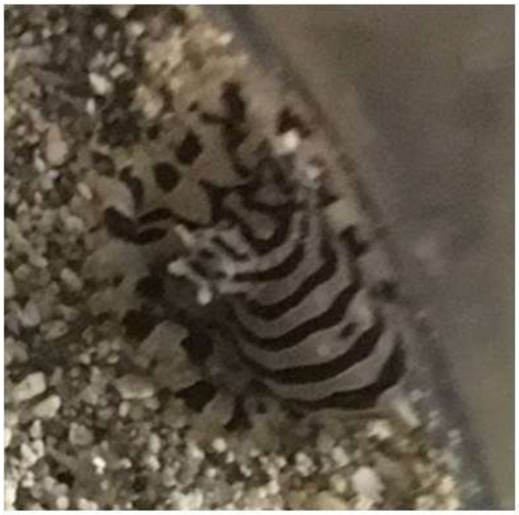

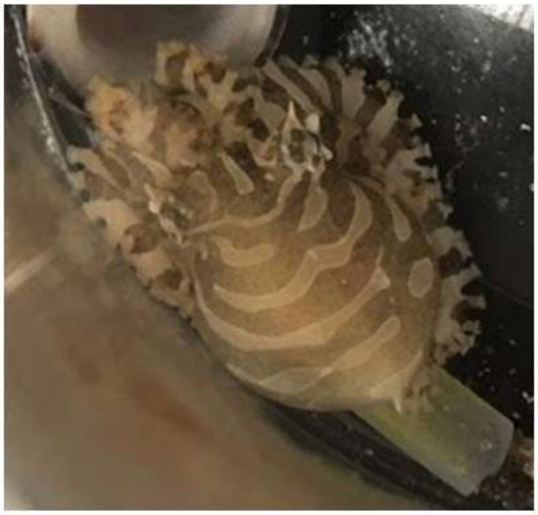

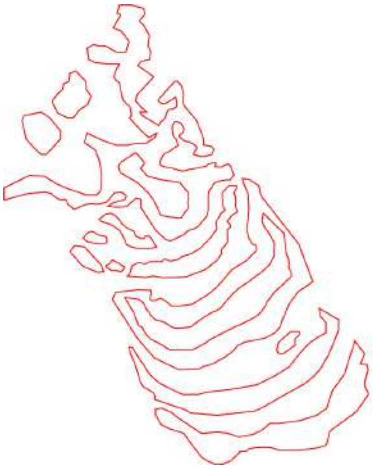

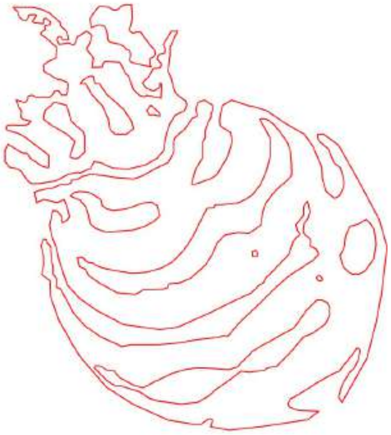

49.

Mark only one oval.

- ◯ match
- ◯ no match

50

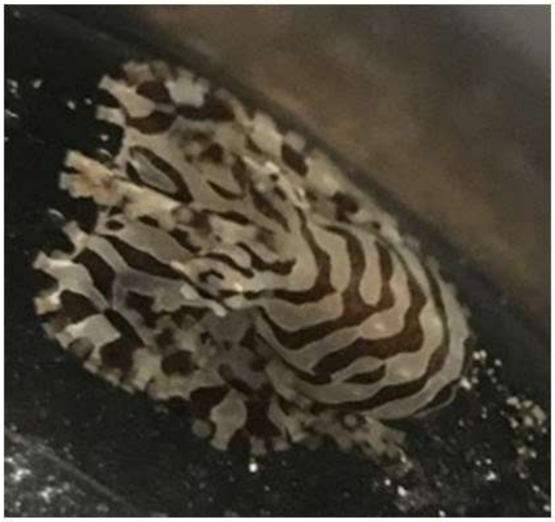

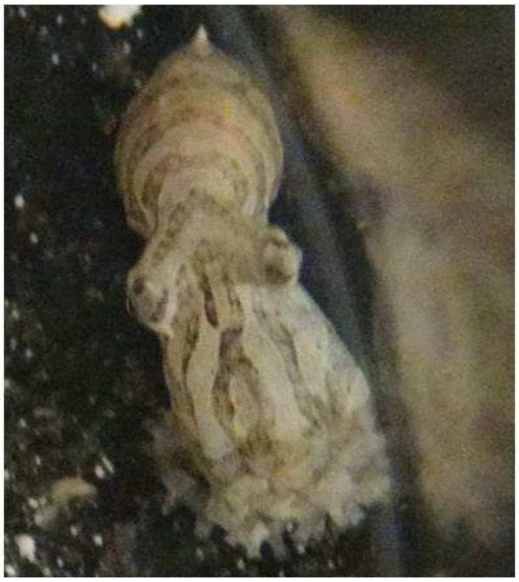

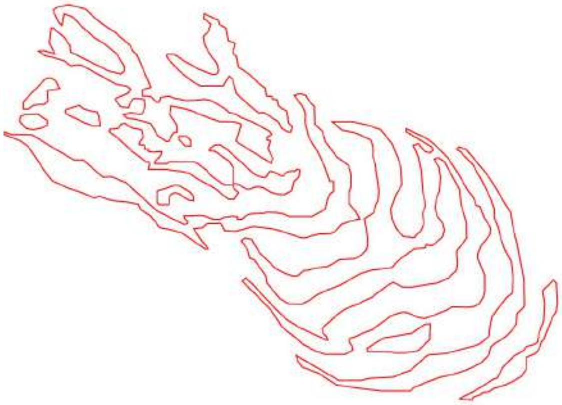

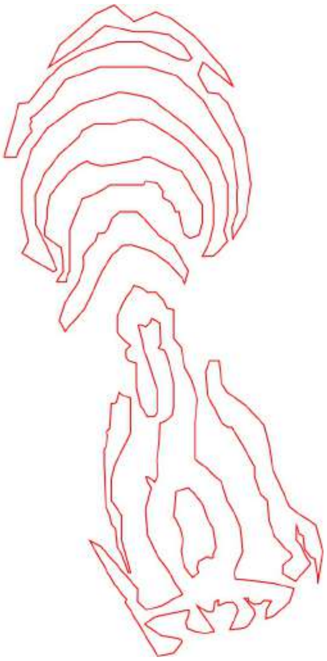

50.

Mark only one oval.

- ◯ match
- ◯ no match

This content is neither created nor endorsed by Google.

Google Forms
