# Supplementary figures and images for: Individually unique, fixed stripe configurations of Octopus chierchiae allow for photoidentification in long-term studies
Source: PLoS One. 2023 Apr 12;18(4):e0265292. doi: 10.1371/journal.pone.0265292 (PMC10096297; doi:10.1371/journal.pone.0265292)

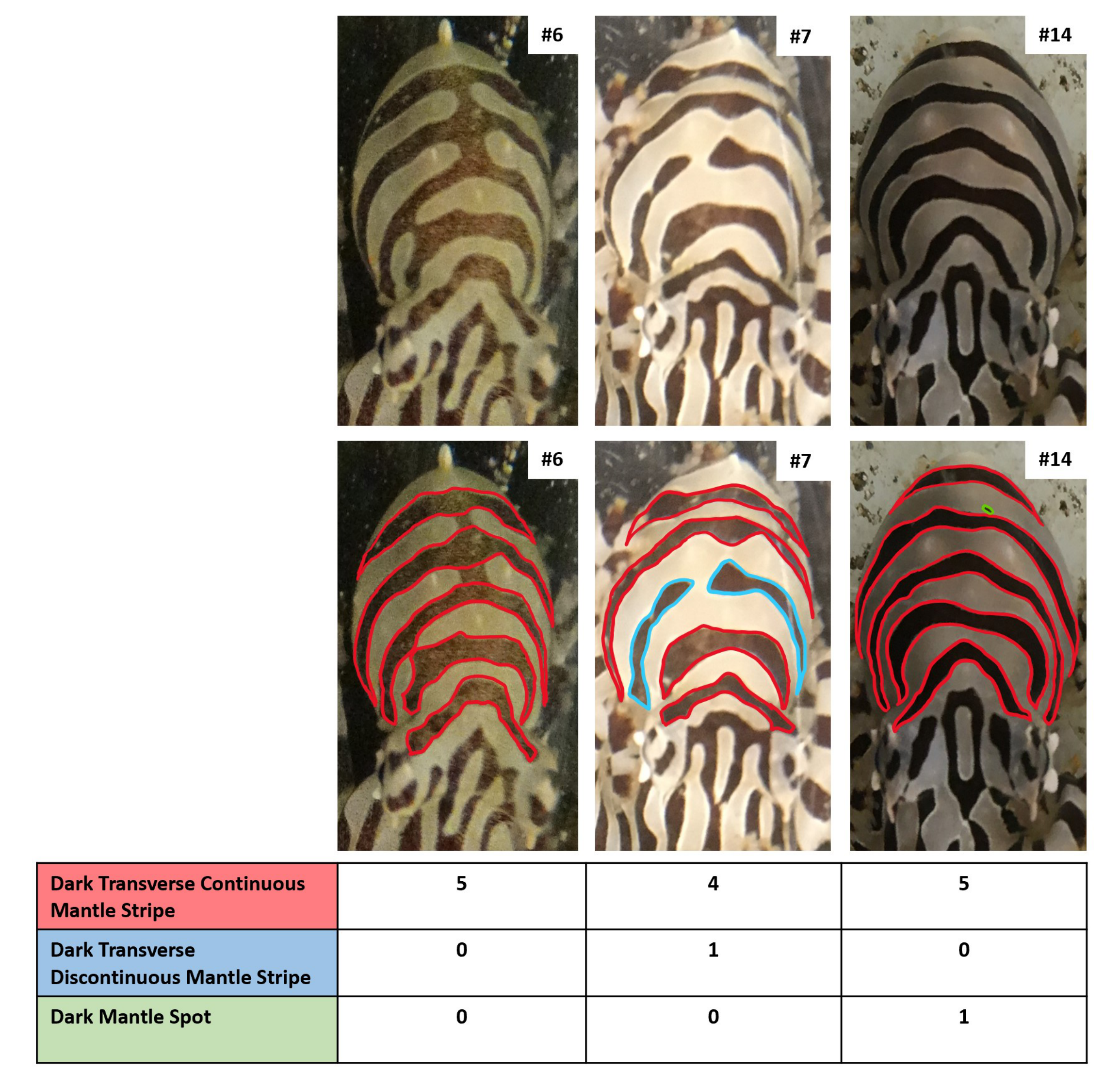

Supplement: S1 Fig — (TIFF) [file pone.0265292.s009.tiff]
