# Supplementary material for: Individually unique, fixed stripe configurations of Octopus chierchiae allow for photoidentification in long-term studies
Source: PLoS One. 2023 Apr 12;18(4):e0265292. doi: 10.1371/journal.pone.0265292 (PMC10096297; doi:10.1371/journal.pone.0265292)
Supplement: Attachment — Submitted filename: Response to Reviewers.pdf [file pone.0265292.s010.pdf]

8 October 2022

Dear Dr. Erik V. Thuesen,

Thank you for your attention to our manuscript, and the constructive comments from the reviewers. We are grateful for the opportunity to address the issues with our initial draft. In addition to our revised manuscript and figure files, we also submit the manuscript file with track changes incorporated, for your reference if needed (Revised Manuscript with Track Changes.*docx*). However, we note that our revisions included extensively revised survey design, data analysis, and text reorganization, and as a result, it may be difficult to visualize individual changes.

Reviewer #1:

1. Liu and colleagues investigated if body patterns in Octopus chierchiae are individually unique and consistent over time and if untrained volunteers can distinguish individuals of this species from pictures. Wild collected octopus were reared and mated in laboratory. A subset of the F2 hatchlings was raised in laboratory and photographed weekly for two years. Untrained volunteers were subjected to an online survey showing pairs of pictures with and without superimposed pattern tracers to test if they were able to distinguish among different individuals. The authors concluded that O. chierchiae showed individually distinctive stripes patterns that are stable through most of its lifespan, opening to the possibility of using them for field photoidentification without animal manipulation, and that non-experts can distinguish among specimens successfully when a clear view of body patterns is provided.

This study addresses the crucial issue of enabling studies on animals without compromising their welfare and I highly appreciate the effort of establishing a minimally/not invasive identification method. Such an approach can pave the way to numerous, exciting applications in this and other species that are currently largely overlooked due to the technical challenges associated with handling fragile organisms. Despite my interest and positive attitude towards such a kind of studies, I would encourage the authors to be clearer and more detailed in their methodological descriptions.

In the current form, I am unable to evaluate if the chosen methodology is appropriate and I have concerns on data analysis and interpretation. If the points indicated below are addressed, then it would be easier to understand their experimental and analytical choices, the presented results, their interpretation and hopefully they will help in making the conclusions and manuscript more solid.

Reply:

We appreciate the reviewer's comments on our manuscript. We have incorporated further details about our methods and analysis in accordance with the reviewer's suggestion and hope that our revision has made our study more interpretable.

2. "Aims and Methods: to my understanding and as anticipated above, this study: tests 1) if stripes patterns can be used as individual identifiers across lifetime in this species; and 2) if untrained volunteers can distinguish between individuals through photos.

First, it seems to me that the uniqueness and consistency of body patterns over time was not tested in an explicit framework. The main text did not mention any statistical analyses to test these hypotheses (uniqueness and consistence, which are two separate features that should not be confused), only a reference to personal observations and pictures (lines 201-204). These results need to be supported by a more solid and objective evidence on the distinctiveness and stability of patterns. For example, individual and time-series pictures could be compared through computer-assisted photo identification software or a survey like the one for untrained volunteers performed on experts. Why were identification software not considered in this study nor in future perspectives? They could be helpful in discerning patterns."

Reply:

We appreciate the comments and concerns raised by the reviewer.

Because access to this species is extremely limited, there is not a significant population of experts outside of our own research team available to perform the survey on and compare with the results from untrained volunteers.

Although interesting, computer vision and other such computer-assisted photoidentification analytics were beyond the scope of expertise and resources of this research team at the time of the study. We proposed such studies making use of this technology be conducted on *O. chierchiae* in the future:

Lines 302-304: "Smartphone apps or websites can be utilized to gather responses *en masse* and the process may even be automated via computer-assisted photoidentification software with enough responses and high quality image data."

Furthermore, there is not an existing extensive repository of photographs for this species, making training computer-assisted photo identification tools unfeasible at the time. Often machine learning libraries rely on thousands to hundreds of thousands of images, and even then are not necessarily as effective as humans in finding matches.

However, inspired by the methods of Huffard et al. (2008) and Byrne et al. (2010), we performed traces of *O. chierchiae* stripe configurations over extensive time spans [Fig 4] and found that the pattern traces remained the same, as was the case for a single *Wunderpus photogenicus* tracked over time (Huffard et al. 2008). For this reason, we believe the conclusion that *O. chierchiae* have unique and consistent stripe configurations over time we reached with our study is valid and legitimate.

Lines 195-199: “For the four individuals with the most extensive photobanks, we arranged a series of photos chronologically, one per month if available, and traced their stripe configurations using Adobe Illustrator. Each isolated stripe was color coded [Fig 4] to visualize and establish the consistency of the stripe configurations. ”

3. Second, it is not clear to me why the authors are interested on photoidentification from untrained volunteers specifically. Was it a control to avoid potential overestimation from trained experts? If this was the case, it should be written explicitly.

Reply:

We have since added this statement explicitly:

Lines 245-246: “Our study focused on untrained volunteers specifically to control for potential overestimation from trained experts with daily exposure to the animals.”

4. According to the main text (lines 108-109, 178-189, 204-213, 216-218, 246-256) it seems that the authors are mainly considering some kind of a citizen science project. Such projects largely differ in type of collected data, required expertise, bias, etc. For example, a project where an untrained volunteer sees an octopus while swimming, she/he takes a photo, sends it together with geographic coordinates taken from smartphone to an expert (i.e., the volunteer provides raw data that is then analyzed by an expert) is different from a project where a person is simply asked if she/he saw the same specimen (i.e., the volunteer “analyses” data on her/his own). The test to explore if a citizen science project can be effective should consider and reflect these differences, starting from how pictures are taken and who analyze them (volunteer? Expert?). More detailed information on the envisaged contribution of untrained volunteers should be added and the analyses valuated accordingly, I am largely unable to assess them in the current form.

Reply:

We have provided a detailed description of a potential way untrained volunteers would be able to contribute to community science efforts in monitoring and studying the species:

Lines 304-310: “We envision untrained volunteers engaging in community science efforts by contributing to the establishment of a photodata repository. This could be done by sending a photograph in which the stripe configuration of the animal’s mantle’s dorsal surface is visible along with the date, time, and geographic coordinates saved from a smartphone. Future research teams may make use of such a database and draw inferences about the species’ natural history, including home range, behavior, population size, population density, and wild lifespan.”

5. Lines 154-162: more details on how samples were photographed are needed to understand the study design. For example, why were not all the hatched individuals photographed? Why was a sample size of 25 chosen for this analysis? How were the photographed samples selected?

Reply:

We have included a more detailed description of our study design under the Photography Methods section of our manuscript:

Lines 180-184: "Due to high hatchling and juvenile mortality rate (Grearson et al., 2021) and some individuals' aversion to open environments, only a subset [n = 25] of our lab reared populations were involved in this study. Only individuals with sufficiently long lives whose activity patterns allowed frequent visibility of stripe configurations to be clearly photographed on multiple occasions were included in this subset."

Lines 218-222: "We created a survey following the methods of Huffard et. al. (2008) using the photobank of images taken over the course of our study. The survey [S4 File] consisted of a series of 20 slides, arranged by a random number generator, each depicting two photographs of *O. chierchiae* individuals side by side, with no repeated measurements. Photo pairs were rotated and cropped to display the animals in similar orientations and sizes."

6. Lines 144-146: mentioned multiple clutches and Figure 4 indicates different hatching time (November 2018, May 2019), were any differences in body pattern uniqueness and consistency tested among clutches, hatching time and parents?

Reply:

We have since included the following statement:

Lines 163-164: "Comparisons of individuals from different clutches, hatching times, and parents were not tested in this study."

7. Were the cameras underwater or emerged? Both settings should be tested if both possibilities could occur in the citizen science project, and the limits associated with such techniques should be discussed (e.g., a picture taken by an emerged camera might be distorted due to refraction and waves).

Reply:

We have clarified in Line 178: "We photographed individual *O. chierchiae* from above water…"

We appreciate the concern raised by the reviewer. However, expert advisors with field experience (Dr. Christine Huffard) have suggested that no practical difference

would arise from emerged or submerged cameras. Because these octopuses are so small, the camera would need to be very close in order to take a picture, and waves/refraction would not be an issue. Additionally, since we are primarily concerned with the stripe configuration of the animals specifically, distortion due to refraction and waves would be of minimal concern. Even if the length and widths of each stripe is changed, the stripe configuration will be the same. Finally, waves and refraction were not issues in the study of photoidentification in W. photogenicus (Huffard et al. 2008), which relied heavily on photographs taken underwater by professional and recreational divers.

8. Were pictures standardized (e.g., taken from the same height through a copy stand) and repeated at each session and view?

Lines 158-160 not fully clear: how many pictures were taken for each individual and session? One top view plus two side view pictures?

Reply:

We have clarified in Lines 191-195: “Over the course of our study, we attempted to avoid disturbing and distressing the animals during feeding, tank transfer, photography sessions, etc.. If an individual was sheltering or clinging to the jar lid, they were not photographed that session. One picture per individual per session was taken from angles relative to the animal’s body position at the time of viewing in order to capture the octopus’ dorsal mantle surface as clearly as possible. “

9. Lines 179-185: how many slides were shown to participants? Were slides designed to be viewed in a computer monitor or smartphone? Was a zoom function, possibility to go back and edit a response or time limit included? Were entries randomized (e.g., to avoid showing all matches first)? Were repeated measurements (e.g., presenting the same photos pair multiple times) included?

Reply:

We have since provided more details about the survey design of our revised survey.

Lines 219-222: “The survey [S4 File] consisted of a series of 20 slides, arranged by a random number generator, each depicting two photographs of *O. chierchiae* individuals side by side, with no repeated measurements. Photo pairs were rotated and cropped to display the animals in similar orientations and sizes. ”

Lines 228-229: “No time limit or built-in zoom function was implemented and participants were free to go back and edit responses prior to submission. .”

Lines 234-237: “In our social media solicitation, we reminded participants to complete the survey on a computer screen, as designed. However, because our data collection methods were not face-to-face and were analyzed anonymously, we cannot

confirm if all the survey responses were completed on a computer screen or on any other device"

10. Lines 181-182: why the maximum age gap between the two pictures in the same slide was five months? Did the zero days apart photos represent the repeated pictures of the same individual on the same day with the same settings or different ones (e.g., pictures taken at different time, or a side and a top view)?

Reply:

Photo pairings for the survey were chosen by a random number generator. As a result, the maximum five month age gap between two pictures in our original survey was completely arbitrary and random.

Zero days apart was intended to represent photographs of two different individuals taken on the same day. To avoid confusion, the "zero days apart" description has since been deleted.

We have clarified our photography methods in Line 193-194: "One picture per individual per session was taken…"

11. Lines 185-189: while more attention was dedicated to the survey description, what and how the collected responses were analysed is less clear. For example, the meaning of "compiled" and "graded", and most importantly, the hypotheses that were tested using the t-tests (please explicitly mention it and its methodological reference), the compared groups and the analyzed data/variables are not obvious. Was data analysed anonymously? The t-test is a parametric approach with specific assumptions to meet but it looks like that these were not tested. If dichotomous data was analyzed, the t-test might not be the most appropriate approach.

Reply:

We have clarified the descriptions of our collected responses:

Lines 231-234: "Each set of survey responses was given a score $[\frac{correct\ answers}{total\ questions} \times 100]$. Incorrect answers were categorized as false positive (meaning an erroneous match) and false negative (meaning a missed match). Unanswered questions were scored as incorrect, but omitted in our false positive-false negative analysis."

Survey results data were analyzed anonymously, as described in Lines 235-237: "However, because our data collection methods were not face-to-face and were analyzed anonymously, we cannot confirm if all the survey responses were completed on a computer screen or on any other device."

We have removed the t-test from our study as, per reviewer comment, it may not be appropriate for our data and adds little to the overall interpretation of our results. In its place we simply looked at the mean and median survey scores to draw our conclusions.

Additionally, we have since clarified the hypothesis in Lines 257-259: “We hypothesized that participants would be able to identify *O. chierchiae* individuals based on their stripe configurations, meaning they would score notably above 50%, the expected value if responses were chosen randomly.”

12. Lines 205-239: the number of surveyed untrained volunteers (22) seems to be quite low. How long was the survey open? How was the sample size chosen?

Reply:

In our newly revised survey, 38 untrained volunteers participated in the survey.

The survey was promoted through social media solicitation for two weeks and was kept open for another two weeks for a total of a month as described in Lines 224-226: “We solicited participants via social media for two weeks, and collected responses via Google Forms for a total of four weeks.”

The survey response sample size was not not chosen intentionally. We simply included all the survey responses that we received over the span of a month (the timespan which the survey was open for) and incorporated them into our study. We believe that a sample size of 38 is adequate and sufficient as similar survey based studies have included far fewer participants (Huffard et al. 2008; Byrne et al. 2010).

13. Such surveys can be biased by demographic factors such as participants’ age, sex, education, occupation, geographic provenance, etc. Was this type of information collected for each participant? Responses should be evaluated in the light of these factors. Issues in vision and the use of a computer vs. smartphone screen might explain some inter-individual variance in responses too. Additionally, the fact that some participants responded to both surveys (line 239) introduce a potential bias that need to be considered.

Reply:

The survey was completely anonymous and no personal information of the participants was collected.

We agree with the concern raised by the reviewer regarding the use of differently sized electronic screens impacting participant’s survey response and have discussed it at length in Lines 234-237: “In our social media solicitation, we reminded participants to complete the survey on a computer screen, as designed. However, because our data collection methods were not face-to-face and were analyzed anonymously, we cannot confirm if all the survey responses were completed on a computer screen or on any other device.”

In our new round of surveys, only one survey format is available, thus eliminating the potential bias introduced from participants responding to both of our original surveys.

14. Lines 216-226: in addition to the concerns highlighted above, I do not think that the conclusion that this species can be consistently identified by untrained observers can be supported so strongly without at least testing if the percentage of correctly assigned matches increases when the issues identified in lines 221-223 are fixed. An explicit comparison between non-expert and expert performance and a measurement of identification accuracy would be more informative too. These analyses would make this study substantially more solid and comprehensive.

Reply:

In our newly designed survey, all of the photographs selected were in-focus pictures of octopuses in their light morph with their stripe configurations on their mantle fully exposed and visible, thus eliminating the issues present in our previous survey.

Like the reviewer, we were also profoundly intrigued by the idea of an explicit comparison between non-expert and expert performance and a measurement of identification accuracy. However, due to the incredible difficulty accessing the species, few individuals in the world have had extensive and sufficient experience working with the animal to be considered experts. And since all of us, the authors, participated in organizing the photos and designing the survey, we were not suitable to participate in the survey ourselves as “experts”.

15. Line 242-246: this study potentially (please see my comments about testing uniqueness and consistency of body patterns above) showed that stripe patterns are stable over time in captivity in a fully standardized rearing environment since before birth. Could variable field conditions and injuries affect body patterns? This and similar potential limits of these results should be discussed.

Reply:

We recognize the limitation described by the reviewer and have since included the following detail:

Lines 189-192: “Per our observations, environmental disturbances can stimulate a change in the animal’s color morph, from dark to light, light to dark [Fig 2a], or flashing between morphs [Fig 2b]. Over the course of our study, we attempted to avoid disturbing and distressing the animals during feeding, tank transfer, photography sessions, etc.. ”

Lines 293-297: “It is conceivable that environmental disturbances and injuries may affect the stripe configuration of *O. chierchiae* in the wild. However, since so little is known about the species in its natural habitat and we avoided injuring animals as much as possible over the course of this study, we do not have conclusive evidence whether or not environmental conditions and injuries would affect an animal’s stripe configuration.”

16. Lines 220-226: the "specific examination of the problematic questions" should be described in more details. How were the factors underlying the problematic questions identified? Was their influence explicitly tested or just hypothesised?

Reply:

The factors underlying the problematic questions were hypothesized based on common attributes and characteristics within their corresponding photographs. The influence of these hypothetical factors underlying the problematic questions were not explicitly tested.

These hypothesized problematic questions were completely removed in our newly designed survey.

17. Ethics statement: several key information such as the field permit number (or if this is not needed or it is included in the formal waiver already), formal waiver number and details on animal welfare are missing. Research projects involving human participants includes the use of data collection methods which are not face-to-face such as online surveys unless they are analysed anonymously, but it is not clear if this was case. The appropriate details should be added here and in Materials and Methods as indicated by the journal.

Reply:

We have since added the following statement explicitly:

Lines 143-145: "An official statement from the UC Berkeley OLAC is provided in the supplementary materials [S1 File]. Human participants were recruited via social media and all data was analyzed anonymously."

Lines 235-239: "However, because our data collection methods were not face-to-face and were analyzed anonymously…"

<u>Minor comments</u>

18. Line 31: here and in the rest of the text, I would be more specific in indicating that body patterns refer to stripes' configurations, as this study did not consider other features such as skin texture.

Reply:

We have implemented the change in terminology per reviewer recommendation. We have replaced all relevant mentions of "body pattern" with "stripe configuration".

19. Line 34: the untrained volunteers survey was conducted on laboratory-reared animals, not wild ones. Please rephrase this sentence and the rest of the main text accordingly. Please also consider replacing “demonstrate” with “might suggest”.

Reply:

We have implemented the change in terminology.

Lines 35-37: “These results demonstrate that laboratory-reared individuals could be identified via photographs taken at different points in their lifetime, which might suggest wild individuals can also be recognized and observed for field longitudinal studies.”

20. Line 87-88: I would suggest replacing “innocuous” with “non-invasive”. I agree that data collection through photoidentification is less invasive than handling, but it likely still involves some stress, being observed/photographed by someone can be an unpleasant experience for humans too. I would avoid using strong words such as “harmless”, “non-intrusive” and instead indicate that such techniques are less impactful on animal welfare.

Reply:

We have implemented the change in terminology per reviewer comment.

21. Line 35, 92, 251, 263: “in situ“ should be in italics. Alternating or replacing it with “field” might be clearer to readers.

Reply:

We have implemented the change in terminology per reviewer suggestion. We have replaced some of the mentions of “in situ” in the manuscript with “field”.

22. Line 102: please add more recent references for photo identification methods.

Reply:

We have since included more recent citations within the past five years.

(Johnston et al., 2017; Schneider, 2019; Vidal, 2021)

Johnston DR, Rayment W, Slooten E, Dawson SM. A time-based method for defining associations using photo-identification. Behaviour. 2017;154(9–10):1029–50.

Schneider S, Taylor GW, Linquist S, Kremer SC. Past, present and future approaches using computer vision for animal re-identification from camera trap data.

Methods Ecol Evol. 2019;10(4):461–70. Available from: http://dx.doi.org/10.1111/2041-210x.13133

Tan HY, Goh ZY, Loh K-H, Then AY-H, Omar H, Chang S-W. Cephalopod species identification using integrated analysis of machine learning and deep learning approaches. PeerJ. 2021;9(e11825):e11825. Available from: http://dx.doi.org/10.7717/peerj.11825

Vidal M, Wolf N, Rosenberg B, Harris BP, Mathis A. Perspectives on individual animal identification from biology and computer vision. Integr Comp Biol. 2021;61(3):900–16. Available from: http://dx.doi.org/10.1093/icb/icab107

23. Line 134: what is the age of sexual maturity?

Reply:

We have since included the following sentence:

Lines 156-158: "As gonad maturity is not currently defined in cephalopods, we approximate the age indicative of sexual maturity to be 16 weeks old, based on behavioral observations, i.e. engaging in copulation."

24. Lines 140-141: please explain the relevance of analysing a generation bred and raised in captivity.

Reply:

We have since included the following sentence explaining the relevance of analyzing a generation of animals bred and raised in captivity:

Lines 167-170: "While the exact age and influence of life experiences on the development of wild caught individuals cannot be determined, having study animals bred and raised entirely in captivity allowed us the unique opportunity to follow the species' development throughout its entire lifecycle, hatching till death."

25. Lines 141-146: do these numbers refer to the F2 alone or include the F1?

Reply:

This number refers to both F1 and F2 combined.

We have since removed all references to generations (F0, F1, F2) from the main body of text, as per reviewer comments, and moved these more detailed breeding and rearing information to the Supplementary Materials [S7 Supporting Information].

26. Lines 136-137: are all mates from the F1 generation?

Reply:

No, not all mated pairs were from the F1 generation. Some breeding pairs were chosen including wild caught individuals in order to reduce inbreeding. We have removed all reference to generations (F0, F1, F2) from the main body of text, as per reviewer comments, and moved these more detailed breeding and rearing information to the Supplementary Materials [S7 Supporting Information].

27. Lines 179-180: please specify if "this photobank" refers to pictures collected in this study or in the mentioned reference.

Reply:

Photobank refers to the pictures collected in this study

Lines 218-219: "We created a survey following the methods of Huffard et. al. (2008) using the photobank of images taken over the course of our study."

28. Line 185: the pattern tracers were superimposed, not below the photo. Please be aware that some visually impaired (e.g., colour-blind) people might have difficulties in seeing them.

Reply:

The pattern traces referenced in this reviewer comment were not superimposed.

We understand and acknowledge that some visually impaired people may have difficulties in seeing the pattern traces. In the future, a research effort with more dedicated time and resources would ideally use color-blind friendly palettes to create the pattern traces, thus making them more accommodating for those who are visually impaired.

29. Lines 191-198: the main text, specifically the Methods, should include this important information that is currently disclosed only these captions, i. e., the pictures pairs showed either the same individual on different days or different individuals on the same day.

Reply:

Due to us having replaced our previous surveys with a newly designed survey, all the captions referenced in this comment and their associated figures have since been removed from the manuscript.

30. Lines 202-204: please specify if experts were able to recognize individuals thanks to the uniqueness or/and consistency of body patterns.

Reply:

At the time of this study, there were no established experts on this species aside from our research group, that we know of. We have since included the following clarification in our manuscript:

Lines 242-244: “All members of our research group (the authors of this paper) were able to recognize individual animals based on a combination of the uniqueness and consistency of the octopuses’ stripe configurations.”

31. Line 240: the survey C was not mentioned before.

Reply:

Survey C has since been removed from our study.

32. 267-268: please report all the information in Methods, including the age at which photo recording started. Why were animals not photographed until they were at least four weeks old?

Reply:

We have since included this information to the Methods section:

Lines 186-189: “The animals were not photographed until they were at least four weeks old because that is the earliest age when every individual’s stripe configuration became clearly discernible on the phone cameras we used to take the photos.”

33. Discussion: I would be more cautious in using expressions such as “our surveys confirm” (line 244), “suggest” would be more appropriate.

Reply:

We have implemented the recommended change in terminology by replacing “confirm”with “suggest” in the referenced line.

34. Line 255: “en masse” should be in italics.

Reply:

We have implemented the recommended correction.

35. Lines 250-287: I enjoyed reading these potential applications, which included a width of perspectives that should be more common in methodological studies.

Reply:

We sincerely appreciate the reviewer's kind words and comments.

36. Figure 1 and 2 caption: the species' name should not start with a capital letter and the complete name should be in italics, as reported elsewhere in the manuscript.

Reply:

We have implemented the correction per reviewer comment.

37. Figure 1-2 and 6-9: I would suggest grouping these pictures in fewer figures (e.g., Figure 1 and 2 could become a single one with multiple panels).

Reply:

We have implemented the suggested changes in figure formats.

We have combined Figure 1 and 2 into Figure 1a and 1b.

Figure 6-9 of our original manuscript submission have been removed entirely. Our redesigned survey may be found in our Supplementary Materials [S4 File].

38. Figure 10 and 11: these figures and captions mentioned "scores" that were not explained in the main text. Please describe how these "scores" were obtained from the surveys' responses in Methods.

Reply:

We have since included the following clarification:

Lines 231: " Each set of survey responses was given a score [$\frac{correct\ answers}{total\ questions} \times 100$]"

39. The number of figures is quite high and might overwhelm readers. I would suggest focusing on few, most relevant ones and moving the others to Supplementary.

Reply:

We have implemented the recommended change by the reviewer and reduced the number of figures by focusing only on a few relevant figures in the main body of text and moving the rest to Supplementary Materials or removing them entirely.

40. Data availability: please include a statement in the main text too and specify what type of data is available.

Reply:

We have since included the following statement detailing the type of data that is available:

252-253: "Survey data can be found in the Supplementary Materials [S5 Dataset]."

41. Numbers from zero to ten (in some cases to thirteen) are conventionally written using words and not numbers.

Reply:

We have implemented the change according to the reviewer comment.

<u>Reviewer #2:</u>

1. The idea behind this paper is worthwhile yet the scholarship overall is subpar and there is one major problem. Both can be remedied if the authors concur - and want their research to be used and further developed by others. I provide examples below and give suggestions on how to address these. For example, the authors provide a very slanted choice of references to "support" their paper (sample details below).

The major flaw in the paper is that the authors do not understand that leucophores in the dermis are the basis of these permanent and species-specific white markings; in this case of O. chierchiae they are also the basis of individual specificity. Leucophores are not even mentioned in this paper.

Here is the basic scenario: chromatophores are mostly evenly distributed in the skin and when selectively activated create pigmentary patterns. Iridophores and leucophores (i.e. "white cells") are not evenly distributed; they occur only in specific parts of the skin. Leucophores are aggregates of static cells (they do not have nerves, muscles etc) and are common in nearly all

octopuses and cuttlefish. Andrew Packard and his colleagues as well as Roger Hanlon and colleagues have published widely on this and you need to reference some of those papers so that readers understand where this "fingerprinting" comes from. (For example: Packard, A., & Hochberg, F. G. (1977). Skin patterning in Octopus and other genera. Symposia of the Zoological Society of London, 38, 191-231.)

Both of those authors have shown that innervation patterns of chromatophores are aligned to expand adjacent to leucophore white markings to produce the high-contrast zebra-like markings. This was demonstrated neurophysiologcally and anatomically, for example, in Sepia officinalis (see Figs in Hanlon and Messenger 1988, Phil Trans). Conversely, other neural command neurons activate chromatophores directly over the leucophores to diminish (or mask) their bright whiteness.

By the way, leucophores produce some of the whitest white known in the animal kingdom (Mäthger, L. M., Senft, S. L., Gao, M., Karaveli, S., Bell, G. R. R., Zia, R., . . . Hanlon, R. T. (2013). Bright white scattering from protein spheres in color changing, flexible cuttlefish skin. Advanced Functional Materials, 23(32), 3980-3989) so this precise pigmentary masking by chromatophores is important and O chierchiae do this as demonstrated by your images.

Your Supplementary Fig. 10 in file S2_file.pdf shows this very well !! This figure has to be elevated to the main manuscript to illustrate what I explained above.

Reply:

> We thank the reviewer for the information they have provided. We respectfully believe the topic of skin ultrastructure is beyond the scope of this study, and not necessary for the interpretation of our results. All of the skin components involved in octopus body color patterns are anatomically fixed in their location. While chromatophores are certainly involved, we do not feel it is necessary or appropriate to speculate about the degree to which iridophores and leucophores may or may not also be involved.
>
> We have included a summary of the recommended information and citations:
>
> Lines 120-124: "Cephalopod body patterns are produced by a combination of pigment-filled chromatophores, color-reflective iridophores, and passively reflective leucophores, as well as muscular and hydrostatic forces that produce textural details (How et al., 2017; Mäthger et al., 2009) that operate within the fixed anatomical architecture of an octopus' skin (Packard and Hochlberg, 1977; Messenger, 2001) [Fig 2]."
>
> We have also moved Supplementary Fig. 10 from our original S2_file.pdf into the main body of text in our revised manuscript and renamed it Fig. 2b.

<u>Specific Comments:</u>

INTRODUCTION

2. Line 41 - delete this sentence - it adds nothing to the paper and such a statement applies to thousands of papers. Furthermore, the reference for this is inappropriate as it is written by someone who only studies octopuses.

Reply:

We have implemented this change and deleted the sentence.

3. L 47 the Hackett reference only covers the pop culture side of what is stated in this sentence. For the biological attributes supply a global rigorous study review such as Hanlon and Messenger 2018 book Cephalopod Behaviour or similar.

Reply:

We have since included a more recent scientific review article covering the same topic as the recommended book (discussing scientific interest in octopuses).

Di Cosmo A, Pinelli C, Scandurra A, Aria M, D'Aniello B. Research trends in octopus biological studies. Animals (Basel). 2021;11(6):1808. Available from: http://dx.doi.org/10.3390/ani11061808

4. L49. There are numerous studies have tracked individual cephalopods in the wild over time. It does not matter that it is difficult. You should cite 2-4 such studies - that have been done on octopuses, cuttlefishes, squids and nautilus!

(One of many examples: Hanlon, R. T., Forsythe, J. W., & Joneschild, D. E. (1999). Crypsis, conspicuousness, mimicry and polyphenism as antipredator defences of foraging octopuses on Indo-Pacific coral reefs, with a method of quantifying crypsis from video tapes. Biological Journal of the Linnean Society, 66(1), 1-22. Retrieved from ://000078601200001

Reply:

We have since included additional citations of studies tracking individual cephalopods in the wild overtime, more recent than the one the reviewer recommended.

Brewer RS, Norcross BL, Chenoweth E. Temperature- and size-dependent growth and movement of the North Pacific giant octopus (Enteroctopus dofleini) in the Bering Sea. Mar Biol Res. 2017;13(8):909–18.

Byrne RA, Wood JB, Anderson RC, Griebel U, Mather JA. Non-invasive methods of identifying and tracking wild squid. Ferrantia. 2010;59:22–31.

5. Overall this intro paragraph is heavily slanted and even a bit misleading. Kindly pose the study in a more appropriate and professional manner.

Reply:

We appreciate the reviewer's comment and have since removed several components of the introduction paragraph that may be perceived as misleading. We have also since included numerous citations, as per reviewer comments, to further establish the validity and professionalism of our writing.

6. L 57. An appropriate reference for cephalopod semelparity is Rocha, F., Guerra, A., & Gonzalez, A. F. (2001). A review of reproductive strategies in cephalopods. Biological Reviews, 76(3), 291-304. doi:10.1017/s1464793101005681

Reply:

We have since added the recommended citation in Line 57.

7. L59 This statement of "… apart from most other octopus species" is nonsense. A very large number of octopus species produce very large eggs that hatch out as miniature adults (i.e. no paralarval/planktonic stage). The authors seem unaware of cephalopod life histories.

Reply:

Because the majority of octopus species with large eggs that hatch out as miniature adults are deep sea forms, we have made the following specification:

Lines 60-62: "Another trait that sets *O. chierchiae* apart from the majority of other shallow water octopus species (Sweeney et al., 1992) is its lack of a planktonic stage in its development, suggesting a limited potential for dispersal."

We have also included the following citation detailing diverse cephalopod life histories to support our claim.

Sweeney MJ, Roper CFE, Mangold KM, Clark MR, Boletzky SV. "Larval" and juvenile cephalopods: a manual for their identification. Smithson Contrib Zool. 1992;(513):1–182. Available from: http://dx.doi.org/10.5479/si.00810282.513

8. L66 The Grearson et al 2021 is not even published … it is BioRivX … Moreover, this species is anything but a model organism! There are only a handful of journal papers and the species is nearly impossible to obtain from the wild, and only recently have lab trials begun in earnest. This sort of hyperbole is unacceptable.

Reply:

Grearson et al. 2021 has since been published.

9. L 102 There are literally dozens of papers in which cephalopods have been studied with photography; at least cite a few of them to justify this paper and this Introduction.

Reply:

We have since included the following citations of cephalopods being studied with photography.

Drerup C, Jackson A, Rickard C, Skea M, Cooke GM. Field observations on the behavioural ecology of the stout bobtail squid Rossia macrosoma (Cephalopoda: Sepiolidae) from Scottish waters. Mar Biodivers. 2021;51(4). Available from: http://dx.doi.org/10.1007/s12526-021-01202-y

Vecchione M, Roper CFE. Cephalopods observed from submersibles in the western North Atlantic. Bull Mar Sci. 1991;49(13):433-45.

Roper CFE, Hochberg FG. Behaviors and systematics of cephalopods from Lizard Island, Australia, based on color and body patterns. Malacologia. 1988;29(1):153-93

Tan HY, Goh ZY, Loh K-H, Then AY-H, Omar H, Chang S-W. Cephalopod species identification using integrated analysis of machine learning and deep learning approaches. PeerJ. 2021;9(e11825):e11825. Available from: http://dx.doi.org/10.7717/peerj.11825

10. L 104 Another method to follow individual octopus, squid and cuttlefish is scar tissue in the skin, or missing arm(s), or even marking the skin with dye in specific spots. These need to be mentioned for proper context in this paper.

Reply:

We have since included details on these alternative methods in the following lines:

Lines 84-88: "Tattooing and branding techniques have had some success in cephalopod tracking studies as they eliminate the issue of tag removal and allow for small animals to be tagged (Semmens et al. 2007). However, they are often practically difficult to execute and may cause the animal severe distress and damage (Semmens et al. 2007)."

Lines 114-117: "Longitudinal field studies conducted on the Caribbean reef squid, *Sepiotheuthis sepioidea*, have demonstrated that individual identification in the wild via body patterning and markings from injury (i.e. skin scar tissue, missing arms, miss-built arms), can be viable in cephalopods (Byrne et al., 2010)."

METHODS

11. This section if very long and includes many items that have nothing to do with this study. If you want credit for reading this species then do a separate ms and put in an appropriate

mariculture journal. Otherwise shorten this section substantially. There are way too many figures in the main ms; if you choose to include so much culture info (which is not the biological basis of this paper) then at least put them in Supplementary sections.

Reply:

We have made the recommended reductions and moved a substantial amount of animal husbandry information and figures to the Supplementary Materials section [S2 Supporting Information]. We have also removed our survey figures and added references to a Supplementary file [S4 File] containing our entire survey.

RESULTS

12. L 201. How many individual octopuses were studied? The only hint of this is in the captions of Figs 7-9; from these the estimate would be perhaps as few as 2 octopuses. This is of central importance and the authors have to be crystal clear on this.

Reply:

The sample size of 25 individual octopuses is already stated in Lines 30-31: “photographically documenting the physical development of 25 octopuses from hatching” and Line 181 “[n = 25]”.

We have reiterated this once more in the results section of our revised manuscript for greater clarity:

Line 240: “[n = 25]”

13. L 203 Where are the data on the results of the research group (this number has to be clear - do you mean the 5 coauthors?)

Reply:

To clarify our intent, the statement in our manuscript referenced by this reviewer comment is purely observational. We have since clarified that we mean the 5 coauthors. Considering that all 5 members contributed to the breeding and rearing of the animals as well as to photography and survey design, we did believe we could survey ourselves without introducing bias.

Line 242: “All members of our research group (the authors of this paper)…”

14. Fig. 10 text/number letters are way too small. The y axis title of Percent of Total Score is ambiguous - can you provide a more intuitive title so that the graphic can stand alone in terms of understanding? … to that point, a color legend could/should be put directly on the graph … there is ample space amidst the blue bars.

Reply:

We have made the suggested formatting changes.

15. An important omission: you need to verify that the white stripes are leucophores and that their distribution is distinctive. The simplist fast method is to do some Light microscopy of gross anatomy of the leucophore stripes. This will give authenticity to your study. You can also illustrate this by taking a skin sample and photograph it normally then with light behind it to show that the leucophores stand out and block light in specific patterns. See Figs 14,15 i (Hanlon, R. T., & Messenger, J. B. (1988). Adaptive coloration in young cuttlefish (Sepia officinalis L.): The morphology and development of body patterns and their relation to behaviour. Philosophical Transactions of the Royal Society of London B, 320, 437-487. Note also Figs 18,19 the same paper to show how chromatophore expansion either accentuates or masks the whiteness.

Reply:

We appreciate the reviewer's comment and have since made the additions below to our manuscript. Please also see our reply to the other leucophore comment above.

Line 120-124: "Cephalopod body patterns are produced by a combination of pigment-filled chromatophores, color-reflective iridophores, and passively reflective leucophores, as well as muscular and hydrostatic forces that produce textural details (How et al., 2017; Mäthger et al., 2009) that operate within the fixed anatomical architecture of an octopus' skin (Packard and Hochlberg, 1977; Messenger, 2001) [Fig 2]."

A microscopic study of the cellular makeup of the animal's skin may be possible for other research groups, but adding this component would be well beyond the scope of this study, and would require sacrificing our study subjects. It is also not necessary to understand skin ultrastructure in order to understand the photoidentification process and give authenticity to this study (see Huffard et al. 2008). We have included the following line encouraging future interested researchers to pursue this study subject in the future:

Lines 325-329: "Identifying the specific anatomical composition of *O. chierchiae*'s stripe configuration was beyond the scope of our study, and would have required sacrificing our study subjects. We encourage future research efforts to be directed into examining the skin histology and ultrastructure of *O. chierchiae,* when deceased individuals are available, in order to gain further insight into the physiological basis of their stripe configuration development."

DISCUSSION

16. L 217. These results are not very convincing - 75% correct is only fair to begin with, but even then only a bit more than half even scored that high.

Reply:

As per reviewer #1's comment, we have modified our survey, correcting for problems that arose during our preliminary survey and attained new, more convincing results.

Lines 247-249: "Survey respondents (n = 38 responses) had a mean score = 84.2% with standard deviation = 15.36% and standard error of the mean = 2.49%. Survey respondents had a median score of 90%, with 44.74% of respondents (n = 17) scoring 95% or higher. [Fig 6]."

17. Moreover, the whole treatise seems to be based on 2 (or a very few) octopus individuals. (See comments in Results; perhaps there is a better explanation about how many octopuses were analyzed in this study).

Reply:

25 individual octopuses were used in this study, as stated in Lines 30-31: "photographically documenting the physical development of 25 octopuses from hatching" and Line 181 "[n = 25]".

We have reiterated this once more in the results section of our revised manuscript for greater clarity for future readers

Line 240: "[n=25]"

18. L 225 "when a clear view of the body patterns is provided." This is a large caveat to the study - your images in the lab were not all that useful/clear either, so going on later to say this could be useful in the field is a quantum leap in difficulty and utility.

Reply:

We sincerely appreciate the reviewer for pointing out that this original sentence did not convey what we were intending to express accurately. We have since included the following clarification:

Lines 275-276: "when an unobstructed, roughly in-focus view of the stripe configuration on the mantle's dorsal surface is provided."

This caveat is in line with the typical quality control any researcher would conduct on their raw image data.

19. Overall, it appears that using such body patterns is quite difficult and requires considerable study and diligence by the biologists. The authors never really say this in this paper. Need to be more straight up on what the results actually indicate.

Reply:

We respect and appreciate the concern raised by the reviewer. Based on the other inputs that we have received from the reviewers, we have since redesigned our survey. And the results of this redesigned survey are indicative that using stripe configurations are fairly easy and straightforward. Untrained volunteers attained high scores, with over 71% of survey respondents scoring 80% or higher on the survey, and a median score of 90%.

20. L 250+ Applying this technique to following individuals underwater will be very difficult (this reviewer has studied many octopus species in the wild; with many volunteers). The photography techniques in situ will require great diligence and patience to even get sufficient images to verify identity, much less acquire quantitative behavioral data.

Reply:

We recognize and appreciate the concern raised by the reviewer. While we acknowledge that the application of this technique may be difficult for some researchers, one of our co-authors (Dr. Roy Caldwell) and one of our resources (Dr. Christine Huffard) have extensive experience successfully photographing and tracking wild individual octopuses underwater. They confirm that many researchers, and far more underwater photographers (both professional and amature) have sufficient skills to take these photographs. One need only conduct a google image search for “Blue Ringed octopus” or the smaller, far more cryptic “Hairy octopus” to appreciate the incredible talent in the underwater photography community. Thus we affirm that it is reasonable to believe such methods would be a viable pursuit for sufficiently skilled researchers, especially who are willing to form collaborations with underwater photographers (e.g. Huffard et al. 2008) if access to *O. chierchiae’s* habitat is available.

21. L 244 To say you have no evidence of pattern change over time is a strong statement given how few animals were used.

Reply:

Based on reviewer #2’s comment 12 and 17, this reviewer seems to be under the impression that our study only consisted of 2 individuals. We have clarified that 25 animals were tracked over the 2 year course of this study..

22. L 245-249 Workers with some octopus and cuttlefish species have often used White frontal spots in octopus and details of zebra stripes in Sepia officinalis as species markers, and I believe J Boal made a comment on individual identity in S. officinalis in one of her pubs (you can search that one). Better off to give broad story and include leucophore markings in other cephs not just your octopus species.

Reply:

We have added the recommended citation:

Lines 288-291: “Although individual recognition has been explored in other cephalopods (Boal, 2006; Shashar 2004; Tricarico et al., 2011), to our knowledge, this is the first evidence that individually unique stripe configurations can be used by volunteers to consistently identify the same individual octopus throughout its development, from juvenile to adult.”

Boal J. Social recognition: a top down view of cephalopod behaviour. Vie et Milieu - Life & Environment. 2006;56(2):69-79.

Shashar N, Vaughan K, Loew E, Boal J, Hanlon R, Grable M. Behavioral evidence for intraspecific signaling with achromatic and polarized light by cuttlefish (Mollusca: Cephalopoda). Behaviour. 2004;141(7):837–61. Available from: http://dx.doi.org/10.1163/1568539042265662

Tricarico E, Borrelli L, Gherardi F, Fiorito G. I know my neighbour: individual recognition in Octopus vulgaris. PLoS One. 2011;6(4):e18710.

23. This paper has potential but requires major revision. These comments are given in a constructive manner to improve the science.

Reply

We have taken these comments into consideration during our revisions.

Once again, we appreciate your time and attention as well as the constructive comments from the reviewers. In every case we have incorporated the recommended change or clarified our intentions and reasoning.

Sincerely,

Leo Song

University of California, Berkeley: Department of Integrative Biology

3040 Valley Life Sciences Building # 3140

Berkeley, CA 94720-3200

leosong@berkeley.edu

(510) 570-5304
