# Supplementary material for: Individually unique, fixed stripe configurations of Octopus chierchiae allow for photoidentification in long-term studies
Source: PLoS One. 2023 Apr 12;18(4):e0265292. doi: 10.1371/journal.pone.0265292 (PMC10096297; doi:10.1371/journal.pone.0265292)
Supplement: Attachment — Submitted filename: Response to Reviewers.docx [file pone.0265292.s011.docx]

3 March 2023

Dear Dr. Erik V. Thuesen,

We sincerely appreciate your time and attention to our manuscript, and the constructive comments from the reviewer. We are grateful for the opportunity to address the issues with our resubmission. In addition to our revised manuscript and figure files, we also submit the manuscript file with track changes incorporated, for your reference if needed (Revised Manuscript with Track Changes*.docx*).

**Editor’s Comments:**

1) Please read over the comments of the reviewer, and address their concerns or explain why you don't.

**Reply:**

We have read over the comments of the reviewer and have addressed their concerns or provided our explanations to the best of our ability.

2) The reviewer asks you to identify patterns as per the categories of Packard & Hochberg. Is there some unique character as described in that paper that people are using to discern individuals?

**Reply:**

The pattern categories outlined in Tables III and IV of Packard and Hochberg (1977) include: White Mantle Spots, White Head-Bar, Frontal White Spots, Arm White Spots, Dark Arm Bar, Eye-Bar, Extended Eye-Bar, Dark Eye-Ring, Eyes-Spot (ocellus), Four Long Mantle Papillae, and Other. People may use some or all of these pattern categories, or something else entirely, to discern individual *O. chierchiae*.

As stated in Packard and Sanders (1971): “...if we ask ‘How many patterns are there in the octopus?’ the best, though hardly satisfactory, answer is ‘There are as many patterns as can be recognized by the classifier’ [...] the primary observational datum is the pattern, not the parts of which it is made up of." (Packard and Sanders, 1971)

We have included references to Packard and Hochberg (1977) as well as Packard and Sanders (1971), to provide readers with more classic terminology for observed pattern components. However, per Packard and Sanders (1971), we believe that attempts to verbalize what each person may use as unique characters to discern individuals would be a pointless endeavor.

Lines 197-200: “...pattern is generally composed of brown and white head bars, eye bars, extended eye bars, and transverse (chevron) stripes on the head and mantle, and longitudinal stripes on the arm crown. Occasionally, small brown mantle dark spots can be found within a white mantle stripe, and vice versa. The precise shape of each of these components was variable between individuals ”

3) In the first submission, you had two trials. In this paper, you have a third trial, but you have thrown out all your previous data. I don't understand how you can do that. Was that third trial run this past year after you received the reviews for this manuscript? Were any of the individuals 'taking the test' the same? That would mean they had more practice. Minimally, that information needs to be included in the appendix.

**Reply:**

The third trial run this past year was performed after we had received the reviews for our initial manuscript submission. This was done in consideration of the reviewer comments recommending us to refine our survey and see if the results improve.

We neglected to include this information in our previous submission and have now created Supplemental Material S5 and S6 where we provide the original surveys, results, description of the problems found, and how we improved upon them for the redesigned trial reported in the main body of the paper.

We recognize the concern that if the same individuals were to take the survey again, they may have additional practice and thus bias the result. Consequently, in our social media solicitation, we publicized and advertised this new survey on social media platforms and channels not used in our previous trials specifically to reach out to people who had not completed the previous two surveys. We also noted in the solicitation message requesting people who had taken the previous two surveys not to participate in this third redesigned survey. As a result, we have reason to believe that none of the individuals taking the test were the same.

4) Please be more specific about solicitation via social media.

**Reply:**

We reached out to Slack channels and Facebook groups that do not contain individuals who had participated in the previous two surveys and requested volunteers to complete our third revised survey. These Slack channels and Facebook groups range from our workplace channels to hobbyist groups that are not biology or science oriented in order to minimize bias in participant backgrounds. Additionally, we specifically stated in our solicitation message asking people who had taken the previous two surveys not to participate in this third redesigned survey in an effort to eliminate bias from additional practice.

We further specified our social media solicitation methods in Lines 141-144: “Human participants were recruited via social media by reaching out in Slack channels and Facebook groups. These social media channels ranged from our workplace channels to hobbyist groups that are not biology or science oriented in order to minimize bias from participant backgrounds.”

5) The reviewer asks for "corroborating the results with other evidence". One way to compare your results would be to have trained observers take the same test. You state "All members of our research group (the authors of this paper) were able to recognize individual animals based on a combination of the uniqueness and consistency of the octopuses stripe configurations", but did you ever test that?

**Reply:**

All authors of this paper took the survey and were able to recognize individual animals with 100% accuracy. We did not explicitly test this as we were all involved in the creation of the survey itself, which makes taking the survey ourselves an uninformative and unrigorous endeavor.

Additionally, as we mentioned in our previous review response, due to the extremely limited access to *O. chierchiae*, there are simply too few people in the world who are adequately trained and experienced with the species outside of our own research group to take the survey as “trained observers” and make a meaningful comparison with the results of untrained volunteers.

6) In Huffard et al. (2008), the conclusion was that people needed training in order to accurately identify individuals. Your results corroborate that conclusion, yet you state "to our knowledge, this is the first evidence that individually unique stripe configurations can be used by volunteers to consistently identify the same individual octopus throughout its development, from juvenile to adult." I would disagree with your statement. Rather, training is needed to reach an acceptable level (99.9%) of identification. I'm not sure why you have overstated your results, but that could be one reason the reviewers have asked for so much corroborating evidence to support your conclusions.

**Reply:**

In Huffard et al. (2008), the authors explicitly stated in their discussion: "Untrained observers were able to differentiate individual *W. photogenicus* based on photographed body patterns." The results of our study corroborate that conclusion.

In addition, we do not know of any formal standard that defines the acceptable level of photoidentification as 99.9% accurate. In fact, nearly all of the published photoidentification studies involving untrained observers that we examined reported less than 99.9% accuracy, usually involving a smaller sample size of volunteers than ours as well (Huffard et al., 2008; Cooley et al., 2013; Chaves et al., 2016; Treilibs et al., 2016; Theimer et al., 2017). The expectation of 99.9% accuracy is unrealistic and not representative of working expectations in actual photoidentification studies. By contrast, marine animal photoidentification projects involving machine learning often provide a correct match less than 80% of the time, and yet can still be very useful (Katija et al., 2022).

Huffard CL, Caldwell RL, DeLoach N, Gentry DW, Humann P, MacDonald B, et al. Individually unique body color patterns in octopus (*Wunderpus photogenicus*) allow for photoidentification. PLoS One. 2008;3(11):e3732.

Cooley CR, Smith SA, Geier CJ, Puentes TG. The use of photo-identification as a means of identifying Western Painted Turtles (*Chrysemys picta bellii*) in long-term demographic studies. Herpetol. Rev. 2013;44(3):432–434.

Chaves LCT, Hall J, Feitosa JLL, Côté IM. Photo-identification as a simple tool for studying invasive lionfish Pterois volitans populations: Pterois volitans photo-identification. J Fish Biol [Internet]. 2016;88(2):800–4. Available from: http://dx.doi.org/10.1111/jfb.12857

Treilibs CE, Pavey CR, Hutchinson MN, Bull CM. Photographic identification of individuals of a free‐ranging, small terrestrial vertebrate. Ecol Evol [Internet]. 2016;6(3):800–9. Available from: http://dx.doi.org/10.1002/ece3.1883

Theimer TC, Ray DT, Bergman DL. Camera Angle and Photographic Identiﬁcation of Individual Striped Skunks. Wildl. Soc. Bull. 2017;41(1):146–150.

Katija K, Orenstein E, Schlining B, Lundsten L, Barnard K, Sainz G, et al. FathomNet: A global image database for enabling artificial intelligence in the ocean. Sci Rep [Internet]. 2022;12(1):15914. Available from: http://dx.doi.org/10.1038/s41598-022-19939-2

7) Overall the manuscript is too wordy. It can be made more succinct.

**Reply:**

We recognize this concern and have since made the writing of our manuscript more succinct where possible.

8) See the instructions for PLOS ONE figure legend style. <https://journals.plos.org/plosone/s/figures#loc-captions>

**Reply:**

We have since corrected the formatting of our figure legends according to the instructions provided.

9) Please make sure that figures are numbered in the order they appear in the text. For example, your current order for figure three is incorrect (C comes before A and B).

**Reply:**

We have since corrected this mistake and all of the figures are now numbered in the order they appear in the text.

10) Give volumes for the chambers in figure three legend.

**Reply:**

We have now provided the volumes for the chambers in Figure 3’s legend.

11) Please stick with third person in the acknowledgements.

**Reply:**

We have since corrected this mistake and now use third person exclusively in the acknowledgments.

12) In the literature cited section, please put all the genus/species names in italics.

**Reply:**

We have since corrected this mistake and have put all the genus/species names in the literature cited section in italics.

**Reviewers' comments:**

Reviewer's Responses to Questions

**Comments to the Author**

1. If the authors have adequately addressed your comments raised in a previous round of review and you feel that this manuscript is now acceptable for publication, you may indicate that here to bypass the “Comments to the Author” section, enter your conflict of interest statement in the “Confidential to Editor” section, and submit your "Accept" recommendation.

Reviewer #1: (No Response)

2. Is the manuscript technically sound, and do the data support the conclusions?

The manuscript must describe a technically sound piece of scientific research with data that supports the conclusions. Experiments must have been conducted rigorously, with appropriate controls, replication, and sample sizes. The conclusions must be drawn appropriately based on the data presented.

Reviewer #1: Partly

3. Has the statistical analysis been performed appropriately and rigorously?

Reviewer #1: N/A

4. Have the authors made all data underlying the findings in their manuscript fully available?

The [PLOS Data policy](http://www.plosone.org/static/policies.action#sharing) requires authors to make all data underlying the findings described in their manuscript fully available without restriction, with rare exception (please refer to the Data Availability Statement in the manuscript PDF file). The data should be provided as part of the manuscript or its supporting information, or deposited to a public repository. For example, in addition to summary statistics, the data points behind means, medians and variance measures should be available. If there are restrictions on publicly sharing data—e.g. participant privacy or use of data from a third party—those must be specified.

Reviewer #1: Yes

5. Is the manuscript presented in an intelligible fashion and written in standard English?

PLOS ONE does not copyedit accepted manuscripts, so the language in submitted articles must be clear, correct, and unambiguous. Any typographical or grammatical errors should be corrected at revision, so please note any specific errors here.

Reviewer #1: Yes

6. Review Comments to the Author

Please use the space provided to explain your answers to the questions above. You may also include additional comments for the author, including concerns about dual publication, research ethics, or publication ethics. (Please upload your review as an attachment if it exceeds 20,000 characters)

Reviewer #1: I recognize the efforts of Authors in addressing Reviewers comments and suggestions. However, I found the revised version of the manuscript still with some pitfalls that does not put me in the condition to recommend for publication at this stage. I believe in the Authors view, that individuals of O. chierchiae exhibit unique patterns that allow identification of each individual animal. However, the approach and data provided do not support such an evidence with adequate robustness. The option of independent observers, survey is only one possibility and needs to be corroborated by other approaches.

**Reply:**

Based on several of the sources that we had examined (Bryne et al. 2010; Theimer et al., 2017), we believe that only pursuing one approach of surveying independent observers is sufficient for this initial study of the species.

The only other paper we know of on octopus photoidentification (Huffard et al. 2008, published in PLOSONE) was based on fewer individuals studied over a much shorter duration. We used similar photoidentification methods to those outlined in that paper, but involved a larger number of volunteers. Performance of our study subjects was comparable to that of Huffard et al. 2008.

However, we appreciate the concern raised by the reviewer and have included Figure 4: a display of a single representative photograph of each individual included in our survey study; with accompanying S7 Dataset: a count of different pattern components captured by photograph and observed by our research team on the dorsal mantle surface of each individual. This figure and table provide additional evidence of the uniqueness of *O. chierchiae* patterns.

Byrne RA, Wood JB, Anderson RC, Griebel U, Mather JA. Non-invasive methods of identifying and tracking wild squid. Ferrantia. 2010;59:22–31.

Theimer TC, Ray DT, Bergman DL. Camera Angle and Photographic Identiﬁcation of Individual Striped Skunks. Wildl. Soc. Bull. 2017;41(1):146–150.

Huffard CL, Caldwell RL, DeLoach N, Gentry DW, Humann P, MacDonald B, et al. Individually unique body color patterns in octopus (*Wunderpus photogenicus*) allow for photoidentification. PLoS One. 2008;3(11):e3732.

As major issues:

1. I am unable to find a description of the different patterns exhibited by the individuals studied (and included in this ms), i.e. a description of patterns (chronic I assume, sensu Packard and Hochberg, 1977 - in the classic definition of body patterning of cephalopods, that the Authors seem to not consider at its fully extent). These descriptions need to be corroborated by some statistics (descriptive and correlation, at least). There is the need of bringing to the Readers objective evidence on the distinctiveness and stability of patterns observed.

**Reply:**

We appreciate the reviewer’s comments and have since included descriptions of the different patterns exhibited by the individuals studied in the following lines:

Lines 189-200: “Terms used to describe *O. chierchiae* body patterns follow those defined and illustrated previously (Packard and Sanders, 1969; Packard and Sanders, 1971; Packard and Hochberg, 1977, Caldwell et al., 2015). Morphological appearance of each animal may vary greatly depending on body position or chromatophore activation. Consequently, this investigation focused on the stripe configurations of the chronic patterns located on the animals’ dorsal mantle surface [Fig 4A], because these were the most consistently observable identifying characteristics. Chronic patterns are those that persist for hours or days, or those that the animal returns to after acute variations that last seconds or minutes (Packard and Sanders, 1969). The stripe-bar-spot pattern is generally composed of brown and white head bars, eye bars, extended eye bars, and transverse (chevron) stripes on the head and mantle, and longitudinal stripes on the arm crown. Occasionally, small brown mantle dark spots can be found within a white mantle stripe, and vice versa. The precise shape of each of these components was variable between individuals.”

We have since incorporated Figure 4: a display of a single representative photograph of each individual included in our survey study; with accompanying S7 Dataset: a count of different pattern components captured by photograph and observed by our research team on the dorsal mantle surface of each individual. These further showcase the uniqueness of *O. chierchiae* body patterns.

We have also applied descriptive statistics as objective corroborating evidence using the S7 Dataset referencing Vaissi et al., (2018).

Lines 200-207: “We chose to specifically examine [S7 Dataset] the dark transverse mantle stripes and dark mantle spots in the chronic patterns of *O. chierchiae*. We defined dark transverse mantle stripes as any dark pattern component that traverses the width of the individual’s mantle. We further categorized these into dark continuous stripes [Fig 4B] and dark discontinuous stripes [Fig 4C], those that contained no visible breakage and those that contained visible breakage, respectively. We also defined dark mantle spots [Fig 4D] as any dark pattern component completely encircled by white pattern components on an individual’s mantle.”

Lines 269-273: “The *O. chierchiae* bred and raised in our lab displayed [Fig 4] an average of 4.8 (±0.49 S.D.) dark transverse mantle stripes in their chronic patterns. In 20% (n =5) of the animals, this included one discontinuous stripe [Fig 4C]. Additionally, 24% (n =6) of the animals also displayed a dark mantle spot [Fig 4D] [S7 Dataset]. Examples of how pattern component counts were obtained may be found in Supplementary Materials [S8 Figure].”

Correlation statistics are not applicable in our study. As we observed stable and consistent stripe configurations over time, there are not two variables to draw a correlation between. For example, if the number of stripe components remains consistent, that would be a constant. By the statistical definition of correlation, there must be two variables to consider. Attempts to apply correlation statistics such as Pearsons or Spearman's correlation resulted in undefined correlations. However, we believe that our timeline figures [Fig 5] adequately demonstrate *O. chierchiae*’s body pattern stability over extended time spans.

Vaissi S, Farassat H, Akmali V, Sharifi M. Consistency of coloration pattern and applicability of photo identification method as a tool to identify individuals of the kaiser’s mountain newt, *Neurergus kaiseri*. Russ J Herpetol. 2018;25(4):311.

2. I understand (from the comments to Reviewers) that the Authors prefer not to use computer assisted photo identification softwares at this stage, but there are other approaches that may allow to support their findings.

**Reply:**

We have since incorporated Figure 4: a display of a single representative photograph of each individual included in our survey study; with accompanying S7 Dataset: a count of different pattern components captured by photograph and observed by our research team on the dorsal mantle surface of each individual. These further showcase the uniqueness of *O. chierchiae* body patterns.

We have also applied descriptive statistics as objective corroborating evidence using the S7 Dataset referencing Vaissi et al., (2018).

Lines 200-207: “We chose to specifically examine [S7 Dataset] the dark transverse mantle stripes and dark mantle spots in the chronic patterns of *O. chierchiae*. We defined dark transverse mantle stripes as any dark pattern component that traverses the width of the individual’s mantle. We further categorized these into dark continuous stripes [Fig 4B] and dark discontinuous stripes [Fig 4C], those that contained no visible breakage and those that contained visible breakage, respectively. We also defined dark mantle spots [Fig 4D] as any dark pattern component completely encircled by white pattern components on an individual’s mantle.”

Lines 269-273: “The *O. chierchiae* bred and raised in our lab displayed [Fig 4] an average of 4.8 (±0.49 S.D.) dark transverse mantle stripes in their chronic patterns. In 20% (n =5) of the animals, this included one discontinuous stripe [Fig 4C]. Additionally, 24% (n =6) of the animals also displayed a dark mantle spot [Fig 4D] [S7 Dataset]. Examples of how pattern component counts were obtained may be found in Supplementary Materials [S8 Figure].”

We believe that our timeline figures [Fig 5] adequately demonstrate *O. chierchiae*’s body pattern stability over extended time spans.

We believe that the information presented in Figure 4, Figure 5 and the descriptive statistics, in conjunction with our survey results, provide the readers with adequate objective evidence of the distinctiveness and stability of *O. chierchiae* patterns which are identifiable by even untrained volunteers.

3. Thus, there is the need of corroborating the results with other evidence apart from the outcome from the survey circulated among untrained volunteers. This is only providing part of the evidence that Authors want to confirm their view.

**Reply:**

We appreciate the reviewer’s comment. However, we believe that the survey results by untrained volunteers provide adequately robust support to our conclusion that *O. chierchiae*’s unique stripe configurations allow individual animals to be identified and distinguished by untrained observers as the same approach has been used to draw similar conclusions in *Wunderpus photogenicus, Chrysemys picta bellii, Sepiotheuthis sepioidea, Mephitis mephitis,* and etc. (Huffard et al., 2008; Bryne et al. 2010; Cooley et al., 2013; Theimer et al., 2017).

We have since incorporated Figure 4: a display of a single representative photograph of each individual included in our survey study; with accompanying S7 Dataset: a count of different pattern components captured by photograph and observed by our research team on the dorsal mantle surface of each individual. These further showcase the uniqueness of *O. chierchiae* body patterns.

We have also applied descriptive statistics as objective corroborating evidence using the S7 Dataset referencing Vaissi et al., (2018).

Lines 200-207: “We chose to specifically examine [S7 Dataset] the dark transverse mantle stripes and dark mantle spots in the chronic patterns of *O. chierchiae*. We defined dark transverse mantle stripes as any dark pattern component that traverses the width of the individual’s mantle. We further categorized these into dark continuous stripes [Fig 4B] and dark discontinuous stripes [Fig 4C], those that contained no visible breakage and those that contained visible breakage, respectively. We also defined dark mantle spots [Fig 4D] as any dark pattern component completely encircled by white pattern components on an individual’s mantle.”

Lines 269-273: “The *O. chierchiae* bred and raised in our lab displayed [Fig 4] an average of 4.8 (±0.49 S.D.) dark transverse mantle stripes in their chronic patterns. In 20% (n =5) of the animals, this included one discontinuous stripe [Fig 4C]. Additionally, 24% (n =6) of the animals also displayed a dark mantle spot [Fig 4D] [S7 Dataset]. Examples of how pattern component counts were obtained may be found in Supplementary Materials [S8 Figure].”

We believe that our timeline figures [Fig 5] adequately demonstrate *O. chierchiae*’s body pattern stability over extended time spans.

We believe that the information presented in Figure 4, Figure 5 and the descriptive statistics, in conjunction with our survey results, provide the readers with adequate objective evidence of the distinctiveness and stability of *O. chierchiae* patterns which are identifiable by even untrained volunteers.

Huffard CL, Caldwell RL, DeLoach N, Gentry DW, Humann P, MacDonald B, et al. Individually unique body color patterns in octopus (*Wunderpus photogenicus*) allow for photoidentification. PLoS One. 2008;3(11):e3732.

Cooley CR, Smith SA, Geier CJ, Puentes TG. The use of photo-identification as a means of identifying Western Painted Turtles (*Chrysemys picta bellii*) in long-term demographic studies. Herpetol. Rev. 2013;44(3):432–434

Byrne RA, Wood JB, Anderson RC, Griebel U, Mather JA. Non-invasive methods of identifying and tracking wild squid. Ferrantia. 2010;59:22–31.

Theimer TC, Ray DT, Bergman DL. Camera Angle and Photographic Identiﬁcation of Individual Striped Skunks. Wildl. Soc. Bull. 2017;41(1):146–150.

4. Text in Lines 266-276 (and relevant figures) should be moved to Results

**Reply:**

We have implemented the reviewer suggestion and moved the text in Lines 266-276 and any relevant figures to the Results section.

Lines 290-297: “To investigate if there was a significant difference between false positive and false negative rates in the survey response, we performed the Wilcoxon Signed Rank Test with our null hypothesis being that false positive rates and false negative rates were the same, while our alternative hypothesis being that false positive rates and false negative rates were different. The test result revealed that there was not a significant difference between false positive and false negative rates (Wilcoxon Signed Rank Test: test statistic = 150, α = 0.05, n = 28, critical value = 116), suggesting a potential future study of wild populations would not be biased towards one error type or another.”

5. Sentence at Lines 288-291 is misleading and scientifically inaccurate. Authors wrote "Although individual recognition has been explored in other cephalopods (Boal, 2006; Shashar 2004; Tricarico et al., 2011), to our knowledge, this is the first evidence that individually unique stripe configurations can be used by volunteers to consistently identify the same individual octopus throughout its development, from juvenile to adult". Individual recognition is a biological phenomenon, based on a cognitive feature of some animals, and used to describe the evidence that a given individual animal is capable of identifying a conspecific. This is not the case of this study. Authors, a different species, are using untrained volunteers (and the same is claimed in the text and in the comments to Reviewers, to be the case of caretaker and people involved in this study) - again a different species -, to identify individuals belonging to the target species. What Authors are adopting is a biometric identification of individual octopuses, based on features of chronic (or stable) patterns exhibited by the same animal over time.The sentence should be rephrased or deleted.

**Reply:**

We acknowledge that we had mistakenly misused the term “individual recognition” in Line 288-291 of our previous submission. We have since re-phrased this sentence and have moved the contained references into a more appropriate section of our manuscript:

Lines 232-235: “To our knowledge, this is the first evidence that individually unique stripe configurations can be used by volunteers to consistently identify the same individual octopus throughout its development, from juvenile to adult”

Lines 361-363: “Individual recognition has been explored in several other cephalopods (Shashar 2004; Boal, 2006), and at least one species, *Octopus vulgaris*, is thought to have the ability of individual conspecific recognition (Tricario et al., 2011). ”

In addition, in several instances the Authors do not rely on accurate use of in-text citation, or the use of not adequate references is preferred to better and more classic/authoritative citations. For example:

Line 46 - aspects of the biology of the species/cephalopods are available in e.g., FAO volume 3 for octopuses (Jereb, P., Roper, C., Norman, M., and Finn, J. (2016). Cephalopods of the World. An Annotated and Illustrated Catalogue of Species Known to Date. Volume 3. Octopods and Vampire Squids. Roma, Italy: FAO, Food and Agriculture Organization of the United Nations). The citations included are not adequate, to my view and expertise.

**Reply:**

We appreciate the reviewer’s comment. However, in an effort to make our manuscript more concise, as per the editor’s suggestion, we have removed the text in question entirely.

Similarly at Line 48 - Authors have various useful, authoritative options better than the two utilized and this Reviewer may eventually provide better suggestion.In many other instances the citations provided are only a very limited example of the possibilities. Thus, Authors may use e.g., for each of the in-text citations (otherwise do not supporting adequately the sentences).

**Reply:**

We appreciate the concern raised by the reviewer and have since utilized e.g. for many of our in-text citations. However, in an effort to make our manuscript more concise, as per the editor’s suggestion, we have removed some of the texts in question entirely.

In the specific instance at Line 48, the citations included in this sentence were intended to refer to broad overviews of the wide array of possibilities which exists as part of our introductory hook. Therefore, we believe that the references provided here are adequate for our purposes.

Line 58 - citation of Mather (2006) is useless and does not add any other information to the knowledge provided by the very good review by Rocha et al. (2001) that Authors also cite. The sentence should be rewritten and Mather (2006) is suggested to remove it from this context.

**Reply:**

We accept the reviewer’s suggestion and have since replaced the citation of Mather (2006) in Line 58 with a citation of the review by Rocha et al. (2001).

Line 75-76: “While most octopus species exhibit semelparity and only reproduce near the end of their lifespan (Rocha et al., 2001)...”

The paragraph (Lines 56-62) needs rewriting. Authors may consider adding other species as example (e.g., O. bimaculoides; O. maya) in the same condition of this one. The cited work (Sweeney et al., 1992) provide a tabularized list of species with hatchlings so-called miniature adults (linked to low number of eggs from mothers and large amount of yolk).

**Reply:**

We appreciate the reviewer’s comment. However, in an effort to make our manuscript more concise, as per the editor’s suggestion, we have eliminated the comparison of *O. chierchiae’s* reproductive strategy with those of other octopus species, as this is not the focus of our study.

Similarly, in text citations at Lines 80 and 100 require an e.g.

**Reply:**

We have since rewritten these lines to include relevant examples for their respective in text citations:

Lines 87-92: “Current studies on wild cephalopods most commonly involve tag-recapture studies (Semmens et al., 2007), as exemplified by efforts to investigate the growth and movement of *Octopus vulgaris* in Central Western Sardinia (Mereu et al., 2010) and *Enteroctopus dofleini* in the Bering Sea (Brewer et al., 2017). Visible Implant Elastomer tags have demonstrated viable usage in long term studies of larger cephalopods such as *E. dofleini* (Brewer and Norcross, 2012)”

Lines 104-109: “The low-impact nature of this approach as well as the virtual ubiquitousness of high-quality cameras that can record photographs, videos, geospatial data, and temporal data, has enabled community science efforts to make increasingly significant contributions to scientific research, as showcased by the US National Parks Service’s What’s Invasive! project and NSF NEON’s Project BudBurst (Graham et al., 2011; Dickinson et al 2012; Chandler et. al., 2017).”

Line 71. Authors refer to what is termed (appropriate definition) daily monitoring following Directive 2010/63/EU; see also requirements from ARRIVE Guidelines (refer for example to:

Kilkenny, C., Browne, W.J., Cuthill, I.C., Emerson, M., and Altman, D.G. (2010). Improving Bioscience Research Reporting: The ARRIVE Guidelines for Reporting Animal Research. PLOS Biology 8(6), e1000412. doi: 10.1371/journal.pbio.1000412.

Percie du Sert, N., Ahluwalia, A., Alam, S., Avey, M.T., Baker, M., Browne, W.J., Clark, A., Cuthill, I.C., Dirnagl, U., Emerson, M., Garner, P., Holgate, S.T., Howells, D.W., Hurst, V., Karp, N.A., Lazic, S.E., Lidster, K., MacCallum, C.J., Macleod, M., Pearl, E.J., Petersen, O.H., Rawle, F., Reynolds, P., Rooney, K., Sena, E.S., Silberberg, S.D., Steckler, T., and Würbel, H. (2020). Reporting animal research: Explanation and elaboration for the ARRIVE guidelines 2.0. PLOS Biology 18(7), e3000411. doi: 10.1371/journal.pbio.3000411.Fiorito, G., Affuso, A., Anderson, D.B., Basil, J., Bonnaud, L., Botta, G., Cole, A., D'Angelo, L., de Girolamo, P., Dennison, N., Dickel, L., Di Cosmo, A., Di Cristo, C., Gestal, C., Fonseca, R., Grasso, F., Kristiansen, T., Kuba, M., Maffucci, F., Manciocco, A., Mark, F.K., Melillo, D., Osorio, D., Palumbo, A., Perkins, K., Ponte, G., Raspa, M., Shashar, N., Smith, J., Smith, D., Sykes, A., Villanueva, R., Tublitz, N., Zullo, L., and Andrews, P.L.R. (2014). Cephalopods in neuroscience: Regulations, Research and the 3Rs. Invert. Neurosci 14, 13-36.

Fiorito, G., Affuso, A., Basil, J., Cole, A., de Girolamo, P., D'Angelo, L., Dickel, L., Gestal, C., Grasso, F., Kuba, M., Mark, F., Melillo, D., Osorio, D., Perkins, K., Ponte, G., Shashar, N., Smith, D., Smith, J., and Andrews, P.L. (2015). Guidelines for the Care and Welfare of Cephalopods in Research - A consensus based on an initiative by CephRes, FELASA and the Boyd Group. Lab. Anim. 49(2 Suppl), 1-90.The sentence should be rewritten

**Reply:**

We have since rewritten the sentence and have removed the term “monitor” from the text in question in our manuscript.

Lines 83-86: “Specifically, the ability to identify and observe the octopuses on an individual basis would be crucial for both field studies (Arechavala-Lopez et al., 2019) as well as controlled laboratory research involving multiple individual animals (Morse et al., 2017).”

7. PLOS authors have the option to publish the peer review history of their article ([what does this mean?](https://journals.plos.org/plosone/s/editorial-and-peer-review-process#loc-peer-review-history)). If published, this will include your full peer review and any attached files.

If you choose “no”, your identity will remain anonymous but your review may still be made public.

**Do you want your identity to be public for this peer review?** For information about this choice, including consent withdrawal, please see our [Privacy Policy](https://www.plos.org/privacy-policy).

Reviewer #1: No

Once again, thank you for your time and attention as well as the constructive comments from the reviewer. In every case we have incorporated the recommended change or clarified our intentions and reasoning.

Sincerely,

Leo Song

University of California, Berkeley: Department of Integrative Biology

3040 Valley Life Sciences Building # 3140

Berkeley, CA 94720-3200

leosong@berkeley.edu

(510) 570-5304
